# A leukemia-protective germline variant mediates chromatin module formation via transcription factor nucleation

**DOI:** 10.1038/s41467-022-29625-6

**Published:** 2022-04-19

**Authors:** Gerard Llimos, Vincent Gardeux, Ute Koch, Judith F. Kribelbauer, Antonina Hafner, Daniel Alpern, Joern Pezoldt, Maria Litovchenko, Julie Russeil, Riccardo Dainese, Riccardo Moia, Abdurraouf Mokhtar Mahmoud, Davide Rossi, Gianluca Gaidano, Christoph Plass, Pavlo Lutsik, Clarissa Gerhauser, Sebastian M. Waszak, Alistair Boettiger, Freddy Radtke, Bart Deplancke

**Affiliations:** 1 Institute of Bioengineering, School of Life Sciences, École Polytechnique Fédérale de Lausanne (EPFL), Lausanne, Switzerland; 2 Swiss Institute of Bioinformatics, Lausanne, Switzerland; 3 Swiss Institute for Experimental Cancer Research (ISREC), School of Life Sciences École Polytechnique Fédérale de Lausanne (EPFL), Lausanne, Switzerland; 4 Department of Developmental Biology, Stanford University, Stanford, CA USA; 5 Division of Hematology, Department of Translational Medicine, University of Eastern Piedmont, Novara, Italy; 6 Oncology Institute of Southern Switzerland, Università della Svizzera italiana, Bellinzona, Switzerland; 7 Institute of Oncology Research, Università della Svizzera italiana, Bellinzona, Switzerland; 8 Division of Epigenomics and Cancer Risk Factors, German Cancer Research Center (DKFZ), Heidelberg, Germany; 9 Centre for Molecular Medicine Norway (NCMM), Nordic EMBL Partnership, University of Oslo and Oslo University Hospital, Oslo, Norway; 10 Department of Pediatric Research, Division of Paediatric and Adolescent Medicine, Rikshospitalet, Oslo University Hospital, Oslo, Norway; 11 Present Address: Cancer Research UK Lung Cancer Centre of Excellence, University College London (UCL) Cancer Institute, Cancer Genome Evolution Research Group, London, UK

**Keywords:** Gene regulation, Chronic lymphocytic leukaemia

## Abstract

Non-coding variants coordinate transcription factor (TF) binding and chromatin mark enrichment changes over regions spanning >100 kb. These molecularly coordinated regions are named “variable chromatin modules” (VCMs), providing a conceptual framework of how regulatory variation might shape complex traits. To better understand the molecular mechanisms underlying VCM formation, here, we mechanistically dissect a VCM-modulating noncoding variant that is associated with reduced chronic lymphocytic leukemia (CLL) predisposition and disease progression. This common, germline variant constitutes a 5-bp indel that controls the activity of an *AXIN2* gene-linked VCM by creating a MEF2 binding site, which, upon binding, activates a super-enhancer-like regulatory element. This triggers a large change in TF binding activity and chromatin state at an enhancer cluster spanning >150 kb, coinciding with subtle, long-range chromatin compaction and robust AXIN2 up-regulation. Our results support a model in which the indel acts as an *AXIN2* VCM-activating TF nucleation event, which modulates CLL pathology.

## Abstract (web summary)

Non-coding variants can regulate transcription factor binding and gene expression at variable chromatin modules. Here, the authors show that a germline variant induces transcription factor nucleation through chromatin compaction leading to *AXIN2* up-regulation and is associated to better prognosis in chronic lymphocytic leukaemia.

## Introduction

A thorough understanding of how genetics contributes to complex traits or disease susceptibility is of great biomedical importance. Although the vast majority (~88–93%) of complex trait- or disease-associated single nucleotide polymorphisms (SNPs) are located outside gene coding regions^1,2^, only a handful of those have been studied at a mechanistic level. Classical views postulate that regulatory variation affects the interaction of transcription factors (TFs) with DNA, which locally affects gene expression and chromatin modifications^3^. However, only a small part of interindividual variable TF binding can be explained by sequence differences in the respective binding sites^4–9^. An intriguing hypothesis is that TFs are dependent on both short- and long-range collaborative interactions with other TFs or co-regulators^3^. This concept is consistent with the recent discovery that DNA regions within certain loci exhibit a high level of molecular coordination (histone marks, TF binding, chromatin accessibility, DNA methylation, and gene expression). We and others have captured this coordinated behavior in a statistical framework yielding variable chromatin modules (VCMs, also termed *cis*-regulatory domains (CRDs))^10–12^, which may be conceptually comparable to the notions of regulatory microenvironments^13^, chromatin nanodomains^14^ or (sub)-sub-topologically associated domains^15^. Further research has revealed that many of these VCMs may be subjected to an internal, regulatory hierarchy, involving “lead” *cis-*regulatory elements (CREs) that dictate the activity of “dependent” CREs, possibly by coordinating local chromatin contacts and chromosome conformation^16^. However, the nature of the genetic and/or molecular triggers that dictate such interactions has so far remained elusive, prompting the question of what molecularly drives VCM formation in the first place, and which role TFs and their target regulatory elements play in defining the activity and hierarchy of individual VCMs.

In this work, we took advantage of the VCM-based partitioning of the regulatory genome in lymphoblastoid cell lines (LCLs)^10,12,16^ to, first, identify and mechanistically detangle variants that control VCM activity, and second, explore whether such variants induce phenotypic effects in B cells given their anticipated large impact on surrounding molecular phenotypes^3,10^. These analyses led us to focus on the Axin-related protein 2 *(AXIN2)* locus which harbors a VCM that is composed of several CREs and whose activity, we found, is driven by the germline indel rs143348853.

## Results

### The rs143348853 indel acts as a QTL of the *AXIN2* VCM in LCLs

**Fig. 1:**
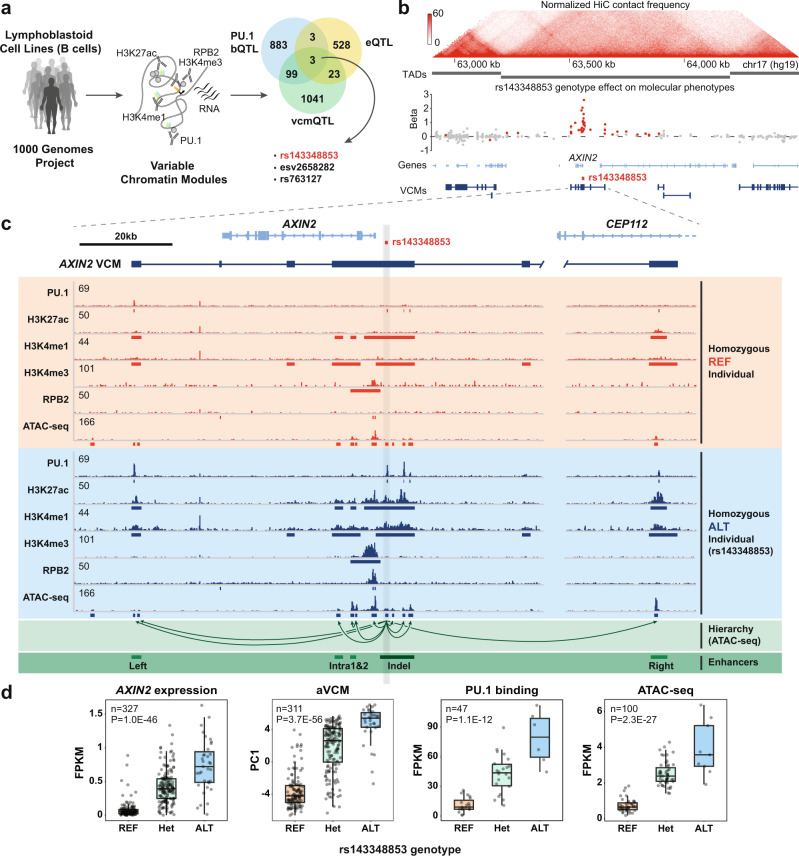
rs143348853 indel activates the *AXIN2* VCM in LCLs. **a** Workflow to evaluate impactful genetic variants using the VCM concept and Venn diagram of the significant variants considering an FDR ≤ 10% for VCM activity (vcmQTL), PU.1 binding (bQTL) and gene expression (eQTL). **b** (Top) Genomic view centered on the *AXIN2*-containing TAD using the 3D Genome Browser^110^ showing GM12878 HiC interactions from Rao et al., 2014^53^ and GM12878 TADs from Beekman et al., 2018^72^. (Bottom) Beta values representing the genetic effect of rs143348853 on different molecular phenotypes (PU.1, H3K27ac, H3K4me1, H3K4me3, RPB2 ChIP-seq, and ATAC-seq signals; FDR < 0.05 values are colored in red) and consensus VCMs. See Supplementary Fig. 1b for beta values for each molecular phenotype. **c** IGV^111^ view of the *AXIN2* VCM region showing the consensus *AXIN2* VCM and profiles for the studied molecular phenotypes from two different individual LCLs representing the rs143348853 homozygous REF and ALT genotypes. Meta peaks are displayed below each track. The shown regulatory hierarchy across the ATAC-seq peaks (visualized as unidirectional arrows) was inferred from the directed acyclic graph information from Kumasaka et al., 2018^16^. Finally, the five principal *AXIN2* LCL enhancer regions are annotated in green according to H3K4me1 and H3K27ac overlap, while the hypothesized “lead” enhancer is marked in dark green. **d** Boxplots showing different molecular phenotypes for each rs143348853 genotype in LCLs: *AXIN2* mRNA expression, the activity of the *AXIN2* VCM as measured by the first principal component analysis value (PC1) on all correlated histone marks embedded in the *AXIN2* VCM, and PU.1 binding (peak id 1036) and ATAC-seq signal (peak id 254430) for the rs143348853-overlapping peaks. *n* indicates the number of considered individual LCL samples and P indicates the *p-*value from a linear regression model. Boxes indicate the IQR (25–75%) and the box center indicates the median. Whiskers represent the minimum or maximum values of no further than 1.5 times the IQR for both the top and bottom of the box.

In this study, we set out to understand the molecular mechanisms underlying VCM formation and thus long-range molecular coordination. To do so, we first looked for genetic variants that control the activity state of VCMs using activity scores (aVCM) that were previously determined for each VCM from 47 individual LCLs^10^. Given the high number of variants that correlated with VCM activity scores (here, called VCM quantitative trait loci or vcmQTLs; 2580, FDR ≤ 10%), we further narrowed the list of candidates by searching for potential molecular phenotypic impact. Specifically, we searched for variants that acted on VCMs containing more than one CRE and that induced gene expression (eQTL) and TF (here, PU.1) binding variation (bQTL) (Fig. 1a). Only three variants fulfilled all criteria: the structural variant esv2658282 (*UGT2B17* eQTL), the SNP rs763127 (non-coding genes *Z97192.1* and *Z97192.2* eQTL) and the indel rs143348853 (*AXIN2* eQTL). We chose the latter for downstream mechanistic characterization given that it affected the expression of *AXIN2*, a well-studied gene that is known to be part of the Wnt signaling pathway and that has already been implicated in several pathologies including acute myeloid leukemia^17^, gastric carcinoma^18^, and oral^19^, lung^20^, prostate^21^, and colorectal^22^ cancers.

rs143348853 is a germline, non-coding, 5-bp-TCAAA deletion (indel) that is located ~2.5 kb upstream of the *AXIN2* transcription start site (TSS). Subsequent polarized insertion/deletion status analyses using the NCBI dbSNP^23^ (build 154) and a UCSC Genome Browser^24^ Multiz alignment of the indel locus to multiple vertebrate genomes (Supplementary Fig. 1a) allowed us to define the ancestral allele as the reference (REF) and the allele containing the TCAAA deletion as the alternate (ALT) with a minor allele frequency of 0.27 in the global population (based on whole genomes from gnomAD^25^). Strikingly, we observed that this indel correlates with the activity of the entire *AXIN2* VCM (153 kb), with the ALT allele featuring higher H3K27ac, H3K4me3, and H3K4me1 signals, PU.1 and RNA Polymerase II Subunit B (RPB2) binding, and ATAC-seq signal (Fig. 1b, c, d and Supplementary Fig. 1b). We thereby noticed that this effect spreads beyond the predicted VCM region, although with a sharp and clear decline outside the predicted VCM region and with minimal or no impact beyond the Topologically Associating Domain (TAD) boundaries (Fig. 1b and Supplementary Fig. 1b). Further data integration involving extended LCL datasets encompassing H3K27ac, H3K4me1, and H3K4me3 ChIP-seq data from 313 LCLs and RNA-seq from 327 LCLs^12^ as well as ATAC-seq data from 100 LCLs^16^ validated our initial observations. Indeed, as shown in Fig. 1c, the *AXIN2* VCM undergoes a state change from “OFF” (REF allele) to “ON” (ALT allele), and coincides with enrichment of active chromatin marks on the ALT allele and an increase in *AXIN2* expression (Fig. 1d).

Next, we exploited the greater statistical power afforded by the extended datasets to recompute the VCMs across the *AXIN2* locus using various methods (based either on pairwise correlations^10^ or hierarchical clustering (Clomics)^12^) and thresholding strategies (Supplementary Fig. 1c; see Methods section for a detailed description of the analysis). Interestingly, as predicted by the effect size of the genotype (shown in Fig. 1b and Supplementary Fig. 1b), these results support the propagation effect of the indel across the locus, which declines with distance. After merging the VCM components obtained from different datasets, we found that the consensus *AXIN2* VCM can be divided into six regions featuring distinct histone mark enrichment patterns. These regions comprise the promoter, the transcription termination site (TTS), two H3K4me1 satellite regions, and a cluster of five different enhancer units (H3K4me1 and H3K27ac enriched): one located downstream of *AXIN2* (left-enhancer), two intragenic elements (intra1- and intra2-enhancer), a large enhancer overlapping the indel (indel-enhancer) and a far upstream element located ~90 kb upstream of the indel (right-enhancer) (Fig. 1c), as catalogued in Supplementary Table 1. It is thereby worth noting that not all TF and histone mark peaks that are embedded in the *AXIN2* VCM are significantly correlated. However, since all showed differential enrichment according to the genotype (Supplementary Fig. 1b and Supplementary Table 1), we suspect that this observation most likely reflects a statistical power issue. For most of the marks, the regions that showed the largest allele-specific bias were the promoter and the indel-enhancer. These results point to the indel-enhancer as a key regulatory unit within the *AXIN2* VCM. To independently validate this hypothesis, we took advantage of the data generated by Kumasaka et al., 2018^16^, who identified regulatory modules akin to VCMs using a large LCL ATAC-seq dataset (100 individuals) and subsequently defined the CRE hierarchy within each module using a Bayesian approach. Consistent with our findings, this study annotated the ATAC-seq peak that overlapped with the indel as being the “lead” CRE, governing the activity of all other ATAC-seq peaks that are embedded in the *AXIN2* VCM (Fig. 1c) (*AXIN2* VCM ATAC-seq peak coordinates and properties can be found in Supplementary Table 2). Based on these collective analyses, we postulate that the rs143348853 indel controls VCM activity and *AXIN2* expression by modulating the activity of a lead CRE that in turn controls the activity of other CREs within the *AXIN2* VCM.

### A circulating B-cell-specific set of CREs that control *AXIN2* expression

**Fig. 2:**
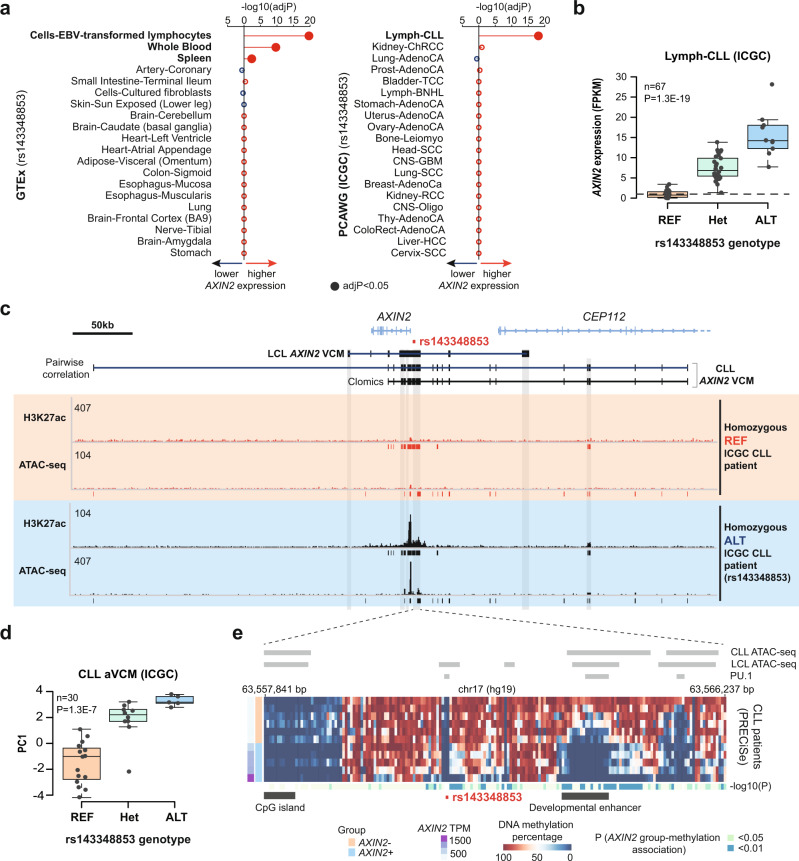
The *AXIN2*-rs143348853 eQTL is highly cell-type-specific. **a** *AXIN2*-rs143348853 eQTL analysis on 49 GTEx tissues (left panel) (only the top 20 tissues based on the nominal *p-*value are shown) and on 20 cancer types from the PCAWG project (right panel) (cancer abbreviations as provided in the original study^26^). The GTEx nominal *p*-values and effect size (normalized effect size, NES) were obtained from the GTEx portal. The PCAWG *p*-values and effect size were obtained from a linear regression model. Significant hits (Bonferroni adjusted *p*-value (adjP) < 0.05) are denoted in bold. **b** *AXIN2* expression from CLL patient lymphocytes from the PCAWG project according to the rs143348853 genotype. **c** Genomic view of the *AXIN2* locus showing the CLL VCM structure based on the two different calling methods) and H3K27ac and ATAC-seq signal profiles from two different CLL cell populations, representing the rs143348853 homozygous REF and ALT genotypes. Meta peaks are displayed below each track. The considered LCL and CLL enhancers, based on high H3K27ac signal, are marked with gray boxes. **d** aVCM measured by the first principal component analysis value (PC1) on all correlated molecular phenotypes (H3K27ac and ATAC-seq) embedded in the CLL *AXIN2* VCM, as called using the pairwise correlation method. **e** DNA methylation on the indel-enhancer from several CLL cells based on WGBS. Cells are grouped based on *AXIN2* mRNA expression (A*XIN2* + and −, which corresponds to high and low *AXIN2* expression, respectively). *AXIN2* expression in transcripts per million (TPM). CLL and LCL ATAC-seq and LCL PU.1 peaks are displayed. CpG island coordinates were obtained from the UCSC Genome Browser. The region found to be in the top 5000 most hypomethylated regions in later stages of B cell development compared to NBCs^29^ (i.e., developmental enhancer) is also denoted. *n* and P indicate the number of considered patients and nominal *p-*value from a linear regression model, respectively. For **b** and **d**, boxes indicate the IQR (25–75%) and the box center indicates the median. Whiskers represent de minimum or maximum values of no further than 1.5 times the IQR for both the top and bottom of the box.

To test the cell type specificity of rs143348853 acting as an *AXIN2* eQTL, we analyzed data from the Genotype-Tissue Expression (GTEx) Consortium as well as from 20 different cancer types that are available in the Pan-Cancer Analysis of Whole Genomes^26^ (PCAWG) cohort, which is part of the International Cancer Genome Consortium (ICGC). Despite the expression of *AXIN2* in many tissues (Supplementary Fig. 2a), we found that rs143348853 is a significant *AXIN2* eQTL only in Epstein Barr Virus (EBV)-transformed lymphocytes, whole blood and spleen (GTEx portal, Fig. 2a), and primary lymph-CLL tumors (PCAWG, Fig. 2a, b). However, we found no significant association between *AXIN2* expression and rs143348853 in B-cell Non-Hodgkin lymphomas (lymph-BNHL) (follicular, marginal, diffuse large B-cell, and Burkitt lymphomas grouped together) (Supplementary Fig. 2b), suggesting that these two types of B cell malignancies feature a distinct regulatory repertoire. Given that LCLs and the ICGC CLL^27^ cells are derived from circulating blood, we define the subset of B cells that show a significant *AXIN2* eQTL as “circulating B cells” to differentiate these from lymph node B cells.

Given that rs143348853 also acted as a *cis*-eQTL in CLL tumors, we next examined to which extent *AXIN2* CREs and the underlying VCM that we detected in LCLs are conserved in CLL. To do so, we mined H3K27ac and ATAC-seq data from 106 CLL patients from the Blueprint project and used again either pairwise correlation^10^ or hierarchical clustering (Clomics^12^) to map the local *AXIN2* VCM. Interestingly, we found that the set of CREs and thus also the VCM configuration in CLL cells are partially different from that of LCLs. Specifically, while we observed several additional, small enhancers, the activation of the principal CREs was restricted to the intra- and indel-enhancers without the implication of the left- and right-(LCL) enhancers (Fig. 2c). Despite this differential configuration, however, VCM activity remained significantly correlated with the rs143348853 genotype (Fig. 2d). In addition, we observed that, based on H3K27ac enrichment data, the indel-enhancer has super-enhancer-like properties that are especially prominent in CLL tumors (Supplementary Fig. 2c). Finally, analysis of H3K27ac data from different classes of healthy donor B cells (naive, memory, plasma, and germinal center B cells) revealed that the set of *AXIN2* CREs in circulating B cells is limited to the intra- and indel-enhancers (i.e., no implication of the left- and/or LCL/CLL right- peripheral enhancers) (Supplementary Fig. 2d), indicating that these enhancers constitute the core *AXIN2*-regulating enhancers.

To acquire high-resolution insights into the engagement level of the indel-enhancer, we mapped local DNA methylation alterations in function of *AXIN2* expression using whole-genome bisulfite sequencing data (WGBS)^28^, which can be mined from the CancerEpiSys-PRECiSe project. In CLL, *AXIN2* expression was accompanied by global DNA methylation loss at regions representing ATAC-seq peaks, which was especially marked around LCL-derived PU.1-binding sites (Fig. 2e and Supplementary Fig. 3a). However, we also observed some DNA methylation loss at these same sites in the absence of *AXIN2* expression, possibly reflecting more spurious chromatin accessibility and TF binding. Intriguingly, based on data from healthy donors, the indel-enhancer was ranked in the top 5000 most hypomethylated regions that undergo epigenetic programming at later stages of B cell development compared to naive B cells (NBCs)^29^. As shown in Supplementary Fig 3b, the indel-enhancer, which is hypermethylated in NBCs and germinal center founder B cells (GCFs) becomes hypomethylated in memory (MBC) and marginal zone (MGZ) B cells, and, surprisingly, does so even in homozygous REF individuals (although to a greater extent in ALT carriers). Moreover, we found that these indel-enhancer-centric differentially methylated regions during B cell development, which are particularly enriched in ATAC-seq peaks, are also associated with rs1433488530 (Pearson’s r = 0.67, Supplementary Fig. 3c and Supplementary Fig. 3d). Together, these findings suggest that during normal B cell maturation, the indel-enhancer becomes predisposed to activation and is increasingly engaged by the regulatory machinery, even though it becomes only fully active in individuals that are deletion carriers.

Our results so far revealed that rs1433488530 is required to fully activate a set of *AXIN2-*controlling CREs in circulating B cells. Intrigued by these results, we set out to provide additional support for rs143348853’s apparent cell type specificity by performing a principal component analysis (PCA) of H3K27ac and H3K4me1 enrichment data derived from the Roadmap Epigenomics^30^ project on the combined LCL and CLL *AXIN2* VCM enhancer regions across all available tissues/cell types. We found that GM12878 LCL (heterozygous for rs143348853) can be clearly distinguished from the other cell types (Supplementary Fig. 4a, b), and shows marked enrichment in left-, indel-, and right-enhancers (Supplementary Fig. 4c, d), with regions spanning the intra-enhancers also active in other tissues. Another B cell sample (fetal cord blood origin, E031) clustered apart from the GM12878 LCL and showed significant enrichment only in the enhancers surrounding the indel (intra2 and indel-enhancers) (Supplementary Fig. 4a and Supplementary Fig. 4d). Furthermore, we observed that, whereas the indel- and right-enhancers appear to possess enhancer properties in other cell types, the left-enhancer is uniquely enriched in LCLs. Analysis of Roadmap DNase data (considering the ATAC-seq regions from LCLs^16^) corroborated these observations (Supplementary Fig. 4a and Supplementary Fig. 4e). In addition, our findings suggest that the two most upstream peaks on the indel-enhancer (peak ids 254432 and 254433) denote putative TF-binding sites that are specific to GM12878, in contrast to the indel-overlapping peak. To rule out the possibility that *AXIN2* expression may bias our results, we analyzed H3K4me3, H3K27ac, and DNase signal at the *AXIN2* promoter and observed no particular enrichment in GM12878 or primary B cells (Supplementary Fig. 4f). Together, our results show that the enhancers observed in LCLs or primary B cells behave in a highly cell-type-specific fashion that is unique to circulating B cells. However, the left- and right-enhancers appear only activated by rs143348853 in EBV-immortalized B cells, suggesting a possible role of EBV in activating distinct enhancers, as has been previously proposed^31^.

Finally, we explored *AXIN2* expression and enhancer activation patterns in two commonly used human CLL lines: OSU-CLL and MEC1 which are heterozygous and homozygous REF, respectively, as verified by Sanger sequencing (Supplementary Fig. 5a). As demonstrated in Supplementary Fig. 5b, we found that *AXIN2* expression levels are consistent with the genotype. Interestingly, H3K27ac enrichment in OSU-CLL (an EBV-immortalized CLL cell^32^) showed an enhancer pattern that is identical to LCLs and not to primary CLL, further supporting the idea that EBV can influence rs143348853-dependent enhancer activation (Supplementary Fig. 5c). Of note, only six CLL patients from the PCAWG cohort appear to carry the EBV infection^33^, and we found that infection status does not impact the *AXIN2*-rs143348853 eQTL (Supplementary Fig. 5d).

### The ALT allele and *AXIN2* expression are associated with a protective role and a better prognosis in CLL patients

**Fig. 3:**
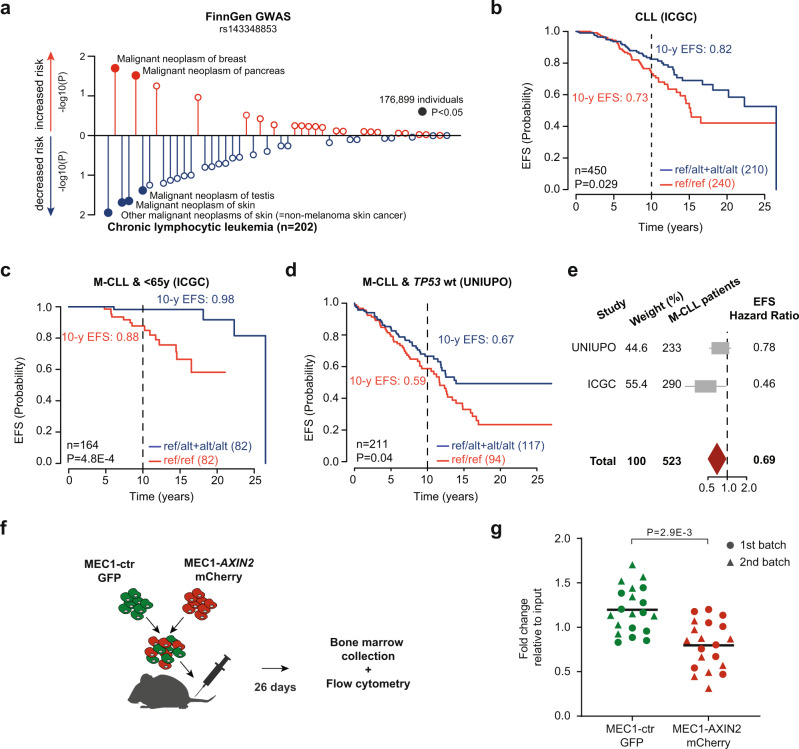
Association between rs143348853 and CLL predisposition and progression. **a** Association between rs143348853 and cancer risk based on information from the Finnish cancer registry (FinnGen GWAS study). **b** Association between rs143348853 deletion carrier status (ref/ref vs. ref/alt + alt/alt) and event-free survival (EFS) probability in CLL. Data was obtained from the ICGC CLLE-ES project^27,35^. *P*-values are based on log-rank tests for the two groups and the probability of 10-year EFS is indicated. **c** Same as **b** but considering the <65-year-old M-CLL patients only. **d** Same as **b** but for the UNIUPO CLL cohort and considering the low-risk (wild-type *TP53*) M-CLL patients only. **e** Meta-analysis of the rs143348853-EFS-hazard ratios for M-CLL patients obtained from a Cox proportional hazards regression model. **f** Experimental design for the in vivo competition assay between MEC1-ctr and MEC1-AXIN2 cells. **g** Fold change of the final cell percentage (day 26) relative to the input percentage (day 0) of MEC1-AXIN2 mCherry + or MEC1-ctr GFP + cells from the in vivo competition experiment; the mean across all 20 mice is displayed, *p-*value calculated with a paired two-sided *t*-test. Source data are provided as a Source Data file. *n* and P indicate the number of considered patients and *p-*value, respectively.

Our analyses so far uncovered a germline indel that appears to control the activity of a large *AXIN2-*associated VCM in circulating B cells. Given its molecular impact and its link to CLL as well as the fact that *AXIN2* has been proposed to function as a tumor-suppressor gene^34^, we hypothesized that this indel may confer differential susceptibility to CLL. To address this hypothesis and discover other phenotypes affected by this indel, we mined the genome-wide association study (GWAS) data from the FinnGen cohort, which comprises 176,899 individuals with genetic information and data from cancer and hospital discharge registries. Interestingly, we found that the ALT allele is among all malignant neoplasms most prominently associated with a reduced risk of CLL (Fig. 3a). In addition, analysis of hospital discharge register data revealed a protective role against lymphoid leukemias and primary lymphoid malignant neoplasms (Supplementary Fig. 6a), suggesting that rs143348853 and *AXIN2* expression might have a broader impact on shaping B cell properties and thus influence cancer development at early stages.

To further characterize the implication of rs143348853 on CLL prognosis, we analyzed 450 CLL patients from the ICGC cohort with clinical outcome data^27,35^. We inferred rs143348853 deletion and non-deletion carrier status from transcriptomes and epigenomes with 91.3% (84/92) prediction accuracy (Supplementary Fig. 6b and Methods) and observed an improved clinical outcome for patients that are rs143348853 deletion carriers (10-year event-free survival (EFS) 82% vs. 73%, *p-*value = 0.029, Fig. 3b). Next, we evaluated the effect of the indel for each CLL subtype. Specifically, there are two CLL classes with distinct prognosis according to the status of immunoglobulin heavy variable (IGHV) genes: patients with IGHV somatic hypermutations (M-CLL) have a markedly better prognosis with a median survival rate of 24 years, in contrast to 10 years for the unmutated or naive status (U-CLL)^36^. We determined that rs143348853 deletion carriers only have a significantly better (i.e. slower) progression if categorized as M-CLL (*p*-value = 0.031 and 0.95, for M-CLL and U-CLL respectively, Supplementary Fig. 6c). Moreover, we observed that rs143348853’s effect is more pronounced if younger patients are selected (<65 years old, lower risk) (*p*-value = 4.8E-4, Fig. 3c and Supplementary Fig. 6d).

To validate the findings from the ICGC cohort, we analyzed an independent CLL cohort of 358 patients from the University of Eastern Piedmont (UNIUPO). rs143348853 genotype information was obtained by PCR (Methods), and its effect on EFS and time to first treatment (TTFT) was assessed. There was no statistically significant difference in EFS when analyzing the entire M-CLL group (Supplementary Fig. 6e). However, when focusing on patients with M-CLL and wild-type in the *TP53* gene, those with at least one ALT allele had a higher EFS compared to homozygous REF patients (10-year EFS: 67% carriers vs. 59% non-carriers, *p*-value = 0.04, Fig. 3d). No statistical significance was found in TTFT (Supplementary Fig. 6f, g). Finally, we considered the effect of rs143348853 in M-CLL patients by performing a meta-analysis using both cohorts, revealing that the ALT allele acted as a protective biomarker with an average EFS-hazard ratio of 0.69 (Fig. 3e and Supplementary Fig. 6h). Overall, these results suggest that the indel rs143348853 and therefore *AXIN2* expression are able to reduce CLL progression in low-risk patients (i.e., M-CLL and *TP53*-wild-type CLL).

To experimentally support the genotype effect in CLL patients, we explored whether leukemic cells with higher *AXIN2* levels proliferate slower, which may contribute to a better prognosis. To do so, we overexpressed *AXIN2* in MEC1 cells (Supplementary Fig. 7a) to study its phenotypic effect on the molecular state of these cells, overall cell proliferation, and CLL progression. RNA-seq analysis revealed that *AXIN2* overexpression in MEC1 cells significantly altered the expression of about 500 genes (Supplementary Fig. 7b, c), with downregulated genes involved in cell division (Supplementary Fig. 7d) or known to be important Wnt pathway activators such as β-catenin, while upregulated genes included Wnt pathway repressors such as GSK3A (Supplementary Fig. 7e), consistent with the Wnt pathway-suppressive function of AXIN2^37,38^. Nevertheless, an in vitro cell proliferation assay did not show any overexpression effect on cell proliferation (Supplementary Fig. 7f). However, given the contextual simplicity of such assay, we decided to perform a competition experiment between the two cell types (*AXIN2* overexpression versus control) in vivo. To do so, we generated GFP-labeled MEC1-control cells as well as mCherry-labeled MEC1-AXIN2 cells after which we systemically distributed 10 million cells as a mixed population (50% GFP+ and 50% mCherry+) in NSG (NOD-*scid* IL2Rgamma^null^) mice via an intravenous tail injection (Fig. 3f). Mice were then sacrificed on day 26 post-injection, which coincided with the first signs of paralysis, reflective of MEC1 cell infiltration into the central nervous system. Thereafter, bone marrow cells were extracted and analyzed by flow cytometry (see Supplementary Fig. 7g for flow cytometry gating strategies). Interestingly, results from two independent biological replicates involving 20 mice revealed that MEC1-ctr GFP+ cells were significantly enriched over MEC1-AXIN2 mCherry+ cells *(p*-value = 2.9E-3, paired two-sided t-test) (Fig. 3g), suggesting that AXIN2 confers a growth disadvantage in this competition assay. In order to eliminate the type of fluorescent protein as a possible cause for the observed effects on MEC1 growth, we performed an additional experiment in which we swapped mCherry and GFP (i.e., MEC1-ctr-mCherry and MEC1-AXIN2-GFP). The results of this experiment were consistent with our original data, validating AXIN2’s capacity to reduce MEC1 proliferation *(p*-value = 1.4E-2, paired two-sided *t*-test) (Supplementary Fig. 7h).

### The ALT-rs143348853 allele creates a de novo MEF2 binding site

**Fig. 4:**
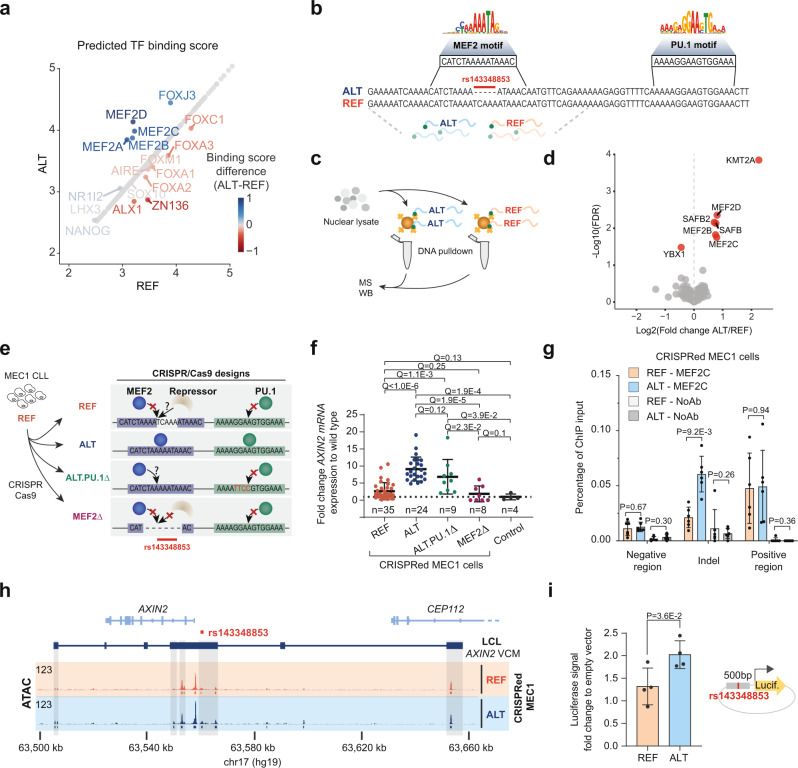
De novo MEF2 TF-binding site formation by rs143348853. **a** Maximum TF-binding site Z-scores for both rs143348853 alleles across 757 bp around the indel. **b** DNA sequence representation of the indel region according to the NCBI dbSNP^23^ build 154 of the two alleles, with a represented logo of MEF2(A) and PU.1-binding motifs based on ENCODE GM12878 data from Factorbook^112^. Underneath, the DNA sequences used for the pulldown experiment are highlighted. **c** Cartoon illustrating the in vitro DNA pulldown experiment with the DNA oligos (see **b**) from the two different alleles, followed by mass spectrometry (MS) or western blotting (WB). **d** Bound TFs to ALT or REF DNA probes detected by in vitro DNA pulldown followed by MS for three replicates. Significant TFs are colored in red and labelled (FDR < 0.05). Only TFs^42^ are shown. **e** Schematic of the CRISPR/Cas9 experiment to modify MEC1 CLL cells to validate the effect of rs143348853 on *AXIN2* expression (ALT and REF) and to test two additional scenarios: ALT.PU.1Δ and MEF2Δ. Control cells are clones that received the plasmid but did not undergo homologous recombination (2 clones for each ALT and REF construct). **f** Each value represents the fold change of *AXIN2* mRNA expression assessed by qPCR relative to wild-type MEC1 cells derived from a single and different clone. *n* indicates the number of clones assessed for each group. Q represents the FDR adjusted *p-*value. **g** MEF2C ChIP-qPCR-based enrichment on distinct regions in the CRISPRed MEC1 cells (*n* = 6 biological replicates). **h** ATAC-seq signal on the *AXIN2* VCM region of CRISPRed MEC1 cells. The track is the combination of three biological replicates (read sum). Below each track, called peaks for each genotype are displayed. **i** Dual reporter luciferase assay performed in MEC1 cells to test the enhancer capacity of a 500 bp sequence centered on the indel for both genotypes (*n* = 4 biological replicates). In **f**, **g**, and **i**, data are presented as the mean ± SD, P represents *p*-values from an unpaired two-sided Welch’s *t*-test, corrected for multiple testing by FDR when necessary (defined as Q), and source data are provided as a Source Data file.

Given rs143348853’s association with CLL (Fig. 3) and the fact that the genotype effect on *AXIN2* expression could be reproduced in clinically relevant lines (Supplementary Fig. 5b), we decided to use the MEC1 line together with LCLs to unravel the mechanistic basis of rs143348853-mediated *AXIN2* VCM formation and expression. First, we set out to determine the regulatory consequence of the rs143348853 5 bp deletion. To do so, we mapped all human TF motifs from HOCOMOCO^39^ on the ATAC-seq peak overlapping the indel (757 bp in total). We then obtained the maximum predicted binding score (represented as Z-scores) for each TF per allele (Fig. 4a) and further narrowed the list of candidates based on expression levels in LCLs (mean FPKM > 0.5 with myocyte-specific enhancer factor 2 C (MEF2C) highest expressed; Supplementary Fig. 8a). Contrary to our expectations, these analyses did not point to PU.1 even though we selected rs143348853 based on the fact that it was a binding QTL for this TF (Fig. 1a). Rather, its motif was detected 25 bp upstream of the indel, suggesting that PU.1 binding is under the control of another, collaborating factor (Fig. 4b). Our analyses revealed several likely candidates with a greater Z-score in ALT vs. REF, namely: MEF2A, MEF2B, MEF2C, and MEF2D, reflecting shared binding motifs among MEF2 family TFs, and FOXJ3. MEF2-type motifs displayed the largest Z-score change between the REF and ALT alleles (mean difference of 0.82 for MEF2 vs. 0.55 for FOXJ3, Fig. 4a). These findings identify MEF2 as the likely causal TF for the de novo activation of the *AXIN2* indel-enhancer, consistent with the documented importance of these TFs in driving enhancer function^40^ and gene regulation^41^. On the other side of the Z-score spectrum, motifs for the TFs ZNF136 and FOXM1 featured a greater Z-score in REF compared to ALT, raising the hypothesis that the activity of the indel-enhancer may also be modulated by potential repressors. To experimentally identify the TFs that differentially bind between the REF and ALT alleles, we performed an in vitro DNA pulldown experiment followed by either mass spectrometry (MS) or western blotting (WB). 39 bp (ALT) and 44 bp (REF) biotinylated DNA probes centered on rs143348853 were each incubated with MEC1 nuclear lysate after which protein complexes bound to the probes were recovered and quantified by MS to compare protein binding affinities between the two probes (Fig. 4b, c). We found 34 significantly differentially enriched proteins, 7 of which were TFs (TF annotation based on Lambert et al., 2018^42^) (Supplementary Data 1). As depicted in Fig. 4d for the MS results, MEF2B, MEF2C and MEF2D were significantly enriched in ALT, as also validated by WB (Supplementary Fig. 8b) (note, MEF2A was enriched in ALT but not significantly). No other candidates from the in silico analysis were detected (ZNF136 or FOXM1) nor emerged as being differentially bound (FOXJ3).

### rs143348853 alone is responsible for *AXIN2* expression and enhancer activation

Our analyses so far demonstrated a direct correlation between indel presence and activity status of the *AXIN2* VCM. To investigate causality, we used CRISPR/Cas9 technology to alter the genotype of MEC1 cells from homozygous REF to homozygous ALT (Fig. 4e). MEC1 cells proved highly refractory to transfection, resulting in poor genome editing efficiencies. To remedy this, we developed a robust CRISPR/Cas9 workflow involving a single plasmid containing the Cas9 protein, the gRNAs, and the template DNA sequence for homologous recombination (Supplementary Fig. 8c and Methods). As CRISPR controls, we also modified MEC1 to the same wild-type genotype and selected clones that received the plasmid but did not undergo homologous recombination (i.e., no locus-specific integration of the mCherry-puromycin resistance cassette). Subsequent gene expression analyses revealed that genetically engineered MEC1 ALT cells (ALT-CRISPR) have higher *AXIN2* expression than their respective MEC1 REF (REF-CRISPR) or wild-type counterparts (Fig. 4f). To explore whether this increased *AXIN2* expression could be linked to MEF2 DNA binding, we performed MEF2C ChIP-qPCR on CRISPRed MEC1 cells, revealing enrichment of this TF on the indel region (Fig. 4g) while ATAC-seq on CRISPRed MEC1 ALT versus REF cells showed increased chromatin accessibility at the LCL *AXIN2* VCM-composing regions (Fig. 4h and Supplementary Fig. 8d) (of note, MEC1 is also an EBV-infected cell line, so the enhancer composition of the *AXIN2* VCM is expected to resemble the one from LCLs). Together, these findings provide additional support for the hypothesized causal relationship between rs143348853 presence and *AXIN2* VCM activation through MEF2 TF binding.

To examine whether increased *AXIN2* expression is indeed driven by enhancer activation, we generated luciferase-based reporter constructs of the REF and ALT alleles, each containing a DNA fragment of 500 bp centered on the indel. As shown in Fig. 4i, the ALT reporter construct exhibited greater luciferase expression than the REF one, although the overall difference was more modest than anticipated.

Next, we aimed to address (1) whether MEF2 is unilaterally controlling the binding behavior of other TFs, thus acting as a pioneer TF^43^; and (2) whether indel-induced *AXIN2* expression could also be explained by a repression model in which the indel would disrupt the binding of REF-bound repressors (e.g., ZNF136 and FOXM1, from Fig. 4a), resulting in enhancer de-repression and *AXIN2* expression. As illustrated in Fig. 4e, to test the former, we genetically engineered the MEC1 cells to generate the ALT genotype while also mutating the PU.1 motif 25 bp upstream of the indel (ALT.PU.1Δ-CRISPR); to test the latter, we deleted all the bases comprising the MEF2 motif (MEF2Δ-CRISPR). Despite several clones showing lower *AXIN2* expression compared to ALT-CRISPR cells, we generally observed maintained expression in ALT.PU.1Δ-CRISPR cells (Fig. 4f). In addition, we observed that MEF2Δ-CRISPR cells lack *AXIN2* expression (Fig. 4f), demonstrating that a gain of MEF2 TF binding and not a loss of TF repressor binding is pivotal to the observed VCM activation. Thus, these results indicate that the MEF2 binding site is necessary for *AXIN2* expression, while the PU.1 one may play a more secondary or cooperative role.

### Massive TF-binding enrichment on the ALT allele

**Fig. 5:**
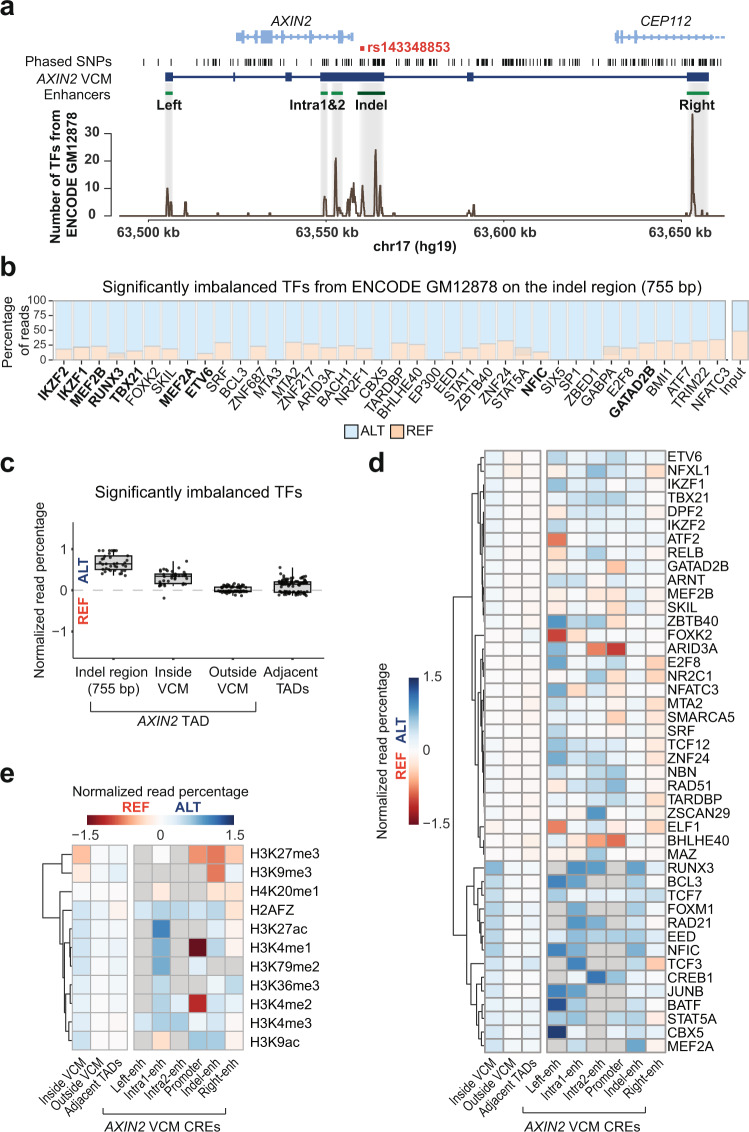
TF-binding preference for the ALT allele. **a** TF-binding density across the *AXIN2* LCL VCM locus from GM12878 ENCODE data. **b** Percentage of reads associated to the ALT or REF-rs143348853 allele for significantly imbalanced TFs on the indel region (ATAC-seq peak of 755 bp overlapping the indel). TFs are sorted from left (low) to right (high) according to the FDR corrected *p*-value from a binomial test. TFs with a detected peak overlapping the region are labelled in bold. The percentage of ChIP-seq input reads is also shown. **c** Percentage of ALT reads from significantly imbalanced TFs for each region of interest: indel (*n* = 38), inside LCL *AXIN2* VCM (*n* = 38), outside LCL *AXIN2* VCM (*n* = 92) but same TAD, and the two adjacent TADs (*n* = 108), where *n* indicates significantly imbalanced TFs. Boxes indicate the IQR (25–75%) and the box center indicates the median. Whiskers represent de minimum or maximum values of no further than 1.5 times the IQR for both the top and bottom of the box. **d** The percentage of ALT reads from significantly imbalanced TFs for each CRE are plotted across particular regions (left panel) as well as the different, principal CREs (right panel) (CREs embedded in the LCL *AXIN2* VCM). The dendrogram clustering based on Euclidean distances was performed only on the overall regions, and the TF list on the right was sorted accordingly. The promoter region was considered as the overlap of H3K4me3 and H3K27ac peaks. Enhancer regions are listed in Supplementary Table 1. To increase statistical power, the right-enhancer region was taken as the full H3K4me1 peak and not only the overlap with the H3K27ac peak. **e** Same as **d** but with all available histone marks. In **c**, **d** and **e**, the percentage of ALT associated reads is displayed as the log2 fold change of ALT read percentage of a TF or histone mark over the ALT read percentage of the input.

The enrichment of MEF2 TFs on the ALT versus REF allele, consistent with our motif-related findings, and the dependence of PU.1 binding on the presence of the indel suggest at first view a model in which formation of a pioneer TF-dependent enhanceosome^44,45^ is required for activation of the indel-enhancer. To address this, we first explored which other TFs may be involved in the activation of the indel-enhancer or more broadly the establishment of the *AXIN2* VCM. To do so, we analyzed ENCODE^46,47^ ChIP-seq data from GM12878 LCL (heterozygous for rs143348853), consisting of 165 different molecular phenotypes (154 TFs and 11 histone marks). Since a phased GM12878 genome is available (i.e., it is known in which chromosome, maternal or paternal, a set of heterozygous variants colocalize), we were able to distinguish the indel effect across the entire *AXIN2* locus, and link ChIP-seq enrichment to either the ALT- or REF-rs143348853 haplotype. As represented in Fig. 5a, regions with the highest TF-binding densities overlapped those encompassed by the *AXIN2* VCM, with more than 20 TFs binding to particular regions. More specifically, based on ChIP-seq peak presence, 12 TFs were detected to bind to the indel region (755 bp) with nine showing a significant ALT binding preference (Fig. 5b, TFs highlighted in bold). However, given the often-arbitrary nature of peak calling, we decided to consider all ENCODE-probed TFs to study the overall impact of the indel on TF allelic binding preferences. Partitioning of TF ChIP-seq reads among the two haplotypes allowed us to identify another 29 biased TFs (thus, a total of 38 out of 154 inspected TFs), all showing a significant preference for the ALT allele (Fig. 5b and Supplementary Data 2). MEF2A and B thereby emerged as top hits, with MEF2C also showing a clear bias toward the ALT allele, albeit only a nominal one as likely caused by the lower number of available reads (Supplementary Data 2). Together, these results provide additional in vivo support for the role of MEF2 in *AXIN2* enhancer/VCM activation. In order to determine potentially important TFs regulating the indel-enhancer, i.e., TFs with a strong DNA motif around the indel, we computed top-site Z-scores for the TFs that intersected with the 38 significantly imbalanced TFs and had available binding scores (PU.1 also included) from randomly accessible enhancers, and plotted the Z-score distribution across the indel-enhancer (Supplementary Fig. 8e). Again, MEF2 TFs appeared to have strong binding sites (only in ALT) compared to a random set of enhancers, together with PU.1, NFIC, and GABPA (both alleles).

The observed ALT-bias was maintained across the entire *AXIN2* VCM, but disappeared outside the VCM region (same TAD) as well as in adjacent TADs (Fig. 5c and Supplementary Fig. 8f). Studying the allelic imbalance for each *AXIN2* CRE independently, we found that most TFs show enriched binding to the ALT allele in most of the CREs, and especially in the left-, two intra- and indel-enhancers (Fig. 5d and Supplementary Fig. 8g). However, we also identified TFs that may act as repressors since they were enriched on the REF allele or TFs that may play dual roles (e.g., ARID3A, GATADB2, and BHLH40) as they were enriched on either the ALT or REF allele depending on the specific CRE. Interestingly, some TFs appeared to have a broad preference towards ALT (e.g., RUNX3, JUNB, or IKZF1), whereas others exhibited a more fine-tuned imbalance on only one CRE (e.g., MEF2, for the indel-enhancer), suggesting TF-specific binding properties (Fig. 5d and Supplementary Data 2). Similar to the binding behavior of TFs, we also observed differential allelic enrichment of specific histone marks: those associated with activation were preferentially enriched on ALT across the entire *AXIN2* VCM, whereas repressive histone marks were enriched on REF with little or no impact outside the VCM regions (Fig. 5e).

### *AXIN2* VCM activation is associated with chromatin compaction

**Fig. 6:**
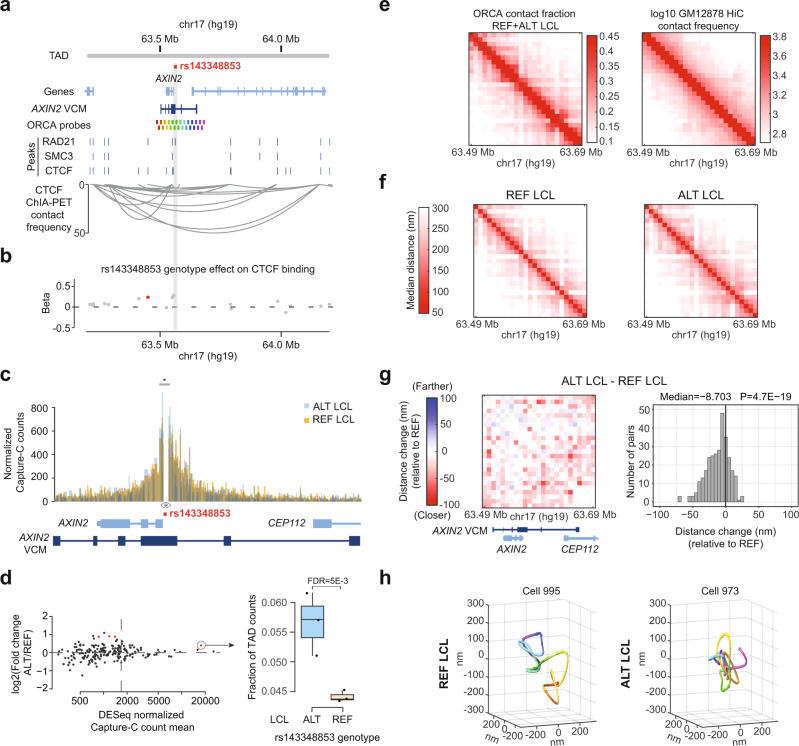
Global chromatin compaction of the *AXIN2* VCM region on the ALT allele. **a** Genomic view of the *AXIN2* locus showing the GM12878 *AXIN2* TAD^72^, the LCL *AXIN2* VCM, the DNA probes used for ORCA, binding sites for RAD21, SMC3, and CTCF in GM12878 from ENCODE, and CTCF ChIA-PET interactions from GM12878^49^. **b** Genetic effect (beta) of the rs143348853 genotype on CTCF binding in LCLs. Significant values (FDR < 0.05) are labelled in red based on FDR corrected *p*-values from a linear regression model. **c** Histogram of the TAD-normalized Capture-C counts from merged replicates for the ALT and REF LCLs. The viewpoint is marked with an eye (indel) and the significant 5 kb bin with differential interaction between the two alleles is marked with a gray box and * (based on DESeq2 results). **d** Log2 fold change of ALT vs. REF normalized and binned (bin-size = 5 kb) Capture-C counts as obtained by DESeq2 (FDR < 0.05 highlighted in red) (the vertical line represents the average count per bin across the *AXIN2* TAD, used as a signal to noise threshold) (left panel). Boxplot of the fraction of TAD counts falling within the 5 kb bin around the viewpoint, containing the *AXIN2* promoter (see **c**, gray box and *) (*n* = 3 biological replicates) (right panel). Boxes indicate the IQR (25–75%) and the box center indicates the median. Whiskers represent de minimum or maximum values of no further than 1.5 times the IQR for both the top and bottom of the box. **e** ORCA contact fraction <150 nm for the merged REF and ALT LCL data (left) and log10 GM12878 HiC contact frequency on the same genomic region (right). **f** Population-median distance in nm for each pair of ORCA segments for REF and ALT LCLs. **g** Matrix (left) and histogram (right) showing the differences between pairs of ORCA segments of population-median distances of REF and ALT alleles. P indicates *p*-value from a two-sided binomial test. **h** Polymer reconstruction of the LCL *AXIN2* VCM 3D architecture obtained by ORCA from one representative LCL single cell for the REF and ALT genotype. The segments are color-coded as illustrated in **a**.

It is well established that enhancer activation can drive chromatin looping upon interaction with other enhancers and promoters^48^. The most intuitive hypothesis based on current concepts is that the induction of *AXIN2* expression would occur through looping of *AXIN2* enhancers and the promoter, as mediated by de novo binding of MEF2 and other TFs. To examine the link between indel-driven *AXIN2* expression and chromatin structure, we performed chromosome conformation analyses. First, we investigated global conformation at the TAD level by analyzing CTCF ChIA-PET data^49^ from GM12878 LCL cells. We found that the *AXIN2* VCM appears to be embedded in a domain that is smaller than those created by most, local CTCF interaction loops (i.e., at a sub-TAD level, resembling a “nanodomain”^14^) (Fig. 6a). We then analyzed CTCF ChIP-seq data from 48 different LCLs^50^ to determine the impact of rs143348853 on each CTCF binding event within the *AXIN2* TAD. Results from this analysis revealed that the ALT allele promotes CTCF binding in discrete peaks that are close to the indel, although the overall effect is small (Fig. 6b). These findings may reflect the ability of CTCF to impose small conformational changes on the ALT allele at the sub-TAD/nanodomain level without compromising the overall TAD structure at the *AXIN2* locus.

Next, we aimed to investigate if the ALT allele induces a conformational change at the *AXIN2* locus. First, we performed NGS Capture-C^51^ on both homozygous ALT and REF LCLs (three independent replicates) taking the rs143348853 as the viewpoint with the specific aim of identifying chromatin regions that interact with the indel-enhancer. Overall, we observed that the Capture-C signal was higher across the TAD for both alleles and that it recapitulated in both alleles the interactions connecting the indel region with the left TAD boundary, seen in the CTCF ChIA-PET, thus validating the experiment and the TAD boundaries (Supplementary Fig. 9a). We next compared how contacts with the indel region are distributed across the TAD for ALT and REF, respectively (Supplementary Fig. 9a). Although we could not observe any striking differences in the overall interaction landscape, we found that the indel (ALT) contacts directly adjacent regions more frequently than in REF, with the latter displaying a more spread-out interaction profile. Statistical analysis confirmed that the 5-kb region surrounding the indel, including the *AXIN2* promoter, was indeed contacted more frequently in ALT compared to REF (DESeq2 FDR = 0.005; Fig. 6c, d). However, none of the other VCM contacts, including the other CREs, met the criteria for statistical significance and had a good signal to noise ratio (higher counts than the TAD contact average, which equals 1750), nor did the promoter region alone (here defined as the region 1 kb upstream of the TSS). Given the focused increase in ALT contacts right around the indel only, we wondered if perhaps an overall, more subtle chromosome conformational change that is not as easily captured in a single bait experiment might explain the VCM activation phenotype.

To address this hypothesis and thus alleviate the limitation of a unique viewpoint while maintaining focus on the *AXIN2* locus, we performed Optical Reconstruction of Chromatin Architecture (ORCA)^52^ on both homozygous ALT and REF LCLs, which allowed us to determine the 3D position of DNA regions of interest at the single-cell level. Specifically, we assessed a region of 200 kb that encompasses the *AXIN2* VCM (illustrated in Fig. 6a) at 8 kb genomic resolution (25 segments). After filtering, we obtained distance matrices for the following number of single cells: 666 (1st replicate REF), 672 (1st replicate ALT), 2281 (2nd replicate REF), and 2390 (2nd replicate ALT). In an effort to increase statistical power and given that the two replicates for each genotype correlated well (0.92 and 0.94 Pearson’s r coefficient for REF and ALT LCLs, respectively) (Supplementary Fig. 9b), we merged the replicates into a single dataset of 2947 and 3062 cells for REF and ALT, respectively. To benchmark our ORCA data to conventional chromosome conformation techniques, we compared it to GM12878 HiC data^53^ (Fig. 6e), demonstrating a good concordance of the *AXIN2* locus conformation between both datasets (Pearson’s r = 0.97) (Supplementary Fig. 9c). Consistent with our Capture-C-based findings, our ORCA data did not reveal any dramatic conformational differences between the REF and ALT alleles (Fig. 6f). We also did not observe significant changes between the *AXIN2* promoter and the indel or between other CREs within the *AXIN2* VCM (Supplementary Fig. 9d). While the former may be explained by the fact that the TSS and the indel are only 3 kb away and may thus not be detected in an 8 kb resolution scheme, the latter appears genuine since the distance between major CREs is well over 8 kb and it is consistent with our Capture-C data. Nevertheless, our ORCA results did point to global compaction of the entire VCM region in the ALT compared to the REF genotype, as indicated by a decrease in the distance across the majority of pairwise contacts in ALT compared to REF (median of ALT minus REF = −8.703 nm; two-sided binomial test *p*-value = 4.7E − 19, Fig. 6g). This observation is consistent when replicates were analyzed independently (Supplementary Fig. 9e) and is visually illustrated by comparing the 3D chromosomal structures within representative, individual cells (Fig. 6h). Together, these findings point to surprisingly small conformational differences between the ALT and REF alleles with a focused increase in ALT contacts right around the indel and subtle, long-range chromatin compaction as the principal alterations.

## Discussion

Gene expression is governed by complex, often locus-dependent regulatory mechanisms^13,54^, making it difficult to distill generalizable and interpretable rules that can aid in detangling the contribution of non-coding variants to traits or disease^3^. Nevertheless, emergent concepts are converging on the notion that gene regulation acts through subnuclear compartments, which afford a high degree of regulatory coordination. A key challenge now is to identify genomic loci that allow us to dissect how these compartments, statistically referred to as VCMs^10^ or CRDs^12^, or experimentally defined as regulatory microenvironments^13^ or chromatin nanodomains (CNDs)^14^, assemble. In this study, we present one such locus, *AXIN2*, featuring a VCM whose activity, we found, is modulated by a single, germline, non-coding 5 bp indel: rs143348853. We determined that this indel features high expression specificity (eQTL) for circulating B cells (LCLs and CLLs), providing a unique opportunity to dissect the molecular mechanisms underlying VCM formation, transcriptional compartmentalization, and its contribution to disease-relevant phenotypes.

**Fig. 7:**
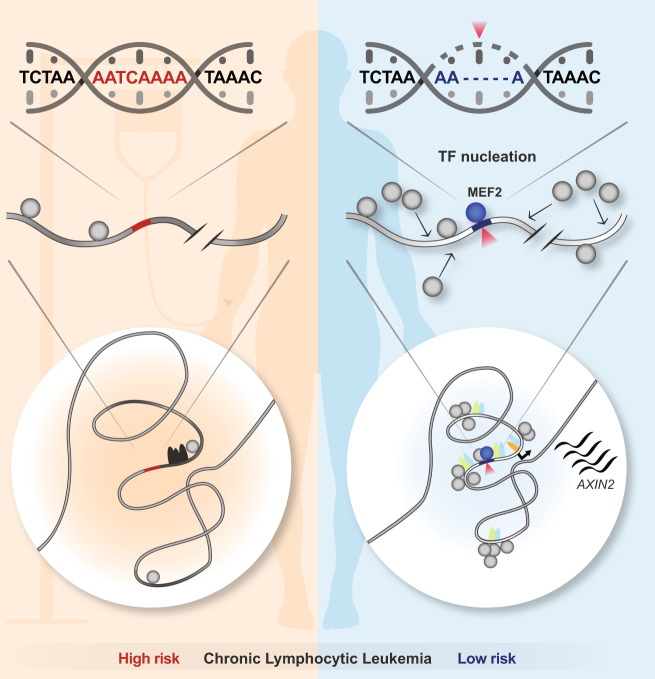
Graphical summary. Our data support a model in which a non-coding, germline 5 bp deletion activates a set of long-range enhancers by creating a de novo MEF2 TF motif, which in turn triggers *AXIN2* expression. MEF2 presence correlates with long-range TF nucleation and chromatin compaction, suggesting that these phenomena are major drivers of *AXIN2* VCM formation. In addition, this 5 bp deletion and *AXIN2* expression are associated with reduced CLL predisposition and disease progression.

As summarized in Fig. 7, our analyses revealed that the variant creates a single de novo binding site for a MEF2 TF. The latter then acts to induce *AXIN2* expression by serving as a nucleation trigger, resulting in a dramatic switch from a repressed to an active transcriptional environment, not only locally, but over a region that spans >150 kb. Thus, our findings indicate that the variant initiates a process that results in a unique regulatory environment, which is believed to be the root of transcriptional hub assemblies^55^. Both our Capture-C and ORCA results suggest that this process is not driven by striking chromosome conformational changes, as we only observed a focused increase in ALT contacts right around the indel and subtle compaction of the entire VCM region in the ALT compared to the REF configuration. That at least some enhancers may regulate target gene expression without requiring these enhancers to ever come into close contact with their target promoters has already been documented^48^. Under this scenario, TF binding to exposed sites within these enhancers may lead to a sufficient increase in the local concentration of compatible TFs such that these compatible TFs demix from the nucleoplasm and form a large, multivalent condensate on the chromatin^56–59^. Indeed, condensates of transcription-associated factors such as BRD4, Mediator, and PolII spanning several hundred nanometers have been observed at some super-enhancer loci^58^. These condensates may mediate long-range communication without the need for nanoscale proximity, and act to further concentrate activating factors while excluding non-specific genomic elements^60^. This is consistent with the observed condensation of a large panel of TFs onto the ALT allele over a relatively large (~100 kb) genomic region, which in turn has significant effects on *AXIN2* expression. Together, these findings support a model in which the formation of the *AXIN2* VCM relies on the coordinated action of multiple TFs, while also depending on a specific nucleation event that triggers the establishment of the underlying, regulatory microenvironment.

Our mechanistic dissection of the *AXIN2* VCM not only allowed us to tackle fundamental questions of how gene activation and VCM formation are controlled, but it also allowed us to provide insights into the flow of molecular information from non-coding variant to likely phenotype (in this case, CLL susceptibility and disease progression, which have already been suggested to also be influenced by regulatory variation^61^). That this is a great challenge in the field is illustrated by the fact that only a handful of studies have so far managed to molecularly connect small, regulatory variants to complex (non-Mendelian) traits (reviewed in ref. ^62^). Moreover, even fewer studies have considered the role of chromosome conformation in this process, mainly implicating variant-mediated enhancer-promoter looping alterations^7,8,63^, which seemingly contrasts with the variant effects observed in this study. Our motivation to specifically investigate the impact of rs143348853 on CLL disease susceptibility and/or progression was driven by its unique impact on *AXIN2* expression in CLL cells and the latter gene’s property as a tumor-suppressor^34^. Our analyses suggest that the variant and thus *AXIN2* up-regulation could serve as a prognostic marker since it is associated with a better outlook for 10-year event-free survival of CLL patients, especially low-risk and relatively young (<65 year old) CLL patients, consistent with our in vivo data demonstrating AXIN2’s ability to reduce in vivo CLL cell proliferation in an overexpression context. In addition, data from the FinnGen population study revealed that the deletion may have protective properties against developing CLL and other lymphoid neoplasms. However, additional studies will be required to fully understand the interplay between *AXIN2* expression and the distinct, cellular phenotypes observed in CLL, and to determine its diagnostic and therapeutic value.

In conclusion, by experimentally dissecting the statistical VCM concept, we were able to identify a germline variant with striking gene regulatory properties. Given the endogenous nature of this variant and its phenotypic impact, we envision that the *AXIN2* locus may become a valuable model system for uncovering additional insights into the molecular mechanisms that drive VCM and regulatory hierarchy formation, including the importance of TF-binding site identity, strength, density and cooperativity in these processes.

## Methods

### Screening of genetic variants that are associated with VCM activity

We used the LCL dataset from Waszak et al., 2015^10^ which comprises 47 individuals from the 1000 Genomes Project^64^ (47 for all ChIP-seq assays and 46 for RNA-seq). Meta peaks (BED files), normalized (by library size, covariates regressed out, and qqnorm transformed), and raw read counts for H3K27ac, H3K4me3, H3K4me1, PU.1, and RPB2 ChIP-seq assays and RNA-seq, QTL analyses results for all molecular phenotypes and genotype information were obtained directly from the authors of Waszak et al., 2015^10^. Screening QTLs was performed using their calculated Q-value or FDR (10% cutoff). Alignment files were obtained from the ArrayExpress Archive: E-MTAB-3657 (ChIP-seq data) and E-MTAB-3656 (RNA-seq data). ChIP-seq BAM files were subjected to duplicate removal using the MarkDuplicates function from Picard v22.2.9 (http://broadinstitute.github.io/picard/). BigWig files were generated with bamCoverage^65^ v3.5.0 (10-bp bin size and RPKM normalized; with --extendReads flag when paired-end). For the PU.1-binding boxplot (Fig. 1d), raw counts were normalized with the rpkm function (edgeR^66^ v3.28.1 in R) (no covariate regression was performed).

### Extended LCL datasets

For the Delaneau et al., 2019^12^ dataset (313 LCLs for ChIP-seq and 327 LCLs for RNA-seq), we obtained access and downloaded part of the H3K27ac, H3K4me1, and H3K4me3 ChIP-seq and RNA-seq alignment files from the Gencord cohort (EGAS00001003485), and the other part was downloaded from the publicly accessible ArrayExpress Archive (E-MTAB-3657). Genotype and covariate information was obtained directly from the authors. ChIP-seq BAM files were subjected to duplicate removal using Picard v22.2.9. Read count matrices were produced with HOMER^67^ v4.11 (without normalization) using the meta peaks from Waszak et al., 2015^10^ for the ChIP-seq assays or htseq-count^68^ v0.12.4 (-s no -m union) using exons for the RNA-seq (gtf file downloaded from Ensembl, GRCh37 release 75). For paired-end ChIP-seq samples, read counts were multiplied by two (given that HOMER counts fragments as half for paired-end data), normalized with the DESeq2^69^ v1.26.0 R package counts function, covariates regressed out and transformed to a normal distribution per individual with qqnorm in R. For the RNA-seq boxplot (Fig. 1d), raw read counts were normalized by rpkm (edgeR^66^ v3.28.1) (no covariate regression was performed).

For the Kumasaka et al., 2018^16^ ATAC-seq set (100 LCLs), raw alignment data was obtained from the European Nucleotide Archive (ERP110508 [https://www.ebi.ac.uk/ena/browser/view/PRJEB28318]) and processed data (peaks, genotype information and normalized/raw read counts) from the original publication (Zenodo: https://zenodo.org/record/1405945#.X7PXCMco-uM). For the ATAC-seq boxplot (Fig. 1d), raw read counts were normalized by rpkm (edgeR^66^ v3.28.1) (no covariate regression was performed).

BigWig files were generated with bamCoverage^65^ v3.5.0 (10-bp bin size and RPKM normalized; with --extendReads flag when paired-end).

### VCM calling and effect size in LCLs

The VCM calling was performed on normalized, covariates regressed out and qqnorm transformed count matrices (peaks x samples) using two distinct methods: (1) the original VCM calling method^10^: we calculated the Pearson’s correlation matrix between peaks and grouped all correlating peaks in modules that passed the FDR threshold of 0.1%. And 2) Clomics v1.0, another method to map VCMs, also named *cis*-regulatory domains (CRDs) in Delaneau et al., 2019:^12^ from the same correlation matrix, hierarchical clustering is applied that further delineates the modules (instead of a fixed FDR thresholding).

For the Waszak et al., 2015^10^ dataset, we remapped the VCMs using the original VCM calling method (0.1% FDR). For the Delaneau et al., 2019^12^ dataset, we computed the VCMs with meta peaks from Waszak et al., 2015^10^ using all three histone marks (two individuals lack one of the three marks, so 311 LCLs in total). VCMs (CRDs) obtained from Clomics and the original VCM calling method (0.1% FDR) were very similar, but resulted in a very large VCM at the *AXIN2* locus, spanning multiple TADs (Supplementary Fig. 1c). We concluded that given the high number of individuals, using a 0.1% FDR threshold for the pairwise correlations may have been too lenient, since this value corresponded approximately to a correlation of *r* ≈ 0.24. Therefore, we decided to use more stringent *r* correlation thresholds (*r* > 0.3, 0.4, 0.5 and 0.6). Given the similarity of the *AXIN2* VCM mapped from other datasets (Waszak et al., 2015^10^ and Kumasaka et al., 2018^16^), we concluded that a pairwise correlation with an *r* > 0.5 threshold was the most optimal approach. For the Kumasaka et al., 2018^16^ ATAC-seq dataset, we called VCMs using the provided log2 FPKM matrix with the original method (0.1% FDR), r > 0.5 and Clomics, and obtained similar results. The output of all these methods regarding *AXIN2* VCM composition in LCLs can be observed in Supplementary Fig. 1c.

The activity score of VCMs (aVCM) was calculated as described in Waszak et al., 2015^10^. The effect size of the genotype (beta, Fig. 1b, Supplementary Fig. 1b and Supplementary Table 1) on all studied molecular phenotypes was calculated using the normalized, covariates regressed out, and qqnorm transformed read counts with a linear regression (lm in R) and corrected for multiple testing by FDR (only associations between rs143348853 and peaks overlapping the *AXIN2* TAD and adjacent TADs were tested).

### Blueprint consortium data

We applied for access to the European Genome-Phenome Archive (EGA) dataset: EGAD00001004046, generated by the Blueprint Consortium, which encompasses H3K27ac ChIP-seq and ATAC-seq data from 106 CLL patients. In addition to CLL, we also retrieved H3K27ac data from 13 healthy donor primary B cells from the same dataset. The Blueprint project was funded by the European Union’s Seventh Framework Programme (FP7/2007–2013) (grant agreement 282510), more information and a full list of investigators who contributed to the generation of the data is available from www.blueprint-epigenome.eu. H3K27ac ChIP-seq and ATAC-seq fastq files were downloaded, replicates were merged, aligned with bwa mem v0.7.17^70^ to hg19, converted to the BAM format, and sorted using SAMtools^71^ v1.9. Read duplicates were removed with Picard v22.2.9. BigWig files were generated as done for LCLs. Normalized count matrices, VCMs, and aVCMs were obtained with the same pipeline as for the Delaneau et al., 2019^12^ ChIP-seq data, yet using the peak coordinates determined by Beekman et al., 2018^72^ and without covariate regression. CLL VCMs were mapped using H3K27ac and ATAC-seq signals together from the 106 CLL patients. However, only 30 had available genotype information in the PCAWG project to represent aVCM in the function of the genotype.

### Enhancer conservation across Roadmap cell types

Consolidated aligned read files (tagAlign) from the Roadmap Epigenomics project^30^ for H3K27ac, H3K4me1, H3K4me3 ChIP-seq, and DNase from different tissues or cell types (98, 127, and 53, respectively) were downloaded. Note that the Roadmap Epigenomics website provides processed datasets for 111 different cell types plus 16 from ENCODE. The number of tags overlapping the different regions of interest was counted using the countOverlaps function from the Bioconductor R package GenomicRanges^73^ v1.38.0 and normalized by library size. To obtain the tag density across the *AXIN2* locus, the whole segment was divided into bins of 36 bp (same length as the tags). *AXIN2* enhancer regions were considered as the overlap of H3K27ac and H3K4me1 LCL peaks (listed in Supplementary Table 1), the promoter region as the H3K4me3 LCL peak and ATAC-seq peaks from Kumasaka et al., 2018^16^ for the DNase regions. In addition, the CLL enhancer located in chr17:63706619–63709184 (hg19) (based on a high H3K27ac signal) was also added to the set of enhancers. H3K4me1 and H3K27ac PCA outliers were identified using the local outlier factor (LOF) function from the bigutilsr v0.3.4 R package. Tag enrichment on specific regions was assessed using the pnorm function in R (considered as one-sided *p*-value) and corrected by FDR when multiple regions of the same mark were assessed.

### TF motif analysis

For the comparison of TF motif scores between the two *AXIN2* enhancer genotypes (ALT and REF), TF-binding models were first downloaded from the HOCOMOCO database^39^ (PWM; mononucleotide models; human; v11) and then scored across the enhancer sequence, spanning the rs143348853 indel. Enhancer boundaries were defined based on accessibility data provided in Kumasaka et al., 2018^16^ (comprising a total of 757 bp around the indel). For comparison across TFs, PWM scores (log scale) were first transformed into Z-scores by using a per-TF PWM score distribution derived from sampling accessible genomic regions (>5000) of the same dataset^16^ and scoring each TF model across. TF-binding site Z-scores were computed for both ALT and REF (rs143348853) *AXIN2* enhancer sequence and only the highest Z-score was retained for each genotype (ALT and REF) and TF for the final comparison.

To obtain the binding profile of significantly imbalanced TFs, we computed a top-site Z-score for the TFs that intersected with the imbalanced TF list (indel region) and had available binding scores (PU1/SPI1 also included). The Z-scores were computed as follows: 5000 random enhancers from the ATAC-seq LCL data^16^, extended around the peak center to 700 bp total length, were scored with the TF motifs and for each enhancer and each TF the top-site score was extracted. This distribution of 5000 top-scores for each TF was then used to compute a Z-score for the *AXIN2* enhancer for both REF and ALT genotypes.

### TF density and allelic imbalance from ENCODE GM12878 ChIP-seq data

We downloaded the raw*.fastq* files from 140 single-end and 62 paired-end ChIP-seq experiments from the ENCODE Project^46,47^ (experiments with released status only), representing 154 different TFs and 11 histone marks for the GM12878 LCL. We also downloaded the 26 associated control experiments (ChIP-seq input, 23 single-end, and 3 paired-end). Fastq files from replicated assays were merged except if they were paired- and single-end, then they were processed independently. In addition, we downloaded released BED tracks for the available TFs (145), choosing “Optimal IDR”, “pseudoreplicated” and “IDR thresholded peaks” output only. BED files from hg38 were lifted to hg19 with rtracklayer^74^ 1.46.0 in R and replicates were merged with Bedtools^75^ v2.27.1. See Supplementary Data 3 for the ENCODE metadata used for this analysis. We collected GM12878 phased genotype data from the 1000 Genomes Project compiled by the Genome In a Bottle consortium^76^, (ftp-trace.ncbi.nlm.nih.gov/giab/ftp/release/NA12878_HG001/latest/GRCh37/). *Creation of phased diploid genomes for GM12878*. Using the phased genotype*.vcf* file for GM12878 and the hg19 human reference genome*.fasta* file downloaded from Ensembl (GRCh37 release 75), we used the vcf2diploid^77^ tool (https://github.com/abyzovlab/vcf2diploid) to create two personalized haplotype sequences (maternal and paternal*.fasta* files). This step allowed us to precisely quantify the reads mapping to each sequence, especially in the case of indels, when the mapping can fail if performed uniquely on the reference genome, thus introducing bias in estimating the allelic imbalance. vcf2diploid tool also generates*.chain* files which allowed us to generate two*.vcf* files, one for each personalized diploid genome. Of note, no tool allowed us to perfectly perform this step, so we used homemade scripts. *ChIP-seq data pre-processing*. Single-end and paired-end reads were aligned with bwa mem v0.7.17^70^ to the two personalized genomes that were created in the previous step. The output was converted to the BAM format and sorted using SAMtools^71^ v1.9. No read duplicate removal was performed. Then we used freebayes^78^ (v.1.3.4) to re-genotype the 53941 heterozygous variants on chromosome 17, independently in the paternal and maternal*.bam* files. Freebayes reports the reference and alternate allele counts for each variant. *Data analysis*. For each specific region, TFs, histone marks, and input read counts from all heterozygous variants overlapping the region were summed and we calculated the *p*-value using a two-sided binomial test (correcting the probability of success, here ALT reads, according to the ALT read percentage from the input). If the sum of total reads was lower than 6, the TF or histone mark was discarded from the analysis. For each region, the list of *p*-values was corrected by FDR. For plotting purposes (Fig. 5 and Supplementary Fig. 8), the fold change of the ALT percentage of a TF or histone mark over the ALT percentage of input was calculated and log2 transformed. To avoid log2(0), one read for both alleles was given to the TF. Of note, we acknowledge that this analysis is highly dependent on having phased variants that overlap the region of interest, therefore small regions suffer from a statistical power issue. The results shown in Fig. 5c, d, e and Supplementary Fig. 8f, g can be found in Supplementary Data 2. To represent TF-binding density, a genomic window comprising the *AXIN2* VCM was divided in bins of 10 bp and the number of peaks for all TFs overlapping each bin was counted using the countOverlaps function from the Bioconductor R package GenomicRanges^73^ v1.38.0.

### ctcfQTL analysis

To analyze the effect of the indel on CTCF binding, we mined Ding et al., 2014^50^ which features CTCF ChIP-seq data from 51 different LCLs. Genotypes were obtained from the phase1 and phase3 1000 Genomes Project (only 48 had rs143348853 genotype information). BAM files were downloaded from the European Nucleotide Archive (ERP002168 [https://www.ebi.ac.uk/ena/browser/view/PRJEB1350]) and replicated from the same LCL were merged. Read duplicates were removed with Picard v22.2.9. Normalized count matrices were obtained with the same pipeline as for Delaneau et al., 2019^12^ ChIP-seq data, yet using the CTCF ChIP-seq peak coordinates obtained from ENCODE GM12878 and without covariate regression. The effect size of the genotype (beta, Fig. 6b) was calculated with a linear regression (lm in R) and corrected for multiple testing by FDR (only associations between rs143348853 and peaks overlapping the *AXIN2* TAD were tested).

### Super-enhancer analysis

H3K27ac ChIP-seq BAM files from homozygous ALT-rs143348853 individuals, 4 LCLs (GM06986, GM11931, GM12275, GM12287), and 4 Blueprint CLL patients, were filtered to remove duplicate reads with Picard v22.2.9 and ENCODE blacklisted regions with Bedtools^75^ v2.27.1. Super-enhancers and enhancer ranking were assessed for each individual with the Rank Ordering of Super-Enhancers (ROSE2)^79^ algorithm (-s 12500 -t 1500) using the respective LCL or CLL H3K27ac peak coordinates. The percentage of H3K27ac signal and the rank for each enhancer from the ROSE2 AllEnhancers.table.txt file was averaged across all 4 individuals of the same cell type. Enhancers were considered super if in at least 1 of the 4 individuals it was detected as such by ROSE2.

### Pan-cancer *cis*-eQTL analyses

We applied for access to ICGC/TCGA Pan-Cancer Analysis of Whole Genomes Project data^26^ and obtained germline variant calls and donor-matched tumor gene expression datasets for cancer *cis*-eQTL analyses (approved access to ICGC data under DACO-1088517) (TCGA dbGaP Study Accession phs000178.v11.p8). We performed linear regression analysis based on rank inverse normal transformed *AXIN2* expression levels (FPKM UQ) and germline rs143348853 genotypes (genotype quality ≥ 20) across 20 cancer types (10–96 donors with European genetic ancestry). We accounted for population structure based on three principal components that were derived from genome-wide SNPs^26^ and we adjusted for multiple testing using Bonferroni correction.

### Whole-genome bisulfite sequencing data

Primary WGBS and RNA-sequencing data (EGAD00001005970) for CLL^28^ and tagmentation-based WGBS data from healthy donor B cells (EGAS00001000534)^29^, which are part of the CancerEpiSys-PRECiSe project, were aligned and preprocessed by the bisulfite alignment and quality control workflow of the DKFZ Omics IT and Data Management Core Facility (https://github.com/DKFZ-ODCF/AlignmentAndQCWorkflows) using the UCSC hg19 human genome assembly. CpG-level methylation calls were imported and further analyzed in R using methrix^80^ v1.4.07. CpG sites embedded in the indel-enhancer with minimum five reads in 80% samples were tested for association analysis between CpG methylation levels (qqnorm transformed across regions) and *AXIN2* gene expression (CLL), rs143348853 genotype status (normal B cells) and cell type (normal B cells) with a linear regression model. Germline genotypes for rs143348853 in normal B cells were called using freebayes^78^ using default parameters. Statistical models for normal B cells included information about cell types (e.g. naïve B cells, memory B cells) and rs143348853 genotype status (ref/ref, ref/alt, alt/alt).

### CLL survival analysis (ICGC data)

We obtained information about clinical outcomes for 450 CLL patients from the ICGC Data Portal (https://dcc.icgc.org/releases/current/Projects/CLLE-ES). Event-free survival (EFS) was defined as the time from diagnosis to the following events: progression, relapse, or death due to any cause. The median follow-up time was 7.0 years. Germline rs143348853 genotype information, CLL epigenome subgroup (n-CLL, i-CLL, m-CLL), and *AXIN2* gene expression (Affymetrix U219 array) were available for 92 donors. This sub-cohort was used to develop a logistic regression model that infers rs143348853 deletion carrier vs. non-carrier status (rs143348853 carrier status ~epigenome subgroup + *AXIN2* expression) and achieves a prediction accuracy of 91.3% (84/92 individuals) using WGS-derived rs143348853 genotypes. This predictive model was used to derive rs143348853 deletion carrier status (Phred quality score  >  10) for the remaining set of 358 patients. The cohort has the following proportions for type (390 CLL, 44 monoclonal B lymphocytosis (MBL), 16 small lymphocytic lymphoma (SLL)), IGHV status (290 M-CLL, 154 U-CLL), and Binet stage (401 A, 36 B, 10 C). Survival analysis was based on the Kaplan–Meier estimator, log-rank tests, and the Cox proportional hazards regression model using the R package survival (v2.44).

### CLL survival analysis (UNIUPO data)

The cohort included consecutive CLL patients followed at the University of Eastern Piedmont. DNA samples were extracted from fresh or frozen PBMCs isolated by Ficoll-Paque gradient centrifugation or from granulocytes. Patients provided informed consent in accordance with local institutional review board requirements (Comitato Etico Interaziendale di Novara, Italy) and the Declaration of Helsinki (study number CE 8/11 and CE 120/19). The study was approved by our local Ethics Committee: Comitato Etico Interaziendale di Novara, Italy (study number CE 8/11 and CE 120/19). Patients did not receive any type of compensation. *TP53* mutations were analyzed by Sanger sequencing in exons 2–11 and mutations were reported if present in the IARC database. To test the IGHV-D-J rearrangement, the DNA of each patient was amplified by PCR and subsequently subjected to Sanger sequencing. FASTA sequences were analyzed using the international ImMunoGeneTics information (IMGT) system (http://www.imgt.org). Classification into mutated IGHV genes and unmutated IGHV genes was based on the established 98% cutoff value for identity to the germline sequence (<98% for mutated and ≥98% for unmutated IGHV genes). FISH karyotype was performed on peripheral blood mononuclear cells using the XL DLEU/TP53 probe for del17p detection (Cytocell Acquarius, Cambridge, England). rs143348853 genotyping was performed using a PCR as described in ref. ^81^. Briefly, a pair of primers (Supplementary Table 3) was used to amplify the indel region (35 cycles), half of the product was mixed with an equal amount of pre-amplified DNA from MEC1 cells and run for an additional cycle (the other unmixed half was also run another cycle). The final products were then run on a 4% agarose gel for 1 h 30 min. A donor was considered: homozygous REF if both samples (mixed with MEC1 and unmixed) had one band, heterozygous if both samples had two bands, and homozygous ALT if the mixed sample had two bands but the unmixed sample had one band. Samples with bands of the wrong size or with more than two bands were discarded. Finally, the cohort yielded 358 genotyped patients (152 REF, 164 Het, 42 ALT) with the following proportions for gender (157 female, 201 male), age (120 < 65 y, 238 > 65 y), type (273 CLL, 84 MBL, 1 SLL), IGHV status (233 M-CLL, 116 U-CLL), Binet stage (288 A, 42 B, 28 C) and *TP53* status (318 wt, 33 mutated). Survival analysis was based on the Kaplan–Meier estimator, log-rank tests, and the Cox proportional hazards regression model using the R package survival (v2.44). The meta-analysis was performed using the R package metafor^82^ (v3.0–2).

### Association between rs143348853 and cancer risk in the Finnish population

We obtained GWAS data summary statistics for rs143348853 and information from the cancer registry (phenotype code C3) and hospital discharge register (phenotype code CD2) for 176,899 individuals from the FinnGen study cohort (release 4; http://r4.finngen.fi/variant/17-65564173-AAAATC-A).

### Cells and cell culture

The following LCLs (obtained from the Coriell Institute) were used to represent the three different genotypes for rs143348853: GM12878 (heterozygous), GM12282 (homozygous REF), and GM11931 (homozygous ALT). OSU-CLL was acquired from The Ohio State University’s Human Genetics Sample Bank. The MEC1 cell line was purchased from DSMZ (ACC 497). HEK293 cells were used for lentivirus production. LCLs and OSU-CLL were cultured using RPMI 1640 GlutaMAX HEPES (Gibco), MEC1 cells with IMDM GlutaMAX HEPES (Gibco), and HEK293 cells with DMEM 4.5 g/L D-Glucose, L-Glutamine Pyruvate (Gibco), all supplemented with 10% fetal bovine serum (FBS, Gibco) (15% FBS for LCLs) and 1% penicillin/streptomycin (P/S, Gibco).

### Genomic DNA extraction and rs143348853 genotyping

Genomic DNA was obtained using the Quick-DNA miniprep kit (Zymo Research) and the genotype was verified by Sanger sequencing (Microsynth) using the Axin2gt primers (Supplementary Table 3) PCR product. The analysis and alignment were performed using SnapGene v4.2.11.

### RNA extraction and *AXIN2* qPCR

Cells were harvested, washed with PBS, and RNA was obtained using either the Direct-zol RNA Miniprep kit (R2052, Zymo Research) or guanidinium thiocyanate-phenol-chloroform extraction protocol. cDNA was synthesized using 1–5 µg of RNA, SuperScript II Reverse Transcriptase (Thermo Scientific), and Anchored Oligo(dT)_20_ VN primers (E0106-01, EURx) in a 20 µl total reaction volume. cDNA was diluted 4 times and 2 µl were used to run the qPCR on a StepOnePlus or QuantStudio 6 Flex Real-Time PCR System (Applied Biosystems) using the PowerUp SYBR Green Master Mix (Thermo Scientific) and MicroAmp Fast Optical 96-Well Reaction Plates (Thermo Scientific), in three technical replicates. HPRT1 was used as a housekeeping gene. Primer sequences are listed in Supplementary Table 3.

### Protein extraction and western blotting

#### Protein lysate extraction

Cells were washed with PBS and incubated in lysis buffer (25 mM Tris pH 8, 50 mM NaCl, 0.5% Na Deoxycholate, 0.1% SDS, 0.5% NP40, 2.5 mM EDTA, 0.5 mM PMSF, 2.5 mM NaF and 1× Pierce Protease Inhibitor (EDTA-free, Thermo Scientific)) for 30 min at 4 °C with constant agitation, followed by sonication at 1 s on/off intervals for 45 s at low intensity on a Bioruptor Plus sonication device (B01020001, Diagenode). The supernatant containing total protein lysate was obtained after 10 min centrifugation at 16,000 rcf at 4 °C. The protein concentration was assessed using the Quick Start Bradford Protein Assay kit (Bio-Rad). *Western blotting*. Protein lysates were analyzed by western blotting under the following conditions: 25 µg of protein lysate/lane was loaded into 10% Mini-PROTEAN TGX Precast gels (#4561034, Bio-Rad), run for 30 min at 50 V, and then at 150 V until the 25 kDa size band reached the bottom of the gel, a wet transfer was performed at 4 °C during 1 h at 100 V/250 mA with the transfer buffer containing 20% EtOH using a PVDF membrane. After transfer, the membrane was blocked 1 h at RT with 5% Blotting-Grade Blocker (#1706404, Bio-Rad) in TBST followed by overnight incubation with primary antibody at 4 °C. The membrane was washed 3 × 5 min with TBST and incubated 1 h at RT with a secondary antibody. The membrane was washed again 3 × 5 min with TBST and revealed with Immobilon Western Chemiluminescent HRP Substrate (Merck Millipore) using the ChemiDoc XRS + scanner (Bio-Rad). The antibodies and dilutions used were: AXIN2 1:1000 (#2151, Cell Signaling), TBP 1:5000 (C15200002, Diagenode), MEF2 1:500 (sc-313, Santa Cruz), anti-rabbit HRP 1:5000 (sc2004, Santa Cruz) and anti-mouse HRP 1:1000 (#115-035-146, Jackson ImmunoResearch). The Precision Plus Protein Dual Color Standards (Bio-Rad) was used as a molecular weight marker.

### Lentiviral infection

The lentiviral plasmid of interest was co-transfected in HEK293 cells with pCMVR8.74 (#22036, Addgene) and pMD2.G (#12259, Addgene) in a 2:1:1 ratio, respectively, using the CalPhos Mammalian Transfection Kit (Takara Bio) or FuGENE 6 Transfection Reagent (Promega). The following day the medium was changed and 2 days later the medium containing the lentiviruses was collected, filtered through a 0.45 µm pore size hydrophilic PVDF membrane, and immediately mixed with MEC1 cells and 10 µg/ml of polybrene (TR-1003-G, Sigma–Aldrich). 24 h later the medium was changed to remove the polybrene. To prepare the MEC1 cells for lentiviral infection, the same number as per HEK293 cells seeded for transfection was used, washed, and centrifuged 30 min at 200 rcf at RT (spinoculation).

### DNA pulldown followed by mass spectrometry or western blotting

Nuclear extracts from MEC1 were obtained as described on the Rockland Immunochemicals nuclear-extract protocol website (https://rockland-inc.com/Nuclear-Extract-Protocol.aspx) with some modifications: 40 million cells were washed twice with PBS, resuspended with 1 ml of Cytoplasmic Extract buffer (CE buffer: 10 mM HEPES, 60 mM KCl, 1 mM EDTA, 0.075% NP40, 1 mM DTT, 1 mM PMSF and 1X Pierce Protease Inhibitor (EDTA-free, Thermo Scientific), adjusted to pH 7.6) and centrifuged for 4 min at 600 rcf. The supernatant was removed, the pellet was resuspended with 500 μl of CE buffer without NP40, centrifuged again for 4 min at 600 rcf and the supernatant was removed. 100 μl of Nuclear Extract buffer (NE buffer: 20 mM Tris, 420 mM NaCl, 1.5 mM MgCl_2_, 0.2 mM EDTA, 1 mM PMSF, 25% glycerol, and 1x protease inhibitor, adjusted to pH 8.0.) was added and incubated on ice for 40 min vortexing 10 s every 10 min. Finally, the supernatant containing the nuclear extract was recovered after 10 min of centrifugation at 16,000 rcf.

The DNA pulldown was customized and performed based on previously described protocols^83–87^. HPLC purified oligos containing the ALT or REF alleles (Supplementary Table 3) were ordered from Thermo Scientific (forward oligo with 5’ biotin modification), resuspended in Annealing Buffer (10 mM Tris pH 7.5, 50 mM NaCl and 1 mM EDTA) and annealed (95 °C for 2 min and decreased to 4 °C at a 0.1 °C/s rate). 50 μl of Dynabeads MyOne Streptavidin C1 (Thermo Scientific) were washed twice with Biotin Binding buffer (BB buffer: 5 mM Tris pH 7.5, 0.5 mM EDTA, and 1 M NaCl). Beads were resuspended with 50 μl of fresh BB buffer and mixed with 10 μl of 50 pmol/μl double-stranded biotinylated probes 30–60 min at RT with constant agitation. Beads were washed twice in BB buffer and once with Protein Binding buffer (PB buffer: 20 mM Tris pH 7.5, 5% glycerol, 1 mM EDTA, 1 mM DTT, 0.15% Triton X-100, 100 mM NaCl, and 4 mM MgCl_2_). 450 μg of nuclear extract from MEC1 cells, 10 μg of poly(dA:dT) (tlrl-patn, InvivoGen) and 5 volumes of PB buffer with 1X protease inhibitor were added to the beads and incubated for 2 h with constant agitation (1 h 30 min at 4 °C and 30 min at RT). After incubation, beads were washed three times with 50 μl PB buffer without protease inhibitor and three times with 250 μl PB-MassSpectrometry buffer (PB-MS buffer: 20 mM Tris pH 7.5, 1 mM EDTA, 100 mM NaCl, and 4 mM MgCl_2_). Finally, proteins bound to the probes were eluted with 30 μl PB-MS containing 16 mM D-biotin (B20656, Thermo Scientific) after 10 min incubation at 37 °C with agitation. Samples (30 μl) were then run on a SDS-PAGE gel for either Western blotting (performed as described above) or mass spectrometry (MS): the gel was run until samples migrated 1 cm, stained 1 h at RT with SimplyBlue SafeStain (Thermo Scientific) and washed with water overnight at 4 °C. Gel lanes were washed twice in 50% ethanol and 50 mM ammonium bicarbonate (AB, Sigma–Aldrich) for 20 min and dried by vacuum centrifugation. The sample reduction was performed with 10 mM dithioerythritol (Merck-Millipore) for 1 h at 56 °C. A washing-drying step as described above was repeated before performing the alkylation step with 55 mM Iodoacetamide for 45 min at 37 °C in the dark. Samples were wash-dried again and digested overnight at 37 °C using mass spectrometry grade Trypsin at a concentration of 12.5 ng/µl in 50 mM AB and 10 mM CaCl_2_. The resulting peptides were extracted in 70% ethanol, 5% formic acid twice for 20 min with permanent shaking. Samples were further dried by vacuum centrifugation and stored at −20 °C. Peptides were desalted on C18 StageTips^88^ and dried by vacuum centrifugation. For Tandem Mass Tag (TMT; TMT10plex Isobaric Label Reagent Set, Thermo Scientific) labeling, peptides were first reconstituted in 8 μl HEPES 100 mM (pH 8.5) and 3 μl TMT solution (20 µg/μl in pure acetonitrile) was then added. Labelling was performed at room temperature for 1.5 h and reactions were quenched with hydroxylamine to a final concentration of 0.4% (v/v) for 15 min. TMT-labeled samples were then pooled at a 1:1 ratio across all samples. The combined sample was vacuum centrifuged near to dryness and subjected to fractionation using the Pierce High pH Reversed-Phase Peptide Fractionation Kit (Thermo Scientific) following the manufacturer’s instructions. The resulting fractions were resuspended in 2% acetonitrile, 0.1% FA and nano-flow separations were performed on a Dionex Ultimate 3000 RSLC nano UPLC system online connected with a Lumos Fusion Orbitrap Mass Spectrometer. A capillary precolumn (Acclaim Pepmap C18, 3 μm-100Å, 2 cm × 75 μm ID) was used for sample trapping and cleaning. Analytical separations were performed at 250 nl/min over 150 min biphasic gradients on a 50 cm long in-house packed capillary column (75 μm ID; ReproSil-Pur C18-AQ 1.9 μm silica beads; Dr. Maisch). Acquisitions were performed through the Top Speed Data-Dependent acquisition mode using a 3 s cycle time. First MS scans were acquired at a resolution of 120,000 (at 200 *m/z*) and the most intense parent ions were selected and fragmented by High energy Collision Dissociation (HCD) with a Normalized Collision Energy (NCE) of 37.5% using an isolation window of 0.7 m/z. Fragmented ions were acquired with a resolution of 50,000 (at 200 *m/z*) and selected ions were then excluded for the following 120 s. The mass tolerance for the precursors was 10 ppm and for the fragments ions, the tolerance was 0.02 Da. Raw data were processed using SEQUEST, Mascot, MS Amanda^89^, and MS Fragger^90^ in Proteome Discoverer v.2.4 (Thermo Scientific) against the Uniprot Human Reference Proteome (Uniprot Release: 2019_06). Enzyme specificity was set to Trypsin and a minimum of six amino acids was required for peptide identification. Up to two missed cleavages were allowed. A 1% FDR cutoff was applied both at peptide and protein identification levels. For the database search, carbamidomethylation (C), TMT tags (K and Peptide N termini) were set as fixed modifications, whereas oxidation (M) was considered as a variable one. The resulting text files were processed through in-house written R scripts (R v3.6.3). Two normalization steps were applied to the corrected reporter intensities: the sample loading normalization^91^ and a Trimmed M-Mean normalization using the R package edgeR^66^ v3.26.8. Differentially bound proteins were determined using the R bioconductor package limma^92^ v3.40.6, followed by FDR multiple-testing correction. A total of three replicates were obtained for both western blot and MS. MS was performed by the Proteomic Core Facility at EPFL.

### Genome editing

CRISPR/Cas9 genome editing was performed in MEC1 cells. In order to improve the efficiency in obtaining CRISPRed clones that underwent homologous recombination, we developed a system with one single plasmid containing all necessary components for gene editing (see Supplementary Fig. 8c for a schematic view of the CRISPR design).

#### Plasmid cloning

Two gRNAs (gRNA1 and gRNA2) were designed using the GPP sgRNA Designer tool from the Broad Institute on CRISPRko mode (https://portals.broadinstitute.org/gpp/public/). One nt G was added to the forward primer 5’ position if the gRNA itself did not start with a G (also added C on the reverse primer 3’ position). Additionally, overhanging DNA bases were added to the 5’ sites for cloning purposes (CACC on the forward and AAAC on the reverse primers). The forward and reverse gRNA primers were annealed and inserted in different pSpCas9(BB)-2A-GFP (PX458) (Addgene #48138)^93^ plasmids on the BbsI sites. The gRNA1 cassette was copied with the doublegRNA primers and inserted into gRNA2 containing PX458 plasmid on the KpnI site so one unique plasmid would express two different gRNAs. We cut the locus on two different sites since otherwise one homologous arm (HA) would be too far (>100 bp) from the cutting site. The donor template construct was homemade and consisted of two HA of 900 bp each encompassing a loxP–CMV–mCherry–T2A–puromycin resistance–WPRE–bGHpoly(A)–loxP–insert cassette. The insert region is the piece of genomic DNA that comprised the indel and was modified according to the genotype of interest. The left HA and the CA-rich region upstream of the indel were cloned from GM12878 genomic DNA. The CMV promoter and puromycin resistance sequences were copied from other plasmids. All other pieces comprising the insert (four different versions corresponding to REF, ALT, ALT.PU.1Δ, and MEF2Δ genotypes) and the right HA were bought as gBlocks from IDT. The donor template was placed in the PX458 plasmid expressing gRNAs 1 and 2 on the NotI site. In order to avoid mutations that could affect the CRISPR readout, first the plasmid with the REF genotype was obtained and was used as a backbone for the other genotypes by exchanging the insert region. To prevent Cas9 cutting the plasmid itself or after homologous recombination, we modified both protospacer adjacent motifs (PAMs) on the vector: gRNA1 PAM is truncated by the left loxP site and the gRNA2 PAM was mutated to introduce a de novo KpnI restriction enzyme site for screening purposes. We verified that these two modifications did not alter any TF motif using the RegulomeDB^94^. The whole donor template sequence was sequenced by Sanger (Microsynth) to make sure no other unwanted mutations were present. See Supplementary Table 3 for cloning and gRNA primer sequences and Supplementary Table 4 for the donor template DNA sequence.

#### CRISPR

5 million MEC1 cells were washed once with calcium and magnesium-free PBS and nucleofected with 35 μg of the corresponding plasmid using the Neon Transfection System 100 μl kit (Thermo Scientific) with the following reagents and parameters: R and E2 buffers, 2 pulses, 1100 V and 30 ms. Cells were immediately cultured with IMDM GlutaMAX with 15% FBS without antibiotics. 48 h later, 0.75 μg/ml puromycin selection was started until a sufficient number of resistant cells overgrew. After 1–2 weeks, GFP- and mCherry+ cells were sorted by FACS (Flow Cytometry Core Facility, EPFL) using FACSAriaII or FACSAriaFusion flow cytometers (BD Biosciences), and single cells were dispensed into 384-well plates containing conditioned medium and 0.75 μg/ml puromycin; plates were sealed with parafilm and incubated until a sufficient number of cells were produced. Conditioned medium was obtained by mixing 50:50 of fresh 10% FBS 1% P/S IMDM with 0.45 μm filtered medium that was used to culture MEC1 cells for 1–2 days, plus an additional 10% FBS. Single-cell clones were PCR genotyped using primers that were specific for the genomic region outside of the HAs, surrounding the donor template, so clones that underwent homologous recombination for both alleles would have a single band of ~6 kb instead of 3 kb (Left_Right.gt primers). A set of primers (CRISPR.background) that amplify a region of the PX458 backbone was used to discard clones that integrated the plasmid unspecifically. Good clones were infected with lentiviruses expressing Cre recombinase and blasticidin resistance. The pLV-Cre plasmid was a generous gift from Dr. Jiahuai Han from Xiamen University. After 1–2 weeks of selection with 5 μg/ml blasticidin, the second round of single-cell cloning was performed as previously described but selecting mCherry- cells. Clones were genotyped using the same primer set Left_Right.gt (expected band of ~3 kb). To make sure that both alleles were correct, we also tested the clones by KpnI restriction enzyme digestion of Axin2gt PCR products. For each genotype, we obtained more than one clone from the first single-cell cloning round, and multiple clones from the second round. Most of the clones were verified by Sanger sequencing (Microsynth) with Axin2gt PCR products and we removed those that had aberrant DNA sequences.

### ChIP-qPCR

Three different ALT clones and three different REF clones from the CRISPRed MEC1 cells with an *AXIN2* mRNA expression close to the average were selected and mixed in equal proportions for the experiment. 20 million cells were fixed with 2% paraformaldehyde in PBS for 10 min at RT under rotation. Fixation was stopped by adding glycine at a final concentration of 125 mM and incubated for 5 min at RT under rotation. Cells were then washed twice with ice-cold PBS and snap-frozen in liquid nitrogen. Cells were then resuspended in 1.5 mL ChIP Lysis buffer (50 mM Hepes pH 7.8, 140 mM NaCl, 1 mM EDTA pH 8, 0.5% NP40, 10% Glycerol, 0.25% Triton and supplemented with proteinase inhibitors (Thermo Scientific, A32965)) and incubated for 10 min at 4 °C under rotation. Cells were spun down, resuspended in 1.5 mL Nuclei Wash buffer (20 mM Tris-HCl pH 8, 200 mM NaCl, 1 mM EDTA pH 8, 0.5 mM EGTA pH 8, and supplemented with proteinase inhibitors), and incubated for another 10 min at 4 °C under rotation. Finally, nuclei were spun down and resuspended in 1 mL Sonication buffer (20 mM Tris-HCl pH 8, 200 mM NaCl, 1 mM EDTA pH 8, 0.5 mM EGTA pH 8, 0.5% Na Deoxycholate, 0.5% N-lauroylsarcosine and supplemented with proteinase inhibitors). Nuclei were then sonicated using a Covaris E220 with the following settings: 15 min, 140 W of intensity, 5% duty factor, and 200 bursts/cycle. After sonication, 50 μl of 20% Triton was added. The sonicated chromatin was then spun at max speed in a microcentrifuge to remove the debris. 10 μl was taken at this step for the input and the remaining chromatin was snap-frozen. Five hundred microliters of the chromatin was used for the MEF2C ChIP and the other 500 μl for the NoAb ChIP control. Five hundred microliters of the chromatin was incubated with 10 μl BSA blocked Dynabeads Protein G (Thermo Scientific) for 2 h at 4 °C under rotation to remove the unspecific binding. Beads were discarded and the chromatin was incubated with 10 μl of MEF2C antibody (#5030, Cell Signaling) and 25 μl BSA blocked Dynabeads Protein G for 2 h at 4 °C under rotation. Beads were washed 5 min at RT under rotation twice with 1 ml low salt buffer (20 mM Tris-HCl pH 8, 150 mM NaCl, 2 mM EDTA pH 8, 1% Triton X-100, 0.1% SDS), twice with 1 ml high salt buffer (20 mM Tris-HCl pH 8, 500 mM NaCl, 2 mM EDTA pH 8, 1% Triton X-100, 0.1% SDS), twice with 1 mL LiCl buffer (10 mM Tris-HCl pH 8, 250 mM LiCl, 1 mM EDTA pH 8, 0.5% NP40, 0.5% Na Deoxycholate) and once with 1 ml TE buffer (10 mM Tris-HCl pH 8, 1 mM EDTA pH 8). Beads were finally resuspended in 100 μl elution buffer (10 mM Tris-HCl pH 8, 1 mM EDTA pH 8, 1% SDS, 200 mM NaCl) and incubated for 10 min at 65 °C. The resulting supernatant was incubated at 65 °C for 5 h with 0.4 μg/μl proteinase K. DNA was purified with DNA Clean & Concentrator-5 (Zymo Research). qPCR was performed with three sets of primers: a negative control region (2 kb from the *RPLP0* TSS), the indel region, and a positive control region (*RPLP0* TSS) (see Supplementary Table 3 for the list of primers), and the percentage enrichment based on the input was assessed. A total of 6 biological replicates was performed.

### ATAC-seq

Three different ALT clones and three different REF clones from the CRISPRed MEC1 cells with an *AXIN2* mRNA expression close to the average were selected and mixed in equal proportions for the experiment. 50,000 cells were washed with PBS, resuspended in 50 μl RSB buffer (10 mM Tris-HCL pH 7.4, 10 mM NaCl, 3 mM MgCl_2_, 0.1% NP40, 0.1% Tween20) and incubated on ice for 3 min. Cells were spun down, the supernatant was discarded, cells were resuspended in 1 ml RBS buffer without NP40, inverted three times, and spun down. The supernatant was discarded, cells were resuspended in 25 μl 2× Tagmentation buffer (20 mM Tris-HCl pH 7.6, 10 mM MgCl_2_, 20% Dimethyl Formamide), 0.5 μl 10% Tween20, 16.5 μl PBS, 1 μl of homemade 24.5 μM Tn5 and 7 μl H_2_O and incubated 30 min at 37 °C rotating at 1000 rpm. DNA was then purified with a DNA Clean & Concentrator-5 (Zymo Research). Barcoded DNA libraries were then prepared by amplifying a total of 11 cycles, as described in^95^. The libraries were then paired-end sequenced (2 × 75 cycles) on a HiSeq 4000 sequencer (Illumina). Fastq files were aligned to hg19 with BWA-MEM^70^ v.0.7.17-r1188, duplicates were removed with Picard v22.2.9 (http://broadinstitute.github.io/picard/), peaks called with MACS2^96^ v2.1.2 (--nomodel -q 0.05) and BigWig files were generated with bamCoverage^65^ v3.5.0 (10 bp bin size and RPKM normalized). Reads per peak were obtained with HOMER^67^ v4.11 (without normalization) using meta peaks obtained from merging REF and ALT data, read counts were multiplied by two (given that HOMER counts fragments as half for paired-end data) and normalized with the DESeq2^69^ v1.26.0 R package counts function.

### Luciferase reporter assays

Genomic DNA from GM12282 and GM11931 was extracted using the Quick-DNA Miniprep kit (Zymo Research) and used as a template to amplify the DNA sequence of 490 bp (REF) or 485 bp (ALT) centered on rs143348853, respectively. The PCR products were inserted using the BamHI and SpeI sites in the STARR-seq luciferase vector (#99297, Addgene). We found that there is a second genetic variant that differs between the two LCLs in the region of interest (rs11867847); in order to modify this polymorphism, the REF template was amplified in two pieces using a second pair of primers (site-directed mutagenesis). See Supplementary Table 3 for the list of primers. 500,000 MEC1 cells were washed once with calcium and magnesium-free PBS and nucleofected with 1 μg of the luciferase plasmids (ALT, REF, or empty vector) and 1 μg of a plasmid expressing Renilla Luciferase (pRL-TK E2241, Promega) using the Neon Transfection System 10 μl kit (Thermo Scientific) with the following reagents and parameters: R and E buffers, 2 pulses, 1100 V and 30 ms. Cells were immediately cultured with IMDM GlutaMAX with 10% FBS without antibiotics. 24 h later, luciferase expression was measured using the Dual-Luciferase Reporter Assay System (Promega). A total of 4 biological replicates were obtained.

### NGS Capture-C

20 M GM12282 (homozygous REF) or GM11931 (homozygous ALT) cells were fixed with 2% PFA in PBS at RT for 10 min. Fixation was stopped by adding glycine at a final concentration of 125 mM and incubated for 5 min at RT under rotation. Cells were then washed twice with ice-cold PBS, resuspended in lysis buffer (10 mM Tris-HCl pH 8, 10 mM NaCl, 0.2% NP40, and supplemented with 1× complete protease inhibitor (Roche)), incubated 20 min on ice, and snap-frozen with the buffer. The Capture-C was performed as described in^51^ with the following modifications: NlaIII restriction enzyme (New England Biolabs) was used and incubated for 24 h. Ligation was performed for 22 h. 5 μg of DNA (3 C library) in 120 μl TE was sonicated using a Covaris E220 with the following settings: 180 s, 10% duty factor, 175 W of intensity, 200 bursts/cycle with the #500141 intensifier (Covaris). 1 μg of sonicated DNA was used to prepare the library using the NEBNext Ultra II DNA Library Prep Kit for Illumina (New England Biolabs) as described by the manufacturer. After adapter ligation, DNA was cleaned-up using 1.8X AMPure X beads (Beckman) and amplified with Herculase II (Agilent) for 6 cycles. Each biological replicate (3 biological replicates per cell line) was labeled with a different barcode and all samples (a total of 6) were pooled together (2 μg of each). For the first capture, 2.9 nM of each biotinylated probe (two probes targeting both extremes of the rs143348853 containing fragment, Supplementary Table 3) was used and incubated for a total of 72 h. The pooled captured library was amplified for 14 cycles and a second capture was performed with all resulting DNA (130 ng) and 2.9 nM of each biotinylated probe for 24 h. The pooled library was further amplified for 14 additional cycles. The NGS Capture-C library was then sequenced on a HiSeq 4000 (Illumina) in paired-end mode, 150 cycles. Fastq files were processed as follows: adapters were trimmed with Trim Galore v0.6.7 (https://www.bioinformatics.babraham.ac.uk/projects/trim_galore/), paired-end reads were reconstructed using FLASH v1.2.11^97^ with the interleaved-output setting and reads were digested with a modified version of the DpnII2E.pl script to cut NlaIII sites (the genome was also digested with the NlaIII version of dpngenome3_1.pl script) (https://github.com/Hughes-Genome-Group/captureC/releases). Digested fastq files were aligned to hg19 using BWA-MEM^70^ v.0.7.17-r1188 using the -t flag 1 (single thread). Finally, SAM files were processed using the CCanalyser3.pl script (https://github.com/Hughes-Genome-Group/captureC/releases). All GFF files obtained from the CCanalyser3.pl were processed as follows: counts were normalized by total counts in the *AXIN2* TAD, and the viewpoints within ±1 kb from the Capture probe were excluded from further analyses or plots. For the differential interaction analysis, the TAD was binned in 5 kb windows and counts of all fragments falling within each bin were aggregated prior to running DESeq267^69^ v1.26.0 with standard parameters. For assessing the overall interaction-count distribution within the *AXIN2* TAD, all three replicates were merged at the fastq level. Barplots were generated using the GenomicInteractions^98^ v1.16.0 R package.

### ORCA

The primary probes tiling a 200 kb region that encompasses the *AXIN2* gene (hg19 chr17:63,486,119-63,686,118) at 2-kb resolution, were designed as previously described^52^ (Supplementary Data 4). In order to increase the number of probe sequences within each 2-kb region, we allowed the primary probes to overlap in their genomic targeting sequence within each 2-kb segment. Probes were amplified from the oligopool (CustomArray), and amplified according to the protocol described in Boettiger et al., 2016^99^ and Mateo et al., 2019^52^. In preparation for ORCA, homozygous REF (GM12282) and homozygous ALT (GM11931) LCL cells were fixed in suspension with 4% PFA for 10 min. Following 3 washes in 1×PBS, cells were resuspended in 70% EtOH in 1×PBS and stored at −20 °C. For cell hybridization, cells were plated on Poly-D-Lysine coated slides. In order to minimize technical variation, we plated GM12282 and GM11931 in spatially distinct spots on the same slide. The hybridization and imaging were performed as previously described^52^. Briefly, for primary probe hybridization, cells were permeabilized for 10 min with 0.5% Triton X in 1X PBS, the DNA was then denatured by treatment with 0.1 M HCl for 5 min, followed by incubation in hybridization buffer (50% formamide, 0.1% tween). two micrograms of primary probes in hybridization solution (50% formamide, 10% dextran sulfate, 0.1% tween, 2× saline-sodium citrate buffer (SSC)) was then added directly on to cells, placed on a heat block for 90 °C for 3 min and incubated overnight at 42 °C in a humidified chamber. Prior to imaging, the samples were post-fixed for 35 min to 1 h in 8% PFA + 2% glutaraldehyde (GA) in 1X PBS. The samples were then washed in 2xSSC and either imaged directly or stored for up to a week at 4 °C prior to imaging. For imaging, samples were mounted into a Bioptechs flow chamber, and secondary probe hybridization and step by step imaging of barcodes was performed as in Mateo et al., 2019^52^. However, due to the low signal intensity of individual barcodes, each step combined 4 barcodes, yielding a genomic resolution of 8 kb (25 segments in total, coordinates listed in Supplementary Table 5). Image processing (drift correction and localization of spots) analysis was performed as described in Mateo et al., 2019^52^. The GM12878 HiC interaction frequency data^53^ for the *AXIN2* locus in Fig. 6e was extracted from Juicebox^100^. For Fig. 6g and Supplementary Fig. 9e, values were obtained by subtracting the REF population-median distance values from ALT. Enrichment of further or closer interactions was assessed with a two-sided binomial test, taking into account changes different than 0. Single-cell 3D reconstruction of chromatin structure represented in Fig. 6h was obtained using in-house^52^ Matlab R2019a scripts employing the segment xyz coordinates (missing values in between segments were inferred to build a continuous polymer), cells were chosen to have the same number of valid 3D segment coordinates (*n* = 21).

### AXIN2 overexpression in MEC1 cells

The *AXIN2* ORF was obtained and copied from the vector pDONR223_AXIN2_WT (#82099, Addgene)^101^. Given that this *AXIN2* ORF contains Serine instead of Proline in position 50 (due to the polymorphism rs2240308), we cloned the ORF from two parts with primers overlapping the SNP (Supplementary Table 3) to change the genotype to the reference allele according to NCBI dbSNP^23^ build 154 (i.e. nucleotide G, aminoacid Proline). We inserted the ORF in pLV-EF1a-IRES-Puro (#85132, Addgene)^102^ between the BamHI and MluI sites. Lentiviruses for empty (control) or *AXIN2* ORF containing vectors were produced to infect MEC1 cells. After 24 h after infection, selection with 0.75 μg/ml puromycin was started and kept under selection for 1–2 weeks.

### mRNA-seq

RNA from 500,000 MEC1-ctr and MEC1-AXIN2 cells was extracted using the Direct-zol RNA Miniprep kit (Zymo Research) with 15 min on-column DNase digestion at RT. Eight hundred nanograms of total RNA were used to synthesize the libraries with Truseq Stranded mRNA LT kit (Illumina) and sequenced on a HiSeq 4000 (Illumina) in paired-end mode, 150 cycles. Reads were aligned to hg19 using STAR^103^ v2.7.3a (--outSAMmapqUnique 60 --outFilterMultimapNmax 1 --outSAMtype BAM Unsorted). Read counts per transcript were assessed using htseq-count^68^ v0.12.4 (-s reverse -m union) with the gtf file downloaded from Ensembl, GRCh37 release 75. Genes with <1 read in all samples were discarded and differentially expressed genes were obtained with the R Bioconductor package DESeq2^69^ v1.26.0. Gene ontology analysis was performed on all significantly differentially expressed genes (FDR < 0.05) using the R Bioconductor package TopGO v2.38.1. PCA plot was obtained using plotPCA function on varianceStabilizingTransformation transformed counts (DESeq2^69^ v1.26.0). To obtain Wnt pathway-related genes we used the BiomaRt^104,105^ v2.42.1 R Bioconductor package. To plot the heatmap (R pheatmap v1.0.12 package), raw read counts were normalized by FPKM (edgeR^66^ v3.28.1) and percentages were calculated based on the mean of the MEC1-ctr samples for each gene (genes and samples clustered by Euclidean distances).

### In vitro cell proliferation assays

We tested the differential growth rate of the MEC1-ctr or MEC1-AXIN2 overexpression cells in vitro using the ViaLight Plus Cell Proliferation and Cytotoxicity BioAssay Kit (Lonza) following the manufacturer’s instructions. We seeded 2,000 cells per well on a 96-well plate to as many wells as five technical replicates x time points to analyze. Right after seeding we took the first measure (Day 0). For the final analysis, a technical replicate was discarded if its value was beyond half or double the average value of the five technical replicates for that data point. The experiment was performed in 3 biological replicates. Significance was tested by two-sided unpaired Welch corrected *t*-test.

### In vivo competitive growth of MEC1-ctr and MEC1-AXIN2 overexpression cells

In order to label the two different MEC1 cell populations (ctr or AXIN2-overexpressing cells) with two different fluorescent proteins for the in vivo study, we used the pLV-mCherry plasmid (#36084, Addgene). To obtain the pLV-GFP plasmid using the same backbone, we exchanged the mCherry gene with a PCR product containing GFP from #17448 (Addgene)^106^, using the BamHI and SalI sites. Lentiviral production and infection were performed as described above in order to label MEC1-ctr with GFP and MEC1-AXIN2 with mCherry. Finally, fluorescent cells were sorted by FACS (Flow Cytometry Core Facility, EPFL) using FACSAriaII or FACSAriaFusion flow cytometers (BD Biosciences).

Cells were harvested, washed twice with PBS, and a mixture of 50:50 MEC1-ctr-GFP and MEC1-AXIN2-mCherry cells was prepared in PBS with a concentration of 5 million cells per 100 μl. 200 μl of this solution were injected intravenously by the tail vein into each mouse for a total of 10 NSG (NOD.Cg-*Prkdc*^*scid*^ *Il2rg*^*tm1Wjl*^/SzJ, The Jackson Laboratory) ~12–14 weeks old male mice per experiment. Prior to the injection, we analyzed the percentage of the respective cell populations in the input by flow cytometry using the LSR Fortessa (BD Biosciences) and the BD FACSDiva software v8.0.2. After 26 days, the mice were sacrificed and bone marrow immune cells from both legs (femur and tibia) and hip bones were extracted. Single-cell suspensions were prepared as previously described using standard procedures^107^. Cells were stained with 1:200 human CD20 PE/Cy7 (302312, BioLegend), 1:800 mouse CD45 APC (17-0451-83, eBioscience), and 1:5000 DAPI, and analyzed by flow cytometry. Data acquisition was performed using the FACSAria III sorter (BD Biosciences) (Flow Cytometry Facility, UNIL) using the BD FACSDiva software v8.0.2. and at least 20,000 GFP + and 20,000 mCherry+ events were acquired. Data were analyzed using FlowJo TreeStar software v10.7.1(BD Biosciences). The percentage of GFP+ and mCherry+ cells was determined and the fold change respective to the input percentage was calculated. The experiment was repeated twice.

To test the effect of the fluorescent protein in MEC1 growth, we performed a similar experiment but exchanging mCherry and GFP, by infecting MEC1-ctr cells with pLV-mCherry and MEC1-AXIN2 with pLV-GFP. We used 3 mice with a mixture of 50:50 MEC1-ctr-mCherry and MEC1-AXIN2-GFP, and 3 mice with a mixture of 50:50 MEC1-ctr-GFP and MEC1-AXIN2-mCherry. 25–26 days post-injection, cells were stained with 1:200 human CD20 PE/Cy7 (302312, BioLegend), 1:800 mouse CD45 APC (17-0451-83, eBioscience), and 1:1000 Zombie NIR (BioLegend), and analyzed by flow cytometry using the LSR Fortessa (BD Biosciences). Mice were bred and maintained at the EPFL animal facility. They were housed in individual cages at 23 °C ± 1 °C with a 12 h light/dark cycle. All animals were supplied with food and water ad libitum. All animal work was carried out in accordance with Swiss national guidelines. This study was reviewed and approved by the cantonal veterinary service, Vaud.

### Quantification and statistical analysis

All statistical tests were performed on R v3.6.2 (except specified), GraphPad Prism 8, and Matlab R2019a. R Bioconductor v3.10 was used.

### Reporting summary

Further information on research design is available in the Nature Research Reporting Summary linked to this article.

## Supplementary information

Supplementary Information

Description of Additional Supplementary Files

Supplementary Data 1

Supplementary Data 2

Supplementary Data 3

Supplementary Data 4

Reporting Summary

## Data Availability

RNA-seq data from MEC1-AXIN2 overexpression or ctr MEC1 cells, ATAC-seq data from CRISPRed MEC1 cells, and Capture-C data from LCLs have been deposited into the Gene Expression Omnibus (GEO) repository (accession number GSE162387). The mass spectrometry proteomics data have been deposited to the ProteomeXchange Consortium via the PRIDE partner repository with the dataset identifier PXD029313 [proteomecentral.proteomexchange.org/cgi/GetDataset?ID = PXD029313]. Study-related UNIUPO data are available upon request whereas source data are provided with this paper. Publicly available data described in Methods: E-MTAB-3657 (LCL ChIP-seq data), E-MTAB-3656 (LCL RNA-seq data), EGAS00001003485 (LCL ChIP-seq and RNA-seq), E-MTAB-3657 (LCL ChIP-seq and RNA-seq), ERP110508 [https://www.ebi.ac.uk/ena/browser/view/PRJEB28318] (LCL ATAC-seq), https://zenodo.org/record/1405945#.X7PXCMco-uM (processed LCL ATAC-seq data), EGAD00001004046 (CLL Blueprint data), ENSEMBL (https://www.ensembl.org/index.html), Roadmap Epigenomics project (www.roadmapepigenomics.org), ENCODE (https://www.encodeproject.org), HOCOMOCO (https://hocomoco11.autosome.ru/), The 1000 Genomes Project (https://www.internationalgenome.org/), GM12878 phased genotype data from the Genome In a Bottle consortium (ftp-trace.ncbi.nlm.nih.gov/giab/ftp/release/NA12878_HG001/latest/GRCh37/), dbGaP Study Accession phs000178.v11.p8, International Cancer Genome Consortium (ICGC) (https://dcc.icgc.org/), EGAD00001005970 (CLL whole-genome bisulfite sequencing data), EGAS00001000534 (healthy donor B cells Whole-genome bisulfite sequencing data), UCSC Genome Browser (https://genome.ucsc.edu/), FinnGen (http://r4.finngen.fi/variant/17-65564173-AAAATC-A), ERP002168 [https://www.ebi.ac.uk/ena/browser/view/PRJEB1350] (LCL CTCF ChIP-seq), RegulomeDB (https://regulomedb.org/regulome-search/), NCBI dbSNP build 154 (https://www.ncbi.nlm.nih.gov/snp/), 3D Genome Browser (http://3dgenome.fsm.northwestern.edu/tutorial.html), TF list^42^ and B cell developmental enhancers^29^. Other publicly available data: The GTEx Portal (https://gtexportal.org/home/index.html) was accessed on April 15, 2020 to obtain rs143348853 eQTL and *AXIN2* expression information across multiple cell types. Previously determined TAD coordinates from the GM12878 LCL were obtained from Beekman et al., 2018^72^, which used HiC data from Rao et al., 2014^53^. For the same cell line, we also obtained CTCF ChIA-PET loop information from Tang et al., 2015^49^, using the GSM1872886_GM12878_CTCF_PET_clusters.txt file corresponding to GEO sample GSM1872886. To visualize the CTCF ChIA-PET loops, we used the Bioconductor R packages GenomicRanges^73^ v1.38.0, GenomicInteractions^98^ v1.16.0 and Gviz^108^ v1.30.3. H3K27ac and Input ChIP-seq data for OSU-CLL and MEC1 cell lines were retrieved from Ott et al. 2018^109^ (GEO accession number GSE119744): fastq files were aligned with bwa mem v0.7.17^70^ to hg19, converted to the BAM format and sorted using SAMtools^71^ v1.9, read duplicates were removed with the MarkDuplicates function from Picard v22.2.9, ENCODE blacklisted regions were discarded with Bedtools^75^ v2.27.1, peaks called with MACS2^96^ v2.1.2 with Input ChIP-seq as control (--nomodel -q 0.05 --broad), and bigWig files generated with bamCoverage^65^ v3.5.0 (10 bp bin size and RPKM normalized). When required to compare datasets from different genome versions, we used the liftOver function from the UCSC Genome Browser^24^ or in R (rtracklayer^74^ v1.46.0 Bioconductor package) to convert them to hg19 coordinates. Source data are provided with this paper.

## Source data

Source Data
