# Supplementary material for: A leukemia-protective germline variant mediates chromatin module formation via transcription factor nucleation
Source: Nat Commun. 2022 Apr 19;13:2042. doi: 10.1038/s41467-022-29625-6 (PMC9018852; doi:10.1038/s41467-022-29625-6)
Supplement: Supplementary file 1 — Supplementary Information [file 41467_2022_29625_MOESM1_ESM.pdf]

SUPPLEMENTARY INFORMATION

Supplementary Figure 1.
Supplementary Figure 2.
Supplementary Figure 3.
Supplementary Figure 4.
Supplementary Figure 5.
Supplementary Figure 6.
Supplementary Figure 7.
Supplementary Figure 8.
Supplementary Figure 9.

Supplementary Table 1.
Supplementary Table 2.
Supplementary Table 3.
Supplementary Table 4.
Supplementary Table 5.

SUPPLEMENTARY REFERENCES

Supplementary Fig. 1

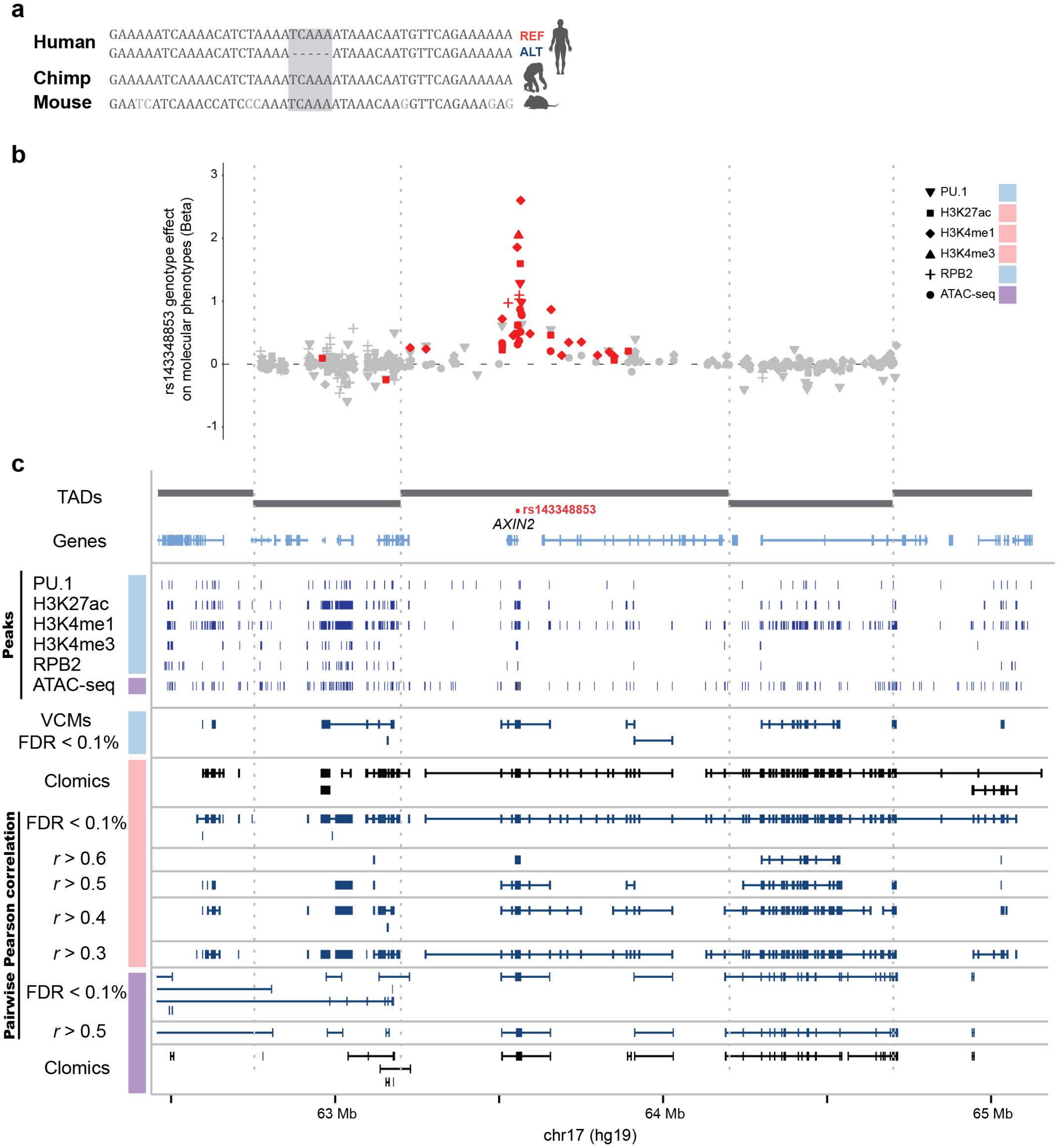

Data:
S. Waszak, O. Delaneau et al. Cell 2015
PU.1, H3K27ac, H3K4me1, H3K4me3 and RPB2 ChIP-seq
47 individual LCLs

O. Delaneau et al. Science 2019
H3K27ac, H3K4me1 and H3K4me3 ChIp-seq
313 individual LCLs

N. Kumasaka et al. Nat. Genet. 2018
ATAC-seq
100 individual LCLs

Supplementary Fig. 1. *AXIN2* VCM characteristics. a DNA sequences of the rs143348853 locus for human, chimp and mouse. **b** Beta values, obtained by a linear regression model, representing the genetic effect of rs143348853 for each studied molecular phenotype (FDR < 0.05 values colored in red). **c** IGV[1] view of the *AXIN2* locus showing GM12878 TADs[2], meta peaks for all studied molecular phenotypes and VCMs calculated from different datasets using either Pearson correlation values between peak pairs with different thresholding methods (*r* coefficient or FDR value)[3] or a hierarchical clustering-based approach (Clomics)[4].

Supplementary Fig. 2

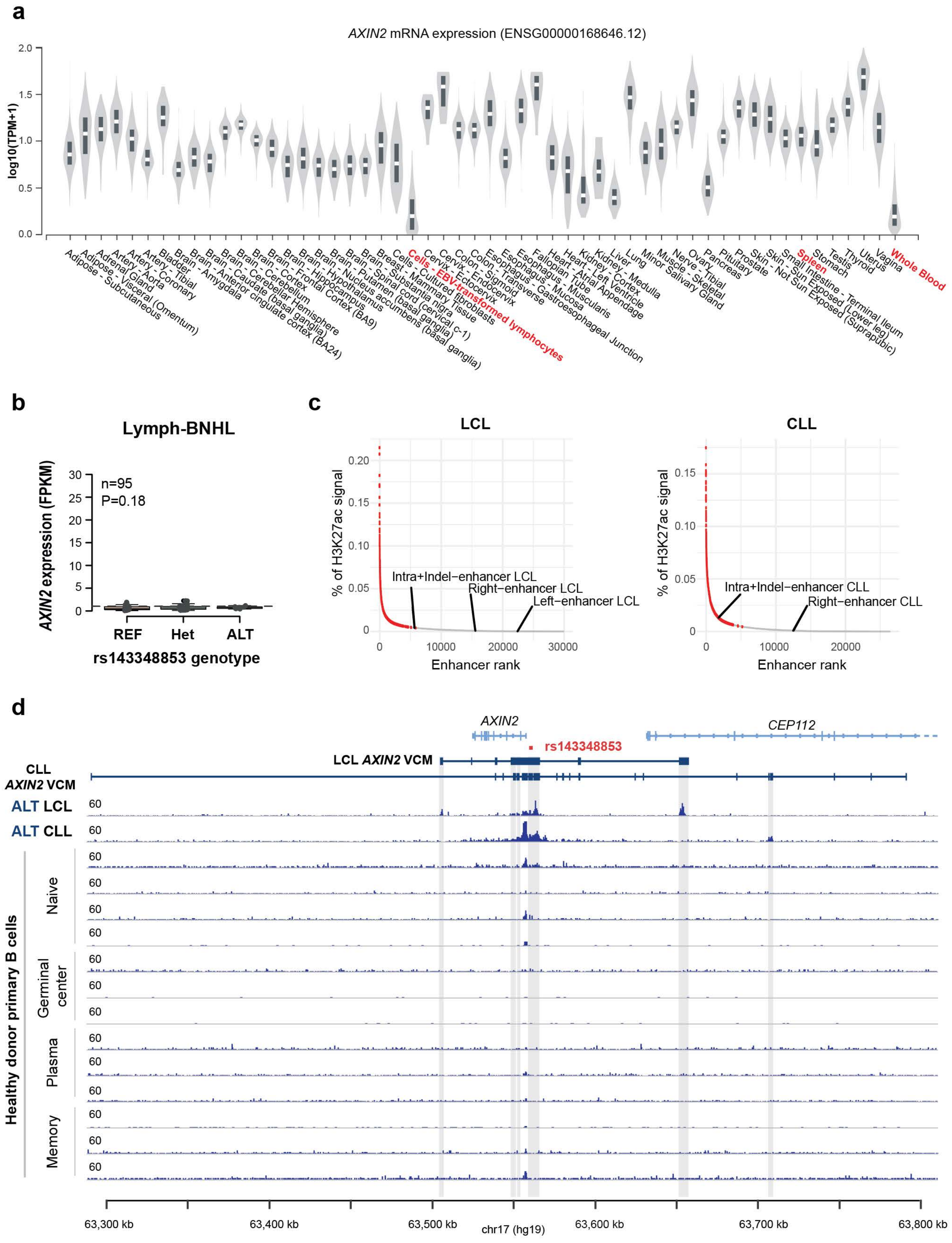

Supplementary Fig. 2. *AXIN2* expression and H3K27ac enrichment across different cell types and rs143348853 genotypes. a *AXIN2* mRNA expression across different cells or tissues from the GTEx portal (TPM accounts for transcripts per million). Significant hits (Bonferroni adjusted *p* value < 0.05) denoted in **Fig. 2a** are labelled in red. **b** *AXIN2* mRNA expression for the different rs143348853 genotypes of B cell Non-Hodgkin lymphoma (Lymph-BNHL) cells from PCAWG; *n* and P indicate the number of considered patients and nominal *p* value from a linear regression model, respectively. Boxes indicate the IQR (25-75%) and the box center indicates the median. Whiskers represent de minimum or maximum values of no further than 1.5 times the IQR for both the top and bottom of the box. **c** Rank-ordered enhancers based on H3K27ac signal from LCLs and CLLs. The average rank from four different homozygous ALT LCL and CLL cells was taken. Enhancers denoted as super-enhancers for at least one individual by ROSE2 are highlighted in red. **d** H3K27ac ChIP-seq signal across the *AXIN2* locus from an ALT LCL, an ALT CLL tumor and healthy donor primary B cells, to illustrate enhancer distribution differences among these B cell classes. The *AXIN2* LCL and CLL VCMs, as inferred using the pairwise correlation method, are also shown. Grey boxes indicate LCL and CLL enhancers.

Supplementary Fig. 3

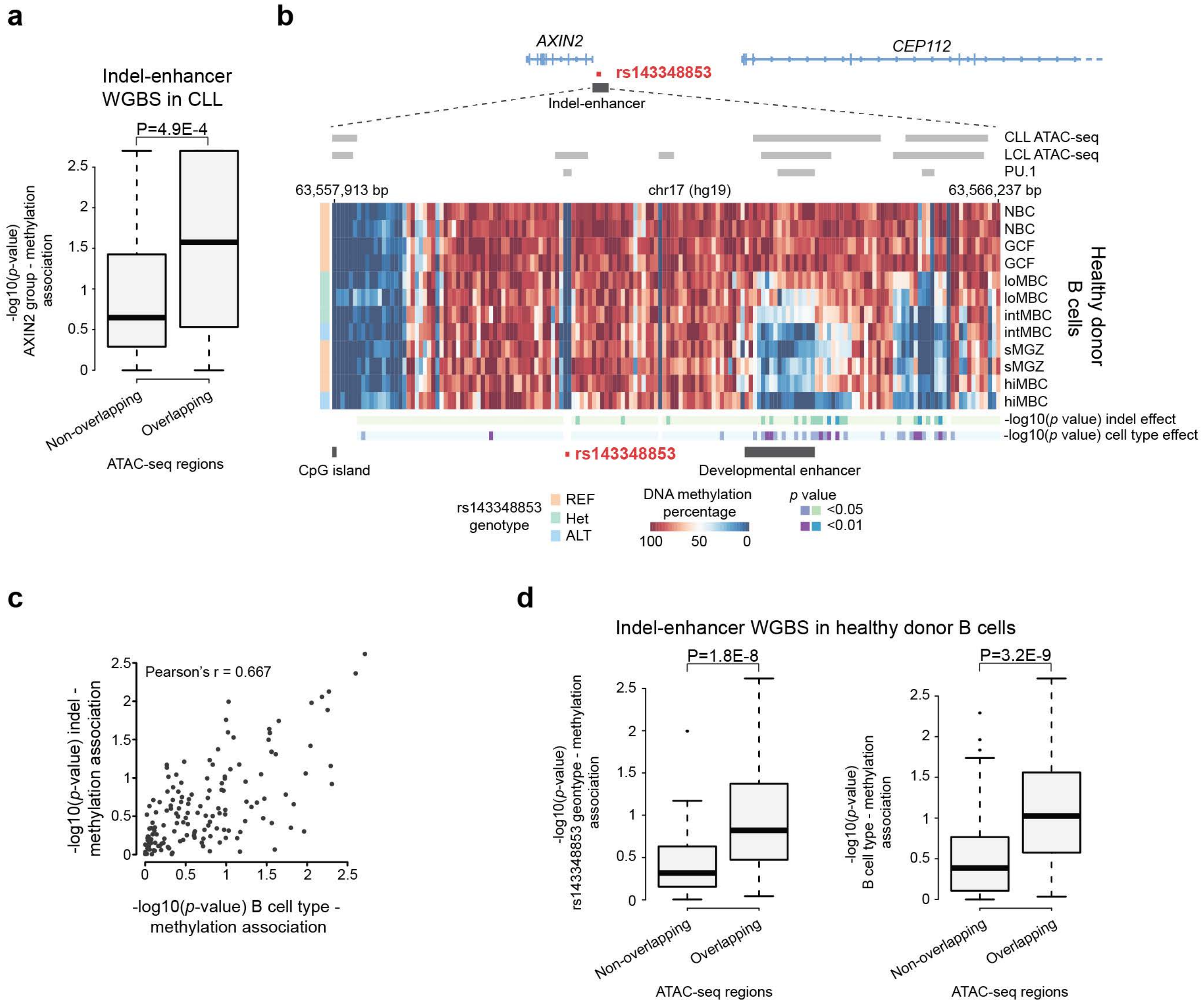

Supplementary Fig. 3. Indel-enhancer predisposition to activation during B cell development. **a** -Log10(nominal *p* value) for each region from an *AXIN2* group-methylation association using a linear regression model in CLL is displayed and categorized as non-overlapping (n = 92) or overlapping (n = 70) ATAC-seq peaks (LCL and CLL merged peaks). Only regions embedded in the indel-enhancer were assessed. P indicates *p* value from a two-sided Wilcoxon test. Boxes indicate the IQR (25-75%) and the box center indicates the median. Whiskers represent de minimum or maximum values of no further than 1.5 times the IQR for both the top and bottom of the box. **b** DNA methylation on the indel-enhancer region from different B cell types during B cell development: naive (NBC), germinal center founder (GCF), early non-class-switched memory (loMBC), non-class-switched memory (intMBC), splenic marginal zone (sMZB) and class-switched memory (hiMBC) B cells. CLL and LCL ATAC-seq and LCL PU.1 peaks are displayed. CpG island coordinates were obtained from the UCSC Genome Browser. The region found to be in the top 5000 most hypomethylated regions in later stages of B cell development compared to NBCs[5] (i.e. developmental enhancer) is also denoted. Nominal *p* values, based on the rs143348853 genotype- or the cell type-methylation associations using a linear regression model, are denoted in the *p* value tracks. **c** -Log10 nominal *p* values for each region in the indel-enhancer from the rs143348853 genotype-methylation and B cell type-methylation associations, using a linear regression model. **d** Same as **a** but in healthy donor B cells for the rs143348853 genotype-methylation (left) and cell type-methylation (right) associations (n = 90 for non-overlapping and n = 61 for overlapping ATAC-seq peaks). P indicates *p* value from a two-sided Wilcoxon test. Boxes indicate the IQR (25-75%) and the box center indicates the median. Whiskers represent de minimum or maximum values of no further than 1.5 times the IQR for both the top and bottom of the box.

Supplementary Fig. 4

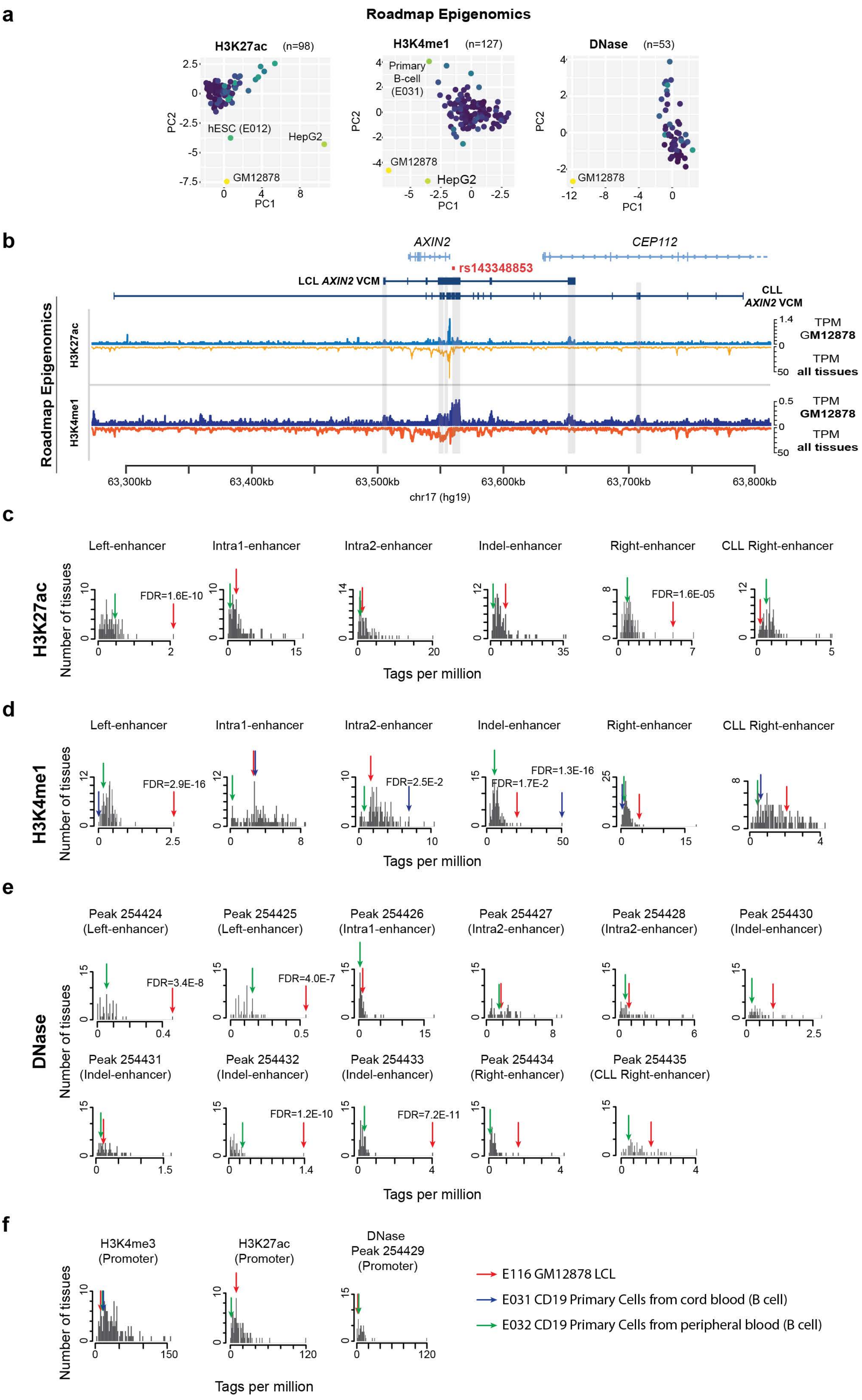

Supplementary Fig. 4. Specificity of the *AXIN2* enhancer cluster. a PCA plots showing enhancer composition dissimilarity based on H3K27ac, H3K4me1 and DNase signals from the Roadmap Epigenomics project data, colored according to the local outlier factor (LOF). The most distinguishable outliers are labelled (E031 indicates the cell type id provided by Roadmap). *n* indicates the number of considered tissues. **b** Genomic view of Roadmap H3K27ac and H3K4me1 signals as tags per million (TPM) for GM12878 and the sum of all different cell types. The LCL and CLL *AXIN2* VCM enhancer regions are marked with grey boxes. **c**,**d**,**e** Histogram representation of tags per million for all available tissue/cell types from Roadmap molecular phenotypes: H3K27ac (**c**), H3K4me1 (**d**) and DNase (**e**) on the different LCL and CLL *AXIN2* enhancers. For DNase, assessed regions represent LCL ATAC-seq peaks. **f** Roadmap H3K4me3, H3K27ac and DNase signals on the *AXIN2* promoter. Only B cell samples were considered for the enrichment test: GM12878 (E116) (red), CD19+ primary cells from cord blood (E031) (blue), CD19+ primary cells from peripheral blood (E032) (green). For **a-f**, cell ids as labelled by Roadmap. Significantly enriched hits (FDR < 0.05) are annotated with their FDR value, which indicates multiple testing corrected one-sided *p* value obtained from the R pnorm function.

Supplementary Fig. 5

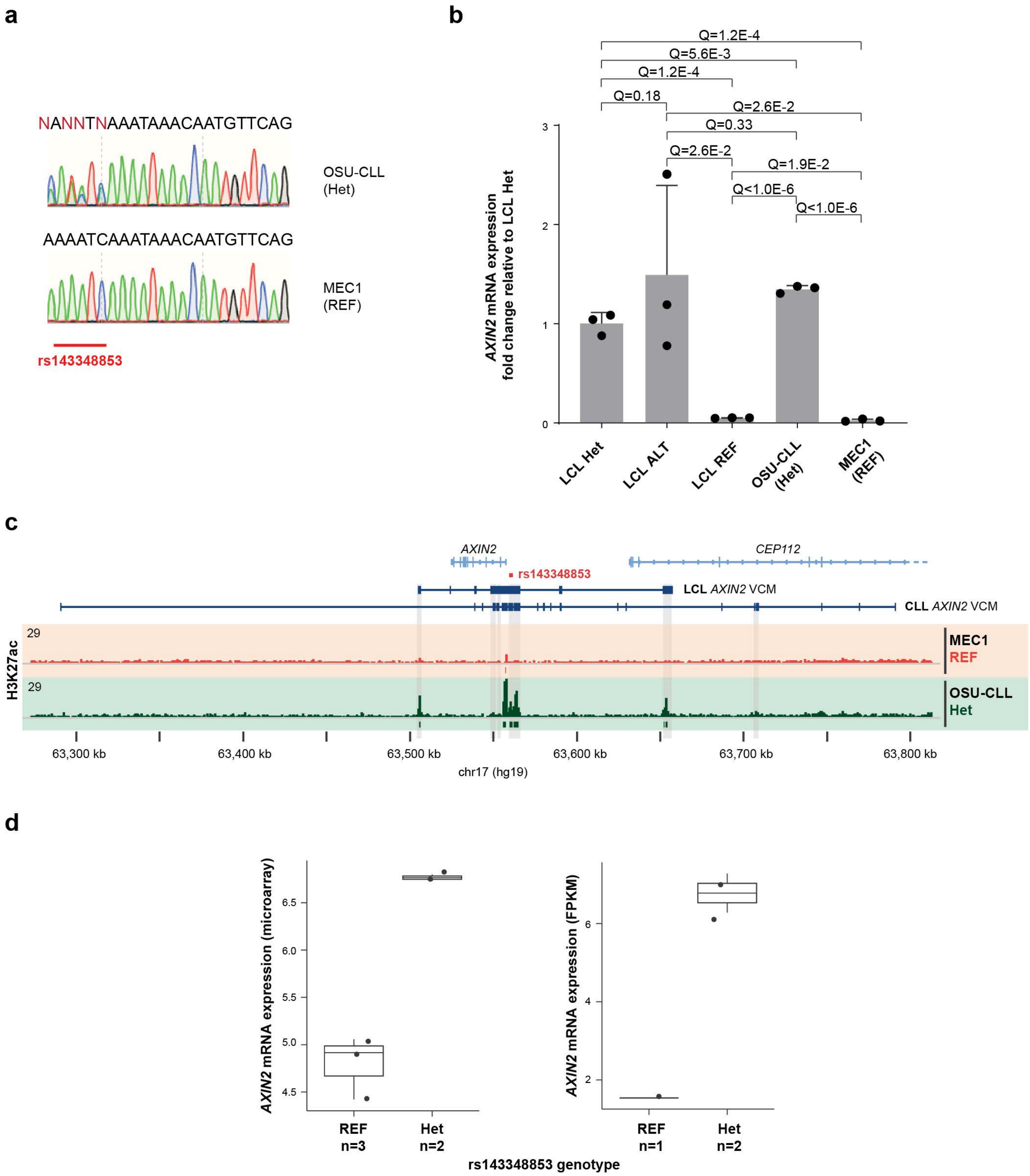

Supplementary Fig. 5. OSU-CLL and MEC1 *AXIN2* expression and enhancer composition. a Sanger sequencing results on the rs143348853 region from OSU-CLL and MEC1 genomic DNA. **b** *AXIN2* mRNA expression for the five cell lines studied in the project: LCL Het (GM12878), LCL homozygous REF (GM12282), GM11931 (homozygous ALT) and the two human CLL cell lines OSU-CLL and MEC1. Q indicate FDR corrected *p* value from an unpaired two-sided t-test with Welch's correction. Bar plots indicate mean ±SD for n = 3 biological replicates. Source data are provided as a Source Data file. **c** H3K27ac signal profile and peaks for the MEC1 and OSU-CLL cell lines with their respective rs143348853 genotypes listed. The illustrated VCMs represent the LCL and CLL VCMs from the pairwise Pearson correlation method. Grey boxes indicate LCL and CLL enhancers. **d** *AXIN2* mRNA expression (microarray or RNA-seq based) for the PCAWG CLL patients with EBV infection. n indicates the number of considered patients. Boxes indicate the IQR (25-75%) and the box center indicates the median. Whiskers represent de minimum or maximum values of no further than 1.5 times the IQR for both the top and bottom of the box.

Supplementary Fig. 6

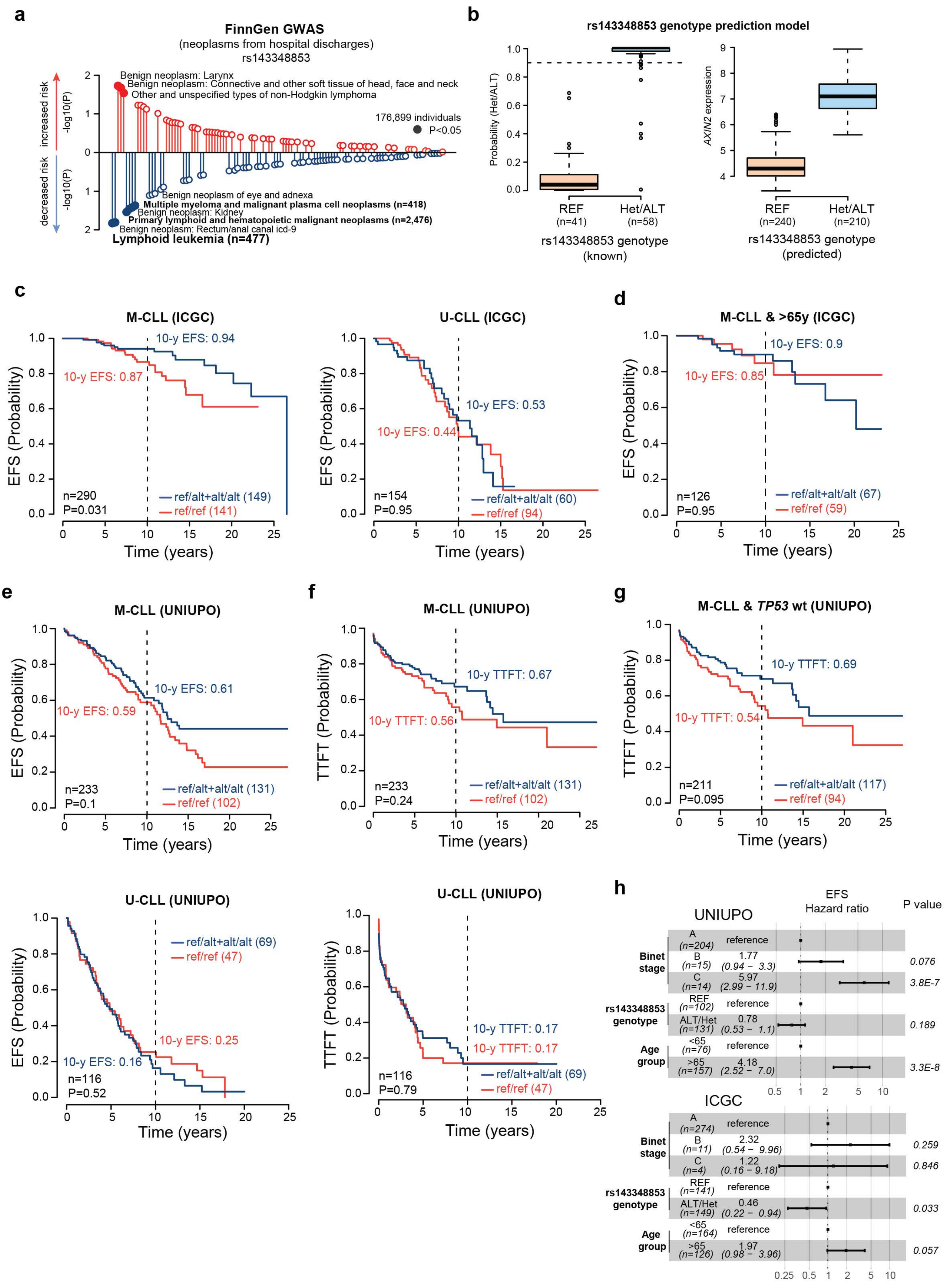

Supplementary Fig. 6. FinnGen and CLL clinical data. a Association between rs143348853 and benign/malignant neoplasms in FinnGen based on information from hospital discharges. *n* and P indicate the number of considered patients and nominal *p* value, respectively. **b** Logistic regression model to predict rs143348853 deletion (ref/alt + alt/alt) vs. non-deletion (ref/ref) carrier status from molecular CLL data in the ICGC CLL cohort. The left panel shows the deletion probability vs. known WGS-derived rs143348853 deletion carrier status. The right panel shows the relationship between inferred rs143348853 carrier status and *AXIN2* expression in the full ICGC CLL cohort. Boxes indicate the IQR (25-75%) and the box center indicates the median. Whiskers represent de minimum or maximum values of no further than 1.5 times the IQR for both the top and bottom of the box. **c** Association between rs143348853 deletion carrier status (ref/ref vs. ref/alt + alt/alt) and event-free survival (EFS) probability in M-CLL (left) and U-CLL (right). Data was obtained from the ICGC CLLE-ES project[6,7]. *P* values are based on log-rank tests for the two groups and the probability of 10-year EFS is indicated. **d** Same as **c** but considering >65 year-old M-CLL patients only. **e** Same as **c** but for the UNIUPO CLL cohort (M-CLL, top) (U-CLL, bottom). **f** Same as **e** but considering the association between rs143348853 and TTFT. **g** Same as **f** but only considering low-risk (wild-type *TP53*) M-CLL patients. **h** EFS hazard ratios for M-CLL patients from both CLL cohorts obtained from a Cox proportional hazards regression model using as variates the Binet stage, the rs143348853 genotype and the age group. *n* and P indicate the number of considered patients and *p* value respectively.

Supplementary Fig. 7

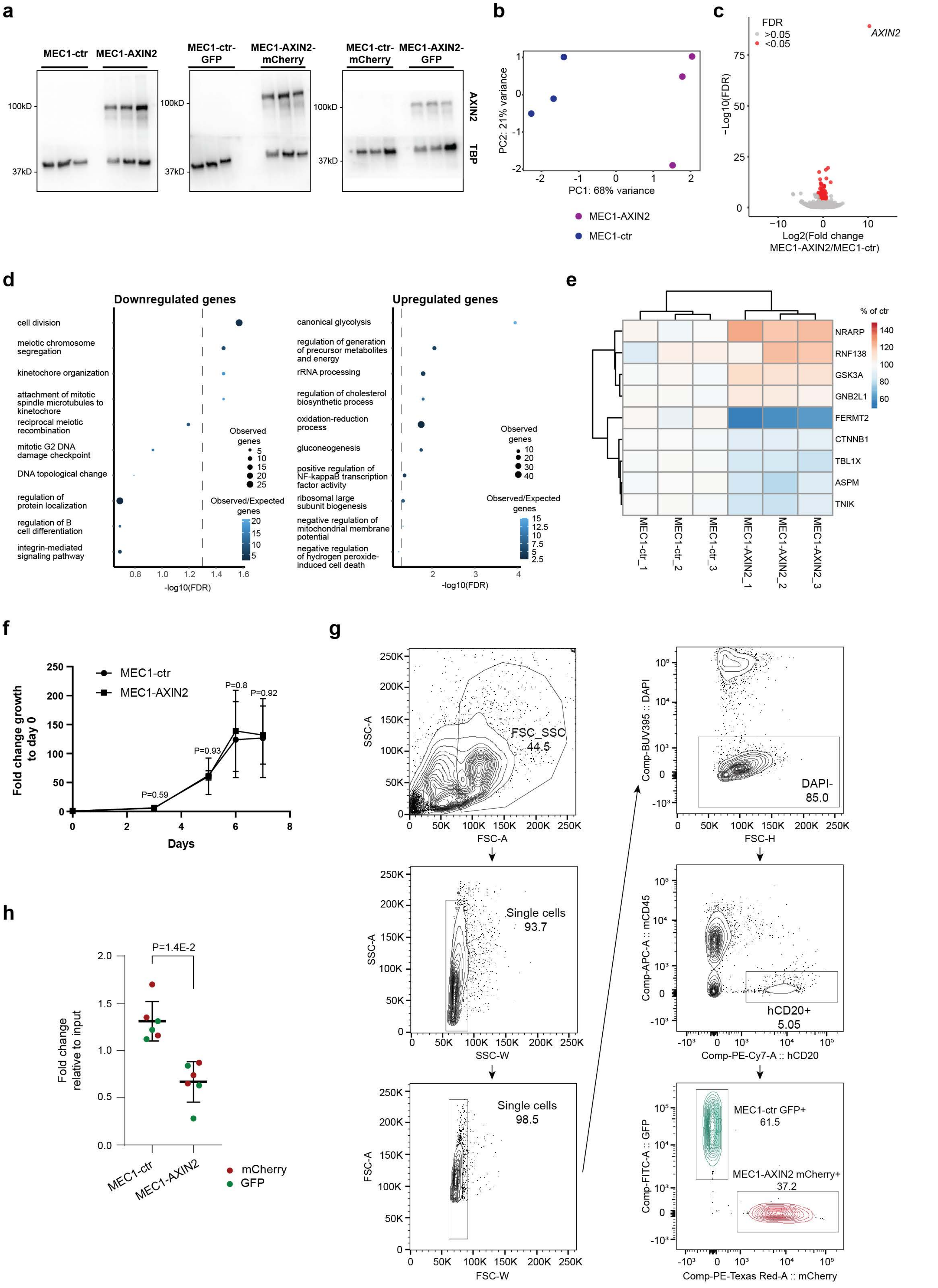

Supplementary Fig. 7. Effect of *AXIN2* (over)expression on MEC1 cells. a AXIN2 protein level assessment by western blot for the AXIN2-overexpressing MEC1 cells from 3 replicates sampled at different days (for GFP or mCherry labelled and unlabelled MEC1 cells). **b** PCA results from MEC1-ctr and MEC1-AXIN2 RNA-seq data from three biological replicates. **c** Volcano plot of the log2 fold gene expression change between MEC1-AXIN2 over MEC1-ctr cells; FDR < 0.05, differentially expressed genes are colored in red. **d** Gene ontology analysis results based on the above-described RNA-seq data, considering significantly differentially expressed genes (FDR < 0.05 and fold change different than 1). **e** Expression of significantly differentially expressed Wnt pathway genes from the RNA-seq data. Values on the heatmap represent the FPKM percentage over the FPKM mean of the control samples (MEC1-ctr, columns) for each gene (row). Dendrogram clustering based on Euclidean distances. **f** *In vitro* growth curve of MEC1-AXIN2 and MEC1-ctr cells. The plot shows the fold change respective to day 0 mean ±SD values for the three biological replicates. Measurements were taken on days 3, 5, 6 and 7 after seeding. P indicates *p* value from a two-sided unpaired Welch corrected t-test. **g** Flow cytometry gating strategy on MEC1 cells extracted from the mouse bone marrow from the *in vivo* competition experiment. **h** Fold change of the final cell percentage (day 25-26) with respect to the input percentage (day 0) of MEC1-AXIN2 mCherry+, MEC1-ctr GFP+, MEC1-AXIN2 GFP+, and MEC1-ctr mCherry+ cells from the *in vivo* competitive experiment to test the effect of the fluorescent proteins on MEC1 cell proliferation *in vivo*; the mean ±SD across all 6 mice is displayed, *p* value calculated with a paired two-sided t-test. For **a** and **h**, source data are provided as a Source Data file.

Supplementary Fig. 8

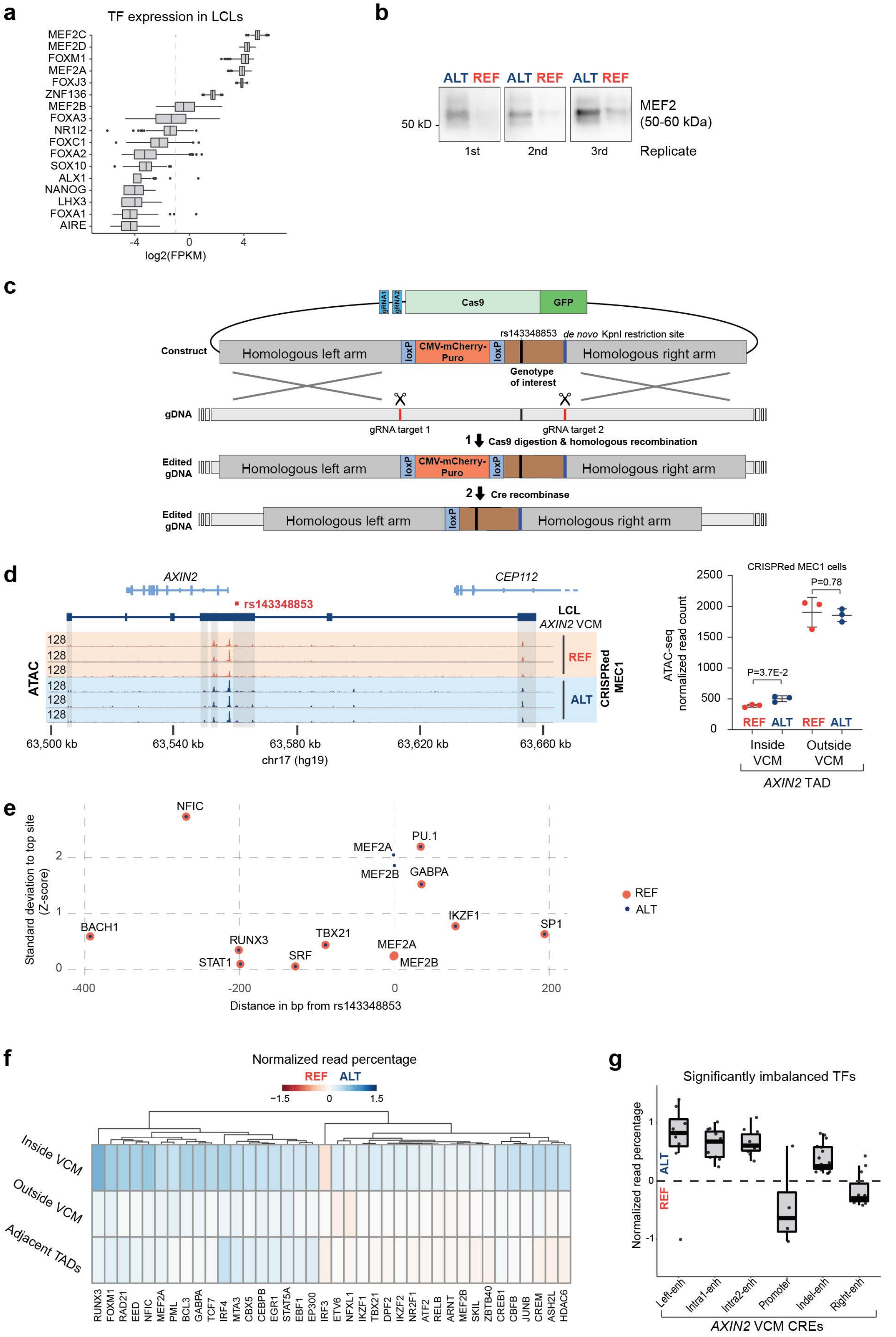

Supplementary Fig. 8. CRISPR/Cas9 experimental design and *AXIN2* VCM significantly imbalanced TFs. a mRNA expression levels of TF candidates identified in **Fig. 4a** from n = 327 individual LCLs. The dashed line represents the 0.5 FPKM threshold. Boxes indicate the IQR (25-75%) and the box center indicates the median. Whiskers represent de minimum or maximum values of no further than 1.5 times the IQR for both the top and bottom of the box. **b** *In vitro* DNA pulldown followed by WB with MEF2 antibody performed in three replicates. **c** Strategy to modify the MEC1 genotype with one single plasmid encoding all the required elements: Cas9-GFP, two gRNAs and the selection cassette containing the desired genetic alteration (REF, ALT, ALT.PU.1Δ or MEF2Δ) surrounded by the homologous arms. **d** ATAC-seq signal on the *AXIN2* VCM region of CRISPRed MEC1 cells for the n = 3 biological replicates (left) and the normalized read sum (mean ±SD) of all peaks embedded in or outside the *AXIN2* VCM (right). P indicates the p value of a two-sided t-test. **e** DNA motif Z-score distribution (i.e. standard deviation) across the indel-enhancer of TFs that appeared significantly imbalanced on the indel-enhancer (in addition to PU.1). **f** Percentage of ALT reads of significantly imbalanced TFs in the *AXIN2* LCL VCM region. Dendrogram clustering based on Euclidean distances. The percentage of reads for each TF is shown for: inside, outside VCM and adjacent TADs for comparison purposes. **g** Percentage of ALT reads from significantly imbalanced TFs for each CRE embedded in the *AXIN2* LCL VCM: left-enhancer (n = 8), intra1-enhancer (n = 11), intra2-enhancer (n = 10), promoter (n = 4), indel-enhancer (n = 21) and right-enhancer (n = 11), where n indicates significantly imbalanced TFs. Boxes indicate the IQR (25-75%) and the box center indicates the median. Whiskers represent de minimum or maximum values of no further than 1.5 times the IQR for both the top and bottom of the box. In **f** and **g**, the percentage of ALT associated reads is displayed as the log2 fold change of ALT read percentage of a TF over the ALT read percentage of the input. For **b** and **d**, source data are provided as a Source Data file.

Supplementary Fig. 9

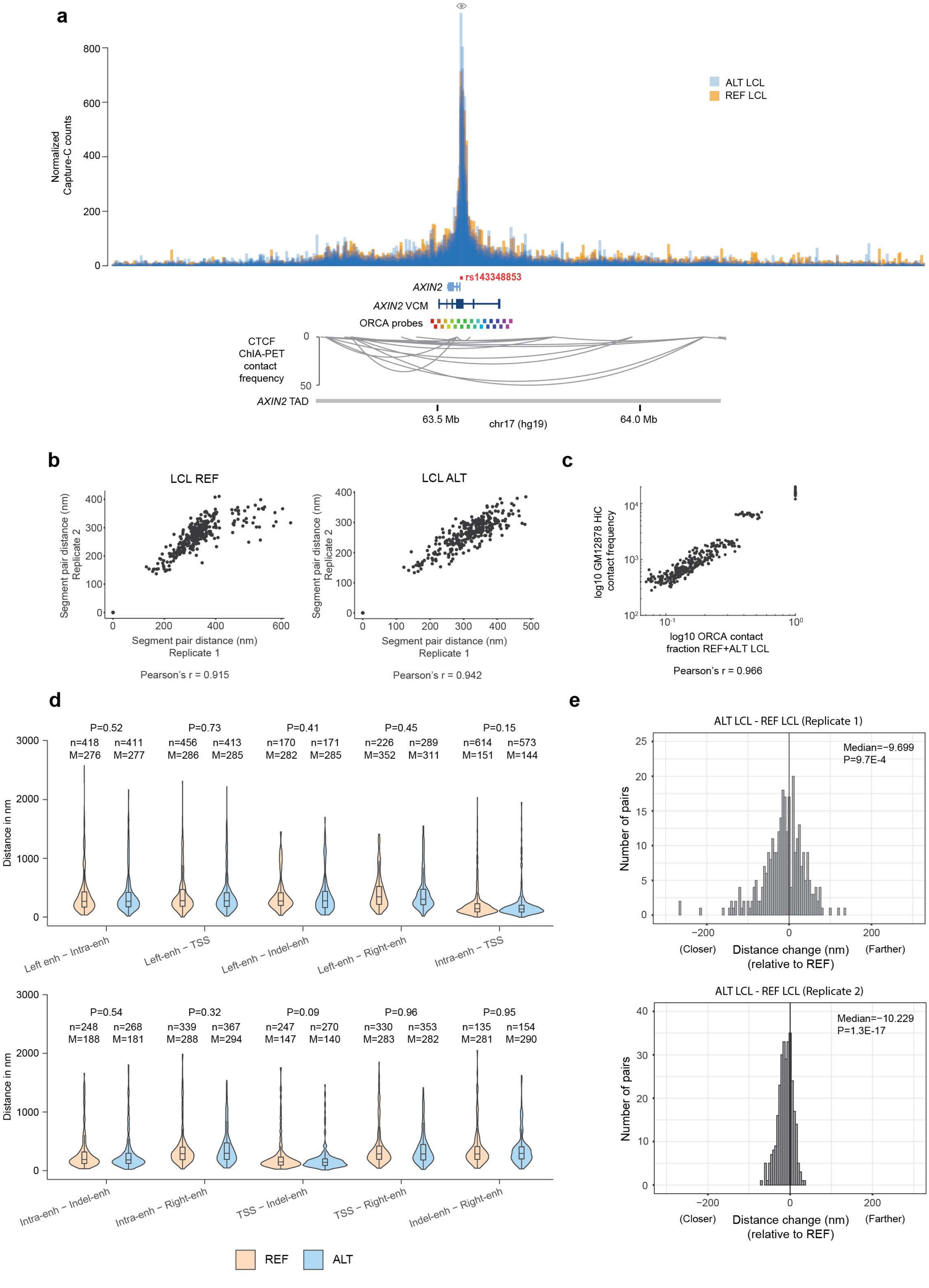

Supplementary Fig. 9. Chromatin architecture characteristics of the *AXIN2* VCM region. **a** Histogram of the TAD-normalized Capture-C counts from merged replicates for the ALT and REF LCLs. The viewpoint is marked with an eye (indel). Below, the GM12878 *AXIN2* TAD[2] and the GM12878 CTCF ChIA-PET interactions[8] are also displayed for comparison purposes. **b** Scatter plot to illustrate the correlation between ORCA replicate 1 and 2 for both LCLs. **c** Scatter plot to illustrate the correlation between the log10 ORCA contact fraction <150 nm of merged REF and ALT LCL data and the log10 GM12878 HiC contact frequency. **d** Distribution of the distance (nm) on REF and ALT LCLs between ORCA segments corresponding to LCL *AXIN2* CREs. P indicates nominal *p* values from a two-sided Wilcoxon test. *n* and M represent the number of considered single cells and population-median distance, respectively. Boxes indicate the IQR (25-75%) and the box center indicates the median. Whiskers represent de minimum or maximum values of no further than 1.5 times the IQR for both the top and bottom of the box. **e** Histograms showing the differences between pairs of ORCA segments of population-median distances of REF and ALT alleles for each replicate. P indicates *p* value from a two-sided binomial test.

Supplementary Table 1. LCL *AXIN2* VCM and ChIP-seq peaks regions. *AXIN2* VCM information using the data from Waszak et al. 2015[3] and Delaneau et al. 2019[4]. The consensus *AXIN2* VCM is the merged set of both VCMs. ChIP-seq peak genomic coordinates (1-based) for all molecular phenotypes and, PU.1 and RPB2 data are from Waszak et al. 2015[3]. H3K27ac, H3K4me1 and H3K4me3 ChIP-seq LCL extended data from Delaneau et al. 2019[4]. In bold, the peaks that overlap with the indel. In red, the peaks that overlap with a molecular phenotype of the consensus *AXIN2* VCM but did not pass the 0.1% FDR or 0.5 Pearson's r threshold from the pairwise Pearson correlation themselves. Coordinates are for hg19. Feature name as described in this publication. The effect size of the ALT allele (Beta) and nominal *p* value of the association are based on a linear regression model.

Id	Chr	Start	End	LCL *AXIN2* VCM feature	Beta	*P* value
**LCL *AXIN2* VCM regions** (Pairwise Pearson correlation FDR < 0.1%, Waszak et al., 2015 data)						
10748	17	63504776	63506884	Left-enhancer	**3.57**	**1.8E-8**
10748	17	63524026	63524225	TTS		
10748	**17**	**63548330**	**63566297**	Intra-enhancer, Promoter and **Indel-enhancer**		
10748	17	63651549	63657768	Right-enhancer		
**LCL *AXIN2* VCM regions** (Pairwise Pearson correlation r > 0.5, Delaneau et al., 2019 data)						
6987	17	63504776	63506884	Left-enhancer	**4.56**	**3.7E-56**
6987	17	63538483	63540256	H3K4me1 satellite		
**6987**	**17**	**63548330**	**63566297**	Intra-enhancer, Promoter and **Indel-enhancer**		
6987	17	63589645	63591417	H3K4me1 satellite		
6987	17	63651549	63657768	Right-enhancer		
***AXIN2* VCM enhancer regions** (H3K27ac and H3K4me1 overlap)						
	17	63504798	63506884	Left-enhancer		
	17	63548904	63550765	Intra1-enhancer		
	17	63552328	63553582	Intra2-enhancer		
	**17**	**63557838**	**63566239**	**Indel-enhancer**		
	17	63651879	63655493	Right-enhancer		
**H3K27ac**						
24440	17	63504798	63506934	Left-enhancer	0.23	6.9E-11
22915	17	63548904	63550765	Intra1-enhancer	0.5	4.9E-27
23484	17	63552328	63553582	Intra2-enhancer	0.64	1.1E-22
**19115**	**17**	**63555248**	**63566297**	Promoter, **Indel-enhancer**	**1.66**	**7.0E-41**

19105	17	63651879	63655493	Right-enhancer	0.47	1.6E-10
**H3K4me3**						
10908	17	63552286	63558834	Promoter	2.24	6.6E-53
**H3K4me1**						
25114	17	63504776	63506884	Left-enhancer	0.73	3.3E-24
30220	17	63538483	63540256	H3K4me1 satellite	0.47	2.8E-20
30676	17	63548330	63554547	Intra-enhancer	1.88	4.3E-40
**23323**	**17**	**63557838**	**63566239**	**Indel-enhancer**	**2.65**	**2.1E-48**
32375	17	63589645	63591417	H3K4me1 satellite	0.49	1.3E-18
29144	17	63651549	63657768	Right-enhancer	0.9	2.1E-20
**PU.1**						
218	17	63505247	63505446	Left-enhancer	0.61	7.4E-03
**1036**	**17**	**63560245**	**63560444**	**Indel-enhancer**	**1.28**	**4.3E-12**
793	17	63563833	63564032	Indel-enhancer	0.98	3.4E-06
2462	17	63565183	63565382	Indel-enhancer	0.64	5.1E-03
662	17	63653599	63653798	Right-enhancer	0.55	1.7E-02
**RPB2**						
10938	17	63524026	63524225	TTS	0.97	4.6E-06
1989	17	63557149	63557348	Promoter	1.03	7.5E-07
3767	17	63557594	63557793	Promoter	1.10	7.4E-08

Supplementary Table 2. ***AXIN2* VCM ATAC-seq peaks information.** ATAC-seq peaks overlapping the LCL *AXIN2* VCM from Kumasaka et al., 2018[9]. Data obtained from the publicly available data files Peaks.bed.gz and lead_caQTL_variants.tsv.gz. All peaks have a probability of ~1 that rs143348853 is the chromatin accessibility QTL (caQTL) considering the peak as a caQTL (as determined by their P_Lead values). In bold, the peak that overlaps with the indel. Peak id as denoted in the original publication. Genomic coordinates (1-based) are for hg19. Beta: effect size of the ALT allele. PMR: probability that the peak is a master regulator.

Peak id	Chr	Start	End	Beta	PMR	LCL *AXIN2* VCM feature
254424	17	63505100	63505624	0.30929	0	Left-enhancer
254425	17	63506043	63506616	0.290475	0	Left-enhancer
254426	17	63549291	63550123	0.45857	0	Intra-enhancer1
254427	17	63552372	63553104	0.302549	0	Intra-enhancer2
254428	17	63553326	63553847	0.608001	0	Intra-enhancer2
254429	17	63556907	63558087	0.35991	0	Promoter
**254430**	**17**	**63559819**	**63560574**	**0.871128**	**0.997**	**Indel-enhancer**
254431	17	63561327	63561856	0.503294	0	Indel-enhancer
254432	17	63563620	63564228	0.798453	0	Indel-enhancer
254433	17	63564898	63565968	0.760568	0	Indel-enhancer
254434	17	63652737	63653484	0.192701	0	Right-enhancer

Supplementary Table 3. **Oligonucleotide sequences.**

**Sanger seq primers**	**Forward (5'-3')**	**Reverse (5'-3')**
AXIN2gt	ACCCCAGGAGTGCCAAGAGTAAA	TGACCGAGAAGATCCAAACCAAA
**qPCR primers**	**Forward (5'-3')**	**Reverse (5'-3')**
AXIN2_mRNA	AAGTGCAAACTTTCGCCAAC	ACAGGATCGCTCCTCTTGAA
HPRT1_mRNA	TGAGGATTTGGAAAGGGTGT	AATCCAGCAGGTCAGCAAAG
**Genotyping**	**Forward (5'-3')**	**Reverse (5'-3')**
rs143348853.genotype	ATAAAAGTGTCTACCATATAAACAA	TTATTATTGACCGAGAAGATC
**Cloning gRNAs**	**Forward (5'-3')**	**Reverse (5'-3')**
gRNA1	CACCGCCACAGAGGGTGTTATTAC	AAACGTAATAACACCCTCTGTGGC
gRNA2	CACCGCTTGCTACAGTCGCAGCCAT	AAACATGGCTGCGACTGTAGCAAGC
doublegRNA	CTGCAGACAAATGGCTCTAGAGGTACCGAGGGCCTATTTCCCATGATT	CCATTTACCGTAAGTTATGTAACGGGTACC
**CRISPR genotyping primers**	**Forward (5'-3')**	**Reverse (5'-3')**
Left_Right.gt	GCTGGCCATAAGACCCTCGT	CCCAGGGAGCCCTTAGTCCT
CRISPR.background	CGCAGGAACCCCTAGTGATG	AGAACGTGGACTCCAACGTC
**SNP pulldown probes**	**Forward (5'-3')**	**Reverse (5'-3')**
ALT_SNPpulldown (39bp)	biotin-GAAAAATCAAAACATCTAAAAATAAACAATGTTCAGAAA	TTTCTGAACATTGTTTATTTTTAGATGTTTTGATTTTTC
REF_SNPpulldown (44bp)	biotin-GAAAAATCAAAACATCTAAAATCAAAATAAACAATGTTCAGAAA	TTTCTGAACATTGTTTATTTTGATTTTAGATGTTTTGATTTTTC
**ChIP-qPCR**	**Forward (5'-3')**	**Reverse (5'-3')**
Negative control	GGTCATGCTGGTCTCGAACT	ATCCTTCCCATGGAACACAG
Indel region	AACTTGGTCTTTGTGGGTGGT	GTCCTTTGGTTAAAGTTGAGCCT

Positive control	CACACGAACCTTCCACGAG	TCGTTCAGCTTTGTCTGACG
**Luciferase assay**	**Forward (5’-3’)**	**Reverse (5’-3’)**
luciferase_rs143348853	AAATCGATAAGGATCCACCCCAGGAGTGCCAAGAGTAAA	TATCAGGGTTACTAGTTGACCGAGAAGATCCAAACCAAA
Luciferase_SNPcorrection_1stpiece	ACAGAAATGTAGACAGAGGGGTA	luciferase_rs143348853_Rev
Luciferase_SNPcorrection_2ndpiece	luciferase_rs143348853_For	CCCTCTGTCTACATTTCTGTTCCA
**NGS Capture-C**	**Left end indel fragment (5’-3’)**	**Right end indel fragment (5’-3’)**
Capture oligos for the rs143348853 viewpoint	biotin-CATGGACCCTCTTAGCCACCAGCTCTGAGCTGGCCCAGGGCCAAGAACACAGCCACCACCTTTGGCCACCCCAGGAGTGCCAAGAGTAAAACTGTCACTGTGGTTCCAGGGAGTCTTTGG	biotin-TCTTTGTGGGTGGTTTTCCTCTCCTCCCAGTTGTCCCCACCGACCCGTCACTGCCTCCCCCAACCAGCCCTAATCACTGTAGGCTCAACTTTAACCAAAGGACTACCTCATTATCCCATG
***AXIN2* ORF cloning**	**Forward (5’-3’)**	**Reverse (5’-3’)**
AXIN2.ORF_1stpiece	TCGTGAGGATCCGCCACCATGAGTAGCGCTATG	AAGAGACAGGCATGGGTTTGGTG
AXIN2.ORF_2ndpiece	CAAACCCATGCCTGTCTCTTCCA	GAATTCACGCGTTCAATCGATCCGCTCCACTT
**GFP cloning**	**Forward (5’-3’)**	**Reverse (5’-3’)**
pLV_GFP	AGAGGATCCGCCGCCACCATGGTGAGCAAGGGCG	ATTGTCGACTTACTTGTACAGCTCGTCCATGCC

Supplementary Table 4. **Donor template construct used for CRISPR/Cas9.** DNA construct used as template for the recombination step during genome editing, integrated in the same plasmid harboring the Cas9-GFP and the dual gRNA cassette (**Supplementary Fig. 8c**). The sequence here corresponds to the REF allele. ALT, ALT.PU.1Δ and MEF2Δ donor template constructs have the same sequence except for the corresponding genotype change (see **Fig. 4e**).

gccttcagaactttaaaacatgcaagaattgtggctctatctaggtatccatagaaaaagagaaggaagaggctgggtgtggt
ggctcacccgtgtaatcccagcacttcgggaggccaaggcaggcggatcacctgaggtcaggcattcgagaccagcctgg
ccaacatagtgaaaccctgtccctactaaaactacaaatattagccaggcgtggtgggggcgcctgtagtcccagctactcg
ggaggctgaggcaggagaatcatttaaacacaggaggttaaggttgcagtgagcctgggcgacagagcaagactccatct
caaaaaaaaaaaagaaaagaaaaaaagaggaggaagatccaccacccatgatctgctggaaaggggcaggtggcag
gactgtgcgccacctgccctcagcctaagggactgtgacaagggactagaaagctcttagactttctagtctaagactctgga
ctctggctcgtgggtgccatgacaggtggccgctcctccccaaaacctgcctttcggtgcccatggtctgcagctccaggctctc
gaccccaggcagccaggttggcataaagacagcagtaccagccctgaaatgtggccctgaagccagccaggaaggcca
gggaaagagtgtggcccgatgactcatctttgccttaggacaactgctggtgtgaggaaaccagtgggctctaaaagccccg
gcctgctggtattctgggggtcagtggcccatggaccctcttagccaccagctctgagctggcccagggccaagaacacag
ccaccacctttggccaccccaggagtgccaagagtaaaactgtcactgtggttccagggagtctttgggccacagagggtgtt
attacataacttcgtatagcatacattatacgaagttatacatgtgacattgattattgactagttattaatagtaatcaattacgggg
tcattagttcatagcccatatatggagttccgcgttacataacttacggtaaatggcccgcctggctgaccgcccaacgacccc
cgcccattgacgtcaataatgacgtatgttcccatagtaacgccaatagggactttccattgacgtcaatgggtggagtatttacg
gtaaactgcccacttggcagtacatcaagtgtatcatatgccaagtacgccccctattgacgtcaatgacggtaaatggcccgc
ctggcattatgcccagtacatgaccttatgggactttcctacttggcagtacatctacgtattagtcatcgctattaccatggtgatg
cggttttggcagtacatcaatgggcgtggatagcggtttgactcacggggatttccaagtctccaccccattgacgtcaatggga
gtttgttttggcaccaaaatcaacgggactttccaaaatgtcgtaacaactccgccccattgacgcaaatgggcggtaggcgtg
tacggtgggaggtctatataagcagagctcgtttagtgaaccgtcagatcgcctggagacgccatccacgctgttttgacctcc
atagaagacaccgactctactagaggatctgccaccatggtgagcaagggcgaggaggataacatggccatcatcaagg
agttcatgcgcttcaaggtgcacatggagggctccgtgaacggccacgagttcgagatcgagggcgagggcgagggccgc
ccctacgagggcacccagaccgccaagctgaaggtgaccaagggtggccccctgcccttcgcctgggacatcctgtcccct
cagttcatgtacggctccaaggcctacgtgaagcaccccgccgacatccccgactacttgaagctgtccttccccgagggctt
caagtgggagcgcgtgatgaacttcgaggacggcggcgtggtgaccgtgacccaggactcctccctgcaggacggcgagt
tcatctacaaggtgaagctgcgcggcaccaacttcccctccgacggccccgtaatgcagaagaagaccatgggctgggag
gcctcctccgagcggatgtaccccgaggacggcgccctgaagggcgagatcaagcagaggctgaagctgaaggacggc
ggccactacgacgctgaggtcaagaccacctacaaggccaagaagcccgtgcagctgcccggcgcctacaacgtcaaca
tcaagttggacatcacctcccacaacgaggactacaccatcgtggaacagtacgaacgcgccgagggccgccactccacc
ggcggcatggacgagctgtacaagccccgggagggcagaggaagtcttctaacatgcggtgacgtggaggagaatcccg
gccctcgaaccgagtacaagcccacggtgcgcctcgccacccgcgacgacgtccccagggccgtacgcaccctcgccgc
cgcgttcgccgactaccccgccacgcgccacaccgtcgatccggaccgccacatcgagcgggtcaccgagctgcaagaa
ctcttcctcacgcgcgtcgggctcgacatcggcaaggtgtgggtcgcggacgacggcgccgcggtggcggtctggaccacg
ccggagagcgtcgaagcgggggcggtgttcgccgagatcggcccgcgcatggccgagttgagcggttcccggctggccgc
gcagcaacagatggaaggcctcctggcgccgcaccggcccaaggagcccgcgtggttcctggccaccgtcggcgtctcgc
ccgaccaccagggcaagggtctgggcagcgccgtcgtgctccccggagtggaggcggccgagcgcgccggggtgcccg
ccttcctggagacctccgcgccccgcaacctccccttctacgagcggctcggcttcaccgtcaccgccgacgtcgaggtgccc
gaaggaccgcgcacctggtgcatgacccgcaagcccggtgcctaatagctagctggaatcaacctctggattacaaaatttg
tgaaagattgactggtattcttaactatgttgctccttttacgctatgtggatacgctgctttaatgcctttgtatcatgctattgcttcccg
tatggctttcattttctcctccttgtataaatcctggttgctgtctctttatgaggagttgtggcccgttgtcaggcaacgtggcgtggtgt
gcactgtgtttgctgacgcaacccccactggttggggcattgccaccacctgtcagctcctttccgggactttcgctttccccctcc
ctattgccacggcggaactcatcgccgcctgccttgcccgctgctggacaggggctcggctgttgggcactgacaattccgtg

gtgttgtcggggaagctgacgtcctttccatggctgctcgcctgtgttgccacctggattctgcgcgggacgtccttctgctacgtc
ccttcggccctcaatccagcggaccttccttcccgcggcctgctgccggctctgcggcctcttccgcgtcttcgccttcgccctca
gacgagtcggatctccctttgggccgcctccccgcgctcgctgatcagcctcgactgtgccttctagttgccagccatctgttgttt
gcccctcccccgtgccttccttgaccctggaaggtgccactcccactgtcctttcctaataaaatgaggaaattgcatcgcattgt
ctgagtaggtgtcattctattctggggggtggggtggggcaggacagcaagggggaggattgggaagacaatagcaggcat
gctggggatgcggtgggctctatggggacctataacttcgtatagcatacattatacgaagttattggaacagaaatgtagac
agaggggtatggattgccataaaagtgtctaccatataaacaacagtaaagtcacacacacacacacacacacacacaca
cacacacacacacacaatgtggggggtgggggagagagaaaaatcaaaacatctaaaatcaaaataaacaatgttca
gaaaaaagaggttttcaaaaaggaagtggaaacttggtctttgtgggtggttttcctctcctcccagttgtccccaccgacccgtc
actgcctcccccaaccagccctaatcactgtaggctcaactttaaccaaaggactacctcattatcggtaccctgcgactgtag
caagaggggactgggactgggactggggacccaggagcaaggcccggtttggtttggatcttctcggtcaataataactgct
ctgagggccggggtgcagtggctcatgcctgtaatcccagcactttgggaggccaaggcaggtggatcacctgaggttagga
gttcgagaccagcctgaccgacatggagaaactccttctctactaaaaatacaaaattagtcaggcatggtggcgcatacctg
taatcccagctacttgagaggcggaggcaggagaatcgcttgaacccaggagacggaggttgtggtgagccgagatcgtg
ccattgcactccagcctgggcaacaagagcaaactccgtctcaaaaaataaataaataaaaaataactcctctgagagcat
ggtttccagaacaggtgctcgggtcactttagggcagtgactccctccaccatcacccctggcctgtcccctttcctacagctttat
atctggtgggacacttccctcccagcaggaggctgggccaagtactggaaagccctctctctagcctctgggtctctgactactt
gttgataatatttctagaggagaatccaaagtcctgtgcttatagcagcccgtttctcaagatagcacgttttccattttccccttgtc
cctggcagcagcgctatcatgtgacttgttgtaatcctgcacggttgcctggaaactggaaagcaaggtgatggatttctgcaca
tgctcactcgcccccccgctccacctttaaagaaaaaccctacggaggataaacagtccactttgcctaagtgcagacaagtt
taataaagcagagagtgattctttgctggaattatatactgggctgaaatc

Supplementary Table 5. **ORCA segment coordinates.** Genomic coordinates (1-based, hg19) for the 25 8-kb segments used for ORCA.

Chromosome	Start	End	Segment	LCL *AXIN2* VCM feature
chr17	63486119	63494121	segment1	
chr17	63494123	63502125	segment2	
chr17	63502127	63510129	segment3	Left-enhancer
chr17	63510131	63518133	segment4	
chr17	63518135	63526137	segment5	TTS
chr17	63526139	63534141	segment6	
chr17	63534143	63542145	segment7	H3K4me1 satellite
chr17	63542147	63550149	segment8	Intra-enhancer
chr17	63550151	63558153	segment9	Intra-enhancer and TSS
chr17	63558155	63566157	segment10	Indel-enhancer
chr17	63566159	63574161	segment11	
chr17	63574163	63582165	segment12	
chr17	63582167	63590169	segment13	H3K4me1 satellite
chr17	63590171	63598173	segment14	H3K4me1 satellite
chr17	63598175	63606177	segment15	
chr17	63606179	63614181	segment16	
chr17	63614183	63622185	segment17	
chr17	63622187	63630189	segment18	
chr17	63630191	63638193	segment19	
chr17	63638195	63646197	segment20	
chr17	63646199	63654201	segment21	Right-enhancer
chr17	63654203	63662205	segment22	Right-enhancer
chr17	63662207	63670209	segment23	
chr17	63670211	63678213	segment24	
chr17	63678215	63686118	segment25	

SUPPLEMENTARY REFERENCES

1. Robinson, J. T. *et al.* Integrative genomics viewer. *Nat. Biotechnol.* **29**, 24–26 (2011).
2. Beekman, R. *et al.* The reference epigenome and regulatory chromatin landscape of chronic lymphocytic leukemia. *Nat. Med.* **24**, 868–880 (2018).
3. Waszak, S. M. *et al.* Population Variation and Genetic Control of Modular Chromatin Architecture in Humans. *Cell* **162**, 1039–1050 (2015).
4. Delaneau, O. *et al.* Chromatin three-dimensional interactions mediate genetic effects on gene expression. *Science* **364**, (2019).
5. Oakes, C. C. *et al.* DNA methylation dynamics during B cell maturation underlie a continuum of disease phenotypes in chronic lymphocytic leukemia. *Nat. Genet.* **48**, 253–264 (2016).
6. Ramsay, A. J. *et al.* Next-generation sequencing reveals the secrets of the chronic lymphocytic leukemia genome. *Clin. Transl. Oncol.* **15**, 3–8 (2013).
7. Puente, X. S. *et al.* Non-coding recurrent mutations in chronic lymphocytic leukaemia. *Nature* **526**, 519–24 (2015).
8. Tang, Z. *et al.* CTCF-Mediated Human 3D Genome Architecture Reveals Chromatin Topology for Transcription. *Cell* **163**, 1611–1627 (2015).
9. Kumasaka, N., Knights, A. J. & Gaffney, D. J. High-resolution genetic mapping of putative causal interactions between regions of open chromatin. *Nat. Genet.* 1–1 (2018) doi:10.1038/s41588-018-0278-6.
