# Supplementary material for: A leukemia-protective germline variant mediates chromatin module formation via transcription factor nucleation
Source: Nat Commun. 2022 Apr 19;13:2042. doi: 10.1038/s41467-022-29625-6 (PMC9018852; doi:10.1038/s41467-022-29625-6)
Supplement: Supplementary file 2 — Description of Additional Supplementary Files [file 41467_2022_29625_MOESM2_ESM.pdf]

Description of Supplementary Data Files

Supplementary Data 1. DNA pulldown mass spectrometry results. List of detected proteins, the log2 normalized peptide counts for the three replicates and the log2 peptide fold change of ALT over REF oligos; sorted by FDR from the differentially bound protein analysis. TFs are indicated (isTF column) according to Lambert et al., 2018[1] annotation.

Excel file.

Supplementary Data 2. TF allelic imbalance results. Read counts information, *p* value from a two-sided binomial test and corrected *p* value (FDR) for all TFs from the ENCODE GM12878 cell line for each tested region in **Fig. 5b,c,d** and **Supplementary Fig. 8f,g**. Total reads are the sum of ALT- and REF-rs143348853 haplotype reads. Values used for plotting are also shown (log2 fold change of ALT reads percentage of a TF over the ALT reads percentage of the input). TFs are sorted by FDR. TFs with less than 6 total reads were discarded from the statistical test (as indicated by NA). Input ChIP-seq reads for each region are indicated at the bottom.

Excel file.

Supplementary Data 3. ENCODE GM12878 ChIP-seq metadata. ChIP-seq fastq files downloaded and used to perform the TF density and allelic imbalance analyses.

Excel file.

Supplementary Data 4. ORCA barcode sequences with genomic coordinates. DNA probe sequences used to perform ORCA on the *AXIN2* VCM. Genomic coordinates denoted as hg19.

Excel file.

SUPPLEMENTARY REFERENCES

1. Lambert, S. A. *et al.* The Human Transcription Factors. *Cell* **172**, 650–665 (2018).
