# Supplementary material for: A leukemia-protective germline variant mediates chromatin module formation via transcription factor nucleation
Source: Nat Commun. 2022 Apr 19;13:2042. doi: 10.1038/s41467-022-29625-6 (PMC9018852; doi:10.1038/s41467-022-29625-6)
Supplement: Supplementary file 7 — Reporting Summary [file 41467_2022_29625_MOESM7_ESM.pdf]

nature research

Corresponding author(s): Bart Deplancke

Last updated by author(s): Feb 8, 2022

Reporting Summary

Nature Research wishes to improve the reproducibility of the work that we publish. This form provides structure for consistency and transparency in reporting. For further information on Nature Research policies, see our Editorial Policies and the Editorial Policy Checklist.

Statistics

For all statistical analyses, confirm that the following items are present in the figure legend, table legend, main text, or Methods section.

n/a	Confirmed	
☐	☒	The exact sample size (*n*) for each experimental group/condition, given as a discrete number and unit of measurement
☐	☒	A statement on whether measurements were taken from distinct samples or whether the same sample was measured repeatedly
☐	☒	The statistical test(s) used AND whether they are one- or two-sided<br>*Only common tests should be described solely by name; describe more complex techniques in the Methods section.*
☐	☒	A description of all covariates tested
☐	☒	A description of any assumptions or corrections, such as tests of normality and adjustment for multiple comparisons
☐	☒	A full description of the statistical parameters including central tendency (e.g. means) or other basic estimates (e.g. regression coefficient) AND variation (e.g. standard deviation) or associated estimates of uncertainty (e.g. confidence intervals)
☐	☒	For null hypothesis testing, the test statistic (e.g. *F*, *t*, *r*) with confidence intervals, effect sizes, degrees of freedom and *P* value noted<br>*Give P values as exact values whenever suitable.*
☒	☐	For Bayesian analysis, information on the choice of priors and Markov chain Monte Carlo settings
☒	☐	For hierarchical and complex designs, identification of the appropriate level for tests and full reporting of outcomes
☐	☒	Estimates of effect sizes (e.g. Cohen's *d*, Pearson's *r*), indicating how they were calculated

Our web collection on statistics for biologists contains articles on many of the points above.

Software and code

Policy information about availability of computer code

Data collection: BD FACSDiva software v8.0.2 was used for flow cytometry data acquisition.

Data analysis:
R v3.6.3 and v3.6.2
Matlab R2019a
bamCoverage v3.5.0
Picard v22.2.9
edgeR v3.28.1 and v3.26.8
HOMER v4.11
htseq-count v0.12.4
DESeq2 v1.26.0
Clomics v1.0
bwa mem v0.7.17
SAMtools v1.9
GenomicRanges v1.38.0
rtracklayer 1.46.0
Bedtools v2.27.1
vcf2diploid (https://github.com/abyzovlab/vcf2diploid)
freebayes v.1.3.4 (https://github.com/freebayes/freebayes)
Rank Ordering of Super-Enhancers (ROSE2) (https://github.com/BradnerLab/pipeline)
IGV v2.3.92
GenomicInteractions v1.16.0
Gviz v1.30.3
MACS2 v2.1.2

Proteome Discoverer v.2.4
DpnII2E.pl (https://github.com/Hughes-Genome-Group/captureC/releases)
dpngenome3_1.pl (https://github.com/Hughes-Genome-Group/captureC/releases)
CCanalyser3.pl (https://github.com/Hughes-Genome-Group/captureC/releases)
FLASH v1.2.11
Trim Galore v0.6.7 (https://www.bioinformatics.babraham.ac.uk/projects/trim_galore/)
BWA-MEM v.0.7.17-r1188
bigutilsr v0.3.4
limma v3.40.6
methrix v1.4.07
STAR v2.7.3a
TopGO v2.38.1
BiomaRt v2.42.1
pheatmap v1.0.12
metafor v3.0-2
GraphPad Prism 8
Bioconductor v3.10
FlowJo v10.7.1

For manuscripts utilizing custom algorithms or software that are central to the research but not yet described in published literature, software must be made available to editors and reviewers. We strongly encourage code deposition in a community repository (e.g. GitHub). See the Nature Research guidelines for submitting code & software for further information.

Data

Policy information about availability of data

All manuscripts must include a data availability statement. This statement should provide the following information, where applicable:

- Accession codes, unique identifiers, or web links for publicly available datasets
- A list of figures that have associated raw data
- A description of any restrictions on data availability

Data availability:
RNA-seq data from MEC1-AXIN2 overexpression or ctr MEC1 cells, ATAC-seq data from CRISPRed MEC1 cells and Capture-C data from LCLs have been deposited into the Gene Expression Omnibus (GEO) repository (accession number GSE162387).

The mass spectrometry proteomics data have been deposited to the ProteomeXchange Consortium via the PRIDE partner repository with the dataset identifier PXD029313.

Online resources and publicly available datasets:
E-MTAB-3657 (LCL ChIP-seq data)
E-MTAB-3656 (LCL RNA-seq data)
EGAS00001003485 (LCL ChIP-seq and RNA-seq)
E-MTAB-3657 (LCL ChIP-seq and RNA-seq)
ERP110508 (LCL ATAC-seq)
https://zenodo.org/record/1405945#.X7PXCMco-uM (processed LCL ATAC-seq data)
EGAD00001004046 (CLL Blueprint data)
ENSEMBL (https://www.ensembl.org/index.html)
Roadmap Epigenomics project (www.roadmapepigenomics.org)
ENCODE (https://www.encodeproject.org)
HOCOMOCO (https://hocomoco11.autosome.ru/)
The 1000 Genomes Project (https://www.internationalgenome.org/)
GM12878 phased genotype data from the Genome In a Bottle consortium (ftp-trace.ncbi.nlm.nih.gov/giab/ftp/release/NA12878_HG001/latest/GRCh37/)
ERP002168 (LCL CTCF ChIP-seq)
dbGaP Study Accession phs000178.v11.p8
International Cancer Genome Consortium (ICGC) (https://dcc.icgc.org/)
EGAD00001005970 (CLL whole genome bisulfite sequencing data)
EGAS00001000534 (healthy donor B cells Whole genome bisulfite sequencing data)
UCSC Genome Browser (https://genome.ucsc.edu/)
FinnGen (http://r4.finngen.fi/variant/17-65564173-AAAATC-A)
GTEx Portal (https://gtexportal.org/home/index.html)
GSM1872886 (GM12878 CTCF ChIA-PET)
GSE119744 (MEC1 and OSU-CLL ChIP-seq)
RegulomeDB (https://regulomedb.org/regulome-search/)
NCBI dbSNP build 154 (https://www.ncbi.nlm.nih.gov/snp/)
3D Genome Browser (http://3dgenome.fsm.northwestern.edu/tutorial.html)
TF list (Lambert et al., 2018, PMID: 29425488)
GM12878 TAD coordinates (Beekman et al., 2018, PMID: 29785028)
B cell developmental enhancers (Oakes et al., 2016, PMID: 26780610)
GPP sgRNA Designer tool from the Broad Institute on CRISPRko mode (https://portals.broadinstitute.org/gpp/public/).

Field-specific reporting

Please select the one below that is the best fit for your research. If you are not sure, read the appropriate sections before making your selection.

☒ Life sciences ☐ Behavioural & social sciences ☐ Ecological, evolutionary & environmental sciences

For a reference copy of the document with all sections, see nature.com/documents/nr-reporting-summary-flat.pdf

Life sciences study design

All studies must disclose on these points even when the disclosure is negative.

Sample size	Sample size calculation was not performed. At least 3 biological or technical replicates were obtained per experiment, based on standards in the field and to be able to perform statistical test (such as a t-test). Additional replicates were obtained when possible to increase the statistical power. For CRISPR, we aimed for >3 clones per genotype and we report results for all good clones obtained from two batches (ALT and REF) and one batch (ALT.PU.1 and MEF2Δ). For the in vivo experiment, we used 10 mice per batch for a total of two batches to have a value of Total N of mice - N of groups between 10 and 20 (Charan and Kantharia, 2013, PMID: 24250214).
Data exclusions	No data exclusions were performed during the course of this study.
Replication	In vitro DNA pulldown was performed in three technical replicates. ORCA was performed in two independent batches. NGS Capture-C and ATAC-seq were performed in three biological replicates. ChIP-qPCR was performed in six biological replicates (qPCR with three technical replicates). Luciferase assays were performed in four biological replicates. RT-qPCR for assessing AXIN2 expression in cell lines was performed in three technical replicates and three biological replicates. RT-qPCR for assessing AXIN2 expression in CRISPR clones was performed in three technical replicates for each clone (each clone is considered an independent biological replicate and >3 clones were aimed per genotype). RNA-seq was performed in three biological replicates. In vitro proliferation was performed in three biological replicates and five technical replicates. MEC1 in vivo competition experiment was performed in two batches of 10 mice each. All replicated experiments showed concordance (successful replication), except for some CRISPR clones, which showed some degree of heterogeneity.
Randomization	Randomization was performed during the mice experiment when selecting mice to be injected with the MEC1-AXIN2-mCherry/MEC1-ctr-GFP or the MEC1-AXIN2-GFP/MEC1-ctr-mCherry mixture. For the other experiments, randomization is generally not applied in this field (most of the experiments start from a single cell line or defined samples).
Blinding	The investigators were not blinded during the experiments and sample processing. The results of our experiments are obtained by objective quantitative methods.

Reporting for specific materials, systems and methods

We require information from authors about some types of materials, experimental systems and methods used in many studies. Here, indicate whether each material, system or method listed is relevant to your study. If you are not sure if a list item applies to your research, read the appropriate section before selecting a response.

Materials & experimental systems

n/a	Involved in the study	
☐	☒	Antibodies
☐	☒	Eukaryotic cell lines
☒	☐	Palaeontology and archaeology
☐	☒	Animals and other organisms
☐	☒	Human research participants
☒	☐	Clinical data
☒	☐	Dual use research of concern

Methods

n/a	Involved in the study	
☒	☐	ChIP-seq
☐	☒	Flow cytometry
☒	☐	MRI-based neuroimaging

Antibodies

Antibodies used	AXIN2 (#2151, Cell Signaling, clone 76G6), RRID:AB_2062432. For western blots at 1:1000 dilution.<br>TBP (C15200002, Diagenode). For western blots at 1:5000 dilution.<br>MEF2 (sc-313, Santa Cruz), RRID:AB_631920. For western blots at 1:500 dilution.<br>MEF2C (5030T, Cell Signaling), RRID:AB_10548759. For ChIP at 1:50 dilution.<br>anti-rabbit HRP (sc2004, Santa Cruz), RRID:AB_631746. For western blots at 1:5000 dilution.<br>anti-mouse HRP (#115-035-146, Jackson ImmunoResearch), RRID:AB_2307392. For western blots at 1:1000 dilution.<br>anti-human CD20 PE/Cy7 (#302312, BioLegend, clone 2H7, Lot B214749), RRID:AB_314260. For flow cytometry at 1:200 dilution.<br>anti-mouse CD45 APC (#17-0451-83, eBioscience, clone 30-F11, Lot E07149-1632), RRID:AB_469393. For flow cytometry at 1:800 dilution.
Validation	All antibodies were purchased from commercial vendors. Validation information can be found at the respective websites:

Validation	AXIN2: https://www.cellsignal.com/products/primary-antibodies/axin2-76g6-rabbit-mab/2151<br>TBP: https://www.diagenode.com/en/p/tbp-monoclonal-antibody-classic-100-ul<br>MEF2: https://www.scbt.com/p/mef-2-antibody-c-21<br>MEF2C: https://www.cellsignal.com/products/primary-antibodies/mef2c-d80c1-xp-rabbit-mab/5030?submissionGuid=58490922-7eeb-44be-bbe3-6b4262823a64<br>anti-rabbit HRP: https://www.scbt.com/es/p/goat-anti-rabbit-igg-hrp<br>anti-mouse HRP: https://www.jacksonimmuno.com/catalog/products/115-035-146<br>anti-human CD20 PE/Cy7: https://www.biolegend.com/en-us/products/pe-cyanine7-anti-human-cd20-antibody-1902?GroupID=BLG7904<br>anti-mouse CD45 APC: https://www.thermofisher.com/antibody/product/CD45-Monoclonal-Antibody-30-F11-APC-eBioscience/17-0451-83

Eukaryotic cell lines

Policy information about cell lines

Cell line source(s)	HEK293 (in-house), GM12878 (Coriell Institute), GM12282 (Coriell Institute), GM11931 (Coriell Institute), MEC1 (ACC 497, DSMZ) and OSU-CLL (The Ohio State University's Human Genetics Sample Bank).
Authentication	None of the cell lines were authenticated.
Mycoplasma contamination	Cell lines tested negative for mycoplasma contamination.
Commonly misidentified lines (See ICLAC register)	None.

Animals and other organisms

Policy information about studies involving animals; ARRIVE guidelines recommended for reporting animal research

Laboratory animals	NOD.Cg-Prkdcscid Il2rgtm1Wjl/SzJ, The Jackson Laboratory. Male, ~12-14 weeks old.
Wild animals	The study did not involve wild animals.
Field-collected samples	The study did not involve field-collected samples.
Ethics oversight	Mice were bred and maintained at the EPFL animal facility. All animal work was carried out in accordance with Swiss national guidelines. This study was reviewed and approved by the cantonal veterinary service, Vaud.

Note that full information on the approval of the study protocol must also be provided in the manuscript.

Human research participants

Policy information about studies involving human research participants

Population characteristics	The study is observational, retrospective and non interventional. The main clinical characteristic (i.e. age, gender, date of diagnosis and of date) of patients were collected anonymously.
Recruitment	The recruitment is: consecutive CLL patients referring to our institution.
Ethics oversight	The study was approved by our local Ethics Committee: Comitato Etico Interaziendale di Novara, Italy. Study number CE 8/11 and CE 120/19.

Note that full information on the approval of the study protocol must also be provided in the manuscript.

Flow Cytometry

Plots

Confirm that:

☒ The axis labels state the marker and fluorochrome used (e.g. CD4-FITC).

☒ The axis scales are clearly visible. Include numbers along axes only for bottom left plot of group (a 'group' is an analysis of identical markers).

☒ All plots are contour plots with outliers or pseudocolor plots.

☒ A numerical value for number of cells or percentage (with statistics) is provided.

Methodology

Sample preparation	Cells were harvested, washed twice with PBS and a mixture of 50:50 MEC1-ctr-GFP and MEC1-AXIN2-mCherry cells was prepared in PBS with a concentration of 5 million cells per 100 µl. 200 µl of this solution were injected intravenously by the tail vein into each mouse for a total of 10 NSG (NOD.Cg-Prkdcscid Il2rgtm1Wjl/SzJ, The Jackson Laboratory) ~12-14 weeks old

male mice per experiment. Prior to the injection we analyzed the percentage of the respective cell populations in the input by flow cytometry using the LSR Fortessa (BD Biosciences). After 26 days, the mice were sacrificed and bone marrow immune cells from both legs (femur and tibia) and hip bones were extracted. Single cell suspensions were prepared as previously described (Wilson et al., 2001, PMID: 11581321). Cells were stained with human CD20 PE/Cy7 (302312, BioLegend), mouse CD45 APC (17-0451-83, eBioscience) and DAPI, and analyzed by flow cytometry.

An additional experiment was performed by exchanging the fluorescent proteins. For this setup, we used 3 mice with a mixture of 50:50 MEC1-ctr-mCherry and MEC1-AXIN2-GFP, and 3 mice with a mixture of 50:50 MEC1-ctr-GFP and MEC1-AXIN2-mCherry. 25-26 days post injection, cells were stained with human CD20 PE/Cy7 (302312, BioLegend), mouse CD45 APC (17-0451-83, eBioscience) and Zombie NIR (BioLegend), and analyzed by flow cytometry.

Instrument

Flow cytometry data from the MEC1 cells input was acquired on an LSR Fortessa (BD) analyzer equipped with 5-lasers and 18-detectors. The configurations are especially adapted to the use of new generation fluorochromes such as Brilliant Violet and Brilliant UltraViolet dyes. Xenograft sorts were performed using an FACSAria III (BD) FACS sorter or assessed with a LSR Fortessa (BD) analyzer

Software

BD FACSDiva software v8.0.2 was used for flow cytometry data acquisition on the LSR Fortessa and the FACSAria III. For analysis of fcs files FlowJo v10.7.1 from TreeStar was used (FlowJo -https://www.flowjo.com/solutions/flowjo; RRID:SCR_008520).

Cell population abundance

The sorted and analyzed cell mCD45-CD20+ population subdivided into GFP+ vs mCherry+ populations ranging from anywhere between 5-35% related to the total cell population and depending in the progression of CLL. Analyses were performed until the least common population (GFP+ or mCherry+ cells) reach 20,000 events.

Gating strategy

For the sorts of each sample the gating strategy was as follows: room temperature - 100µm Nozzle - sorting mask set to purity - sorting purity greater than 98%:
FSC_SSC
Single Cells
DAPI or Zombie NIR negative
Exclude murine CD45 positive cells (anti-mouse CD45-APC)
Gate on human CD20-PE-Cy7 positive cells (this can be anywhere from 5% to 35% - depending on how the disease is progressing).
Then the CD20+ cells should be subdivided into mCherry+ (Sorting gate 1) and GFP+ (Sorting gate 2).

☒ Tick this box to confirm that a figure exemplifying the gating strategy is provided in the Supplementary Information.
